# Hybrid neural networks in the mushroom body drive olfactory preference in *Drosophila*

**DOI:** 10.1126/sciadv.adq9893

**Published:** 2025-05-30

**Authors:** Li-Shan Cheng, Ching-Che Charng, Ruei-Huang Chen, Kuan-Lin Feng, Ann-Shyn Chiang, Chung-Chuan Lo, Ting-Kuo Lee

**Affiliations:** ^1^ Department of Physics, National Tsing Hua University, Hsinchu 300043, Taiwan.; ^2^ Institute of Systems Neuroscience and Department of Life Science, National Tsing Hua University, Hsinchu 30013, Taiwan.; ^3^ Brain Research Center, National Tsing Hua University, Hsinchu 30013, Taiwan.; ^4^ Kavli Institute for Brain and Mind, University of California San Diego, La Jolla, CA 92093-0526, USA.; ^5^ Institute of Physics, Academia Sinica, Taipei 11529, Taiwan.; ^6^ Department of Biomedical Science and Environmental Biology, Kaohsiung Medical University, Kaohsiung 80780, Taiwan.; ^7^ Institute of Molecular and Genomic Medicine, National Health Research Institutes, Miaoli 35053, Taiwan.; ^8^ Graduate Institute of Clinical Medical Science, China Medical University, Taichung 40402, Taiwan.

## Abstract

In *Drosophila melanogaster*, olfactory encoding in the mushroom body (MB) involves thousands of Kenyon cells (KCs) processing inputs from hundreds of projection neurons (PNs). Recent data challenge the notion of random PN-to-KC connectivity, revealing preferential connections between food-related PNs and specific KCs. Our study further uncovers a broader picture—an L-shaped hybrid network, supported by spatial patterning: Food-related PNs diverge across KC classes, whereas pheromone-sensitive PNs converge on γ KCs. α/β KCs specialize in food odors, whereas γ KCs integrate diverse inputs. Such spatial arrangement extends further to the antennal lobe (AL) and lateral horn (LH), shaping a systematic olfactory landscape. Moreover, our functional validations align with computational predictions of KC odor encoding based on the hybrid connectivity, correlating PN-KC activity with behavioral preferences. In addition, our simulations showcase the network’s augmented sensitivity and precise discrimination abilities, underscoring the computational benefits of this hybrid architecture in olfactory processing.

## Teaser

An L-shaped hybrid network orchestrates differential olfactory coding for learning in flies, unveiling sensory-to-behavior links.

## INTRODUCTION

Olfaction is crucial for animal survival and reproductive success, enabling the precise distinction between food odors, pheromones, and other environmental odors. In the case of a fruit fly, *Drosophila melanogaster*, the mushroom body (MB) governs olfactory associative learning (*1*, *2*). This process commences with compressing olfactory signals from a multidimensional chemical space into a streamlined functional coding, orchestrated by ~52 glomeruli within the antennal lobe (AL) (*3*–*6*). These signals then diverge into projections, transmitting olfactory information from around 100 uniglomerular projection neurons (PNs) across the 52 glomeruli to sparsely activated Kenyon cells (KCs) from an assembly of roughly 2000 KCs (*7*–*9*). These olfactory cues subsequently acquire functional significance through altering connection weights between sparsely activated KCs and the MB output neurons (MBONs), thereby triggering actions through MBONs (*10*–*12*). Through processing in the triadic PN-KC-MBON hierarchical network, fruit flies distinguish distinct chemical compounds, generalize experiences to similar odors, and adeptly perceive their environment (*13*, *14*). However, the intricate neural circuitry translating odor representation from the AL glomeruli to KC coding underpinning the impressive functionality (*15*, *16*) of the fruit fly olfactory system remains an enigma.

Earlier investigations introduced two contrasting hypotheses concerning PN-to-KC connectivity: random versus structured (*17*–*28*). The random connectivity hypothesis posits random PN-to-KC connections, backed by functional response variations and a lack of precise cellular-level circuit specification (*20*, *24*). A specific type of KC exhibits varied reaction profiles to the same odor across different brains (*24*). For instance, Caron *et al.* (*20*) labeled inputs of 200 KCs through dye injection, discovering that no two KCs shared the same glomerular input combination. The spatial patterns of activated KCs display inconsistencies among animals, implying a random architecture (*24*). However, this seemingly random connectivity might result from experience-dependent network rewiring (*29*). For instance, olfactory responses in downstream MBONs exhibit higher interindividual correlation than expected by chance (*27*), and this variation diminishes in the *rutabaga* learning mutant (*11*).

In contrast, the structured connectivity hypothesis posits a degree of preferential connectivity between PNs and KCs at a population level, an arrangement maintained across individuals (*17*, *18*). This hypothesis finds support in various observations: (i) Functional imaging demonstrated consistent spatial calcium response patterns in KCs across diverse individuals for the same odor (*25*). (ii) Anatomical studies revealed notable overlap between specific glomerular PN axon arbors and dendritic arbors of particular KC classes (*17*, *28*). (iii) Neuronal spatial innervation of PNs could be clustered based on a metric assessing neuronal distance (*30*). (iv) Mutant flies without normal olfactory co-receptors display comparable glomerular input ratios to KC classes across diverse individuals (*21*), implying genetically determined PN-to-KC connectivity preferences. (v) The food-related PNs form a community structure converging on specific KCs, as demonstrated by comparisons mainly with specific random networks (*22*), where a KC claw can randomly choose any PN bouton using the FAFB dataset (*31*).

In addition to food-related PNs, previous anatomical observations indicate that a broader range of PNs exhibit substantial overlap with various KC classes (*17*). The possibility of additional, undiscovered structures remains an open question. Furthermore, it has yet to be confirmed whether these observed anatomical structures correspond to functional responses. To address these questions, we used a multimodal approach, integrating multiscale connectome analysis, functional experiments, and computational modeling using the hemibrain dataset (FlyEM) (*32*). The dataset offers nearly comprehensive synaptic-level connectome data, comprising 106 uniglomerular PNs and 1745 KCs. This heightened connectivity insight permits revisiting whether the calyx (CA) harbors concealed wiring preferences and how this structure influences olfactory information processing. Our results reveal connection preferences at various levels, from connections to spatial arrangements and from synapses to neurites, across different neuron types. Specifically, we identified a hybrid connectivity pattern between PN clusters and KC classes, characterized by varying degrees of convergence and divergence. Our examination of data incompleteness demonstrated its impact on observing this hybrid structure. Furthermore, using a quantification approach, we showed that the hybrid network is supported by the spatial arrangement of PN clusters and KC classes, a pattern that extends to the AL and lateral horn (LH). We validated this hybrid scheme through computer simulations that modeled KC responses to diverse odors, comparing these results with in vivo calcium imaging. Both spatial innervation analysis and functional predictions were reproduced using the FAFB dataset (*31*). In addition, beyond chemical odor features, we used correlation analysis to link odor tuning profiles with behavioral odor preferences, uncovering potential valence associations for each circuitry component. Furthermore, our computational model elucidates the potential advantages of this network for olfactory coding. Ultimately, our research highlights intricate connectivity patterns and functional interrelationships within the PN-to-KC hybrid network, illuminating the mechanisms guiding odor processing in the insect brain.

## RESULTS

### Diverse preferences in PN-to-KC connections

**Fig. 1.**
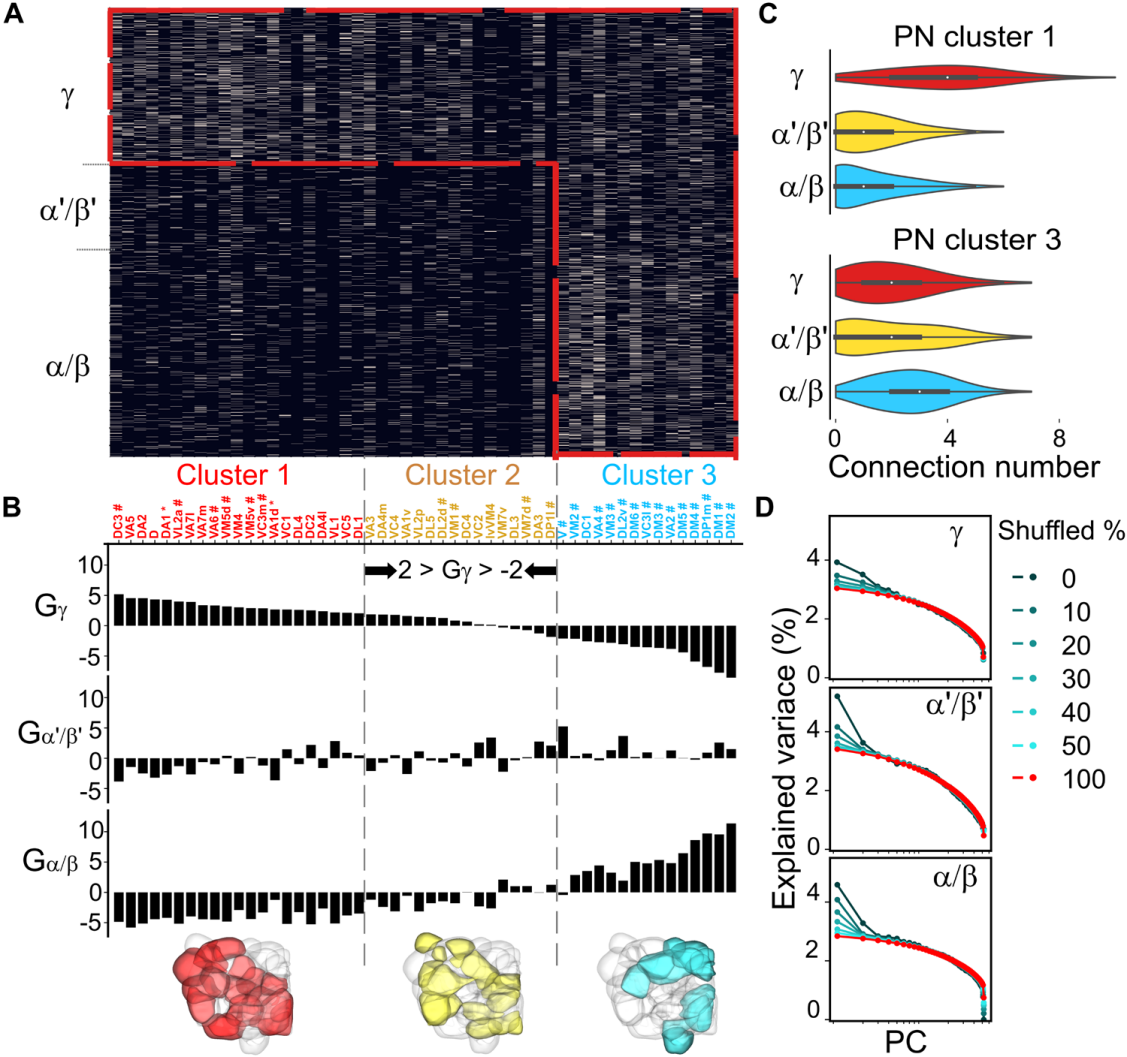
Preferential connectivity between olfactory PNs and three classes of KCs. (**A**) The connectivity matrix of PN-to-KC synapses derived from the hemibrain dataset (*32*) exhibits an L-shaped configuration. PNs originating from individual glomeruli were categorized into three clusters based on their connection preferences in (B). The color coding indicates the number of uniglomerular PNs connected to a particular KC. Note the hybrid pattern in which the PN cluster 3 projects to all KC classes (divergent) whereas PN cluster 1 projects predominantly to γ KC (convergent) (figs. S6 and S7) (**B**) Connection preferences indicated by the *G*_*i*_ score between a KC class (*i* = γ, α′/β′, or α/β) and PNs from a particular glomerulus (see Materials and Methods). The glomeruli are ordered by the *G*_γ_ score. Dashed lines indicate the range of *G*_γ_ scores of the shuffled results between −2 and 2. “#” indicates food-responsive glomeruli (*22*). “*” indicates pheromone-attractive glomeruli for a female fly (*69*). (**C**) Frequency distribution of PN numbers received by each individual neuron in three KC classes (see more details in fig. S2). (**D**) Analysis of global input preferences revealed by PCA on the PN-to-KC connection matrix after global shuffling of upstream PNs for individual KCs within the same class (refer to fig. S9 for detailed information). The color indicates the shuffled ratio of each KC class.

To unravel potential connection patterns of the PN-to-KC network within the calyx (fig. S1, A and B), we commenced by dissecting connectivity using the comprehensive hemibrain dataset (*32*) (Fig. 1A), an electron microscopy–based synaptic connectome derived from a female fly. Our examination of connection preferences (G) encompassing ~100 uniglomerular PNs related to olfaction and three distinct KC classes (γ, α′/β′, and α/β; see Fig. 1B) entailed a comparison of the real network with randomly shuffled networks. Because each glomerulus PN and its boutons may demonstrate disparate connection capacity (*33*), our shuffling algorithm preserved the connection number between individual PNs and KCs, maintaining the heterogeneous counts of KCs connected to a given PN’s bouton across different PN types (fig. S1, C and D; see Materials and Methods). The results of our analysis brought to light that each KC class exhibits preferred connections stemming from a specific subset of PNs (Fig. 1, B and C, and fig. S2). Specifically, the γ class features preferred (*G*_γ_ > 2) or disfavored (*G*_γ_ < −2) connections with 36 glomeruli (~69%), the α′/β′ class features preferred (*G*_α′/β′_ > 2) or disfavored (*G*_α′/β′_ < −2) connections with 19 glomeruli (~36%), and the α/β class features preferred (*G*_α/β_ > 2) or disfavored (G_α/β_ < −2) connections with 39 glomeruli (~75%) (Fig. 1B). Among 52 glomeruli, 39 exhibit both preference and disfavor toward specific KC classes, whereas 6 exhibit neither preference nor disfavor toward any KC class. Moreover, we observed a strong negative correlation between the preference score distributions of the γ and α/β classes (Pearson’s correlation coefficient = −0.96, *P* < 0.001). A similar trend emerges when considering connection weightings (synapse numbers) (fig. S3).

Next, we classified the 52 glomeruli into three distinct clusters based on the preference score *G*_γ_ (cluster 1: *G*_γ_ ≥ 2; cluster 2: 2 > *G*_γ_ > −2; cluster 3: *G*_γ_ ≤ −2; see Fig. 1B). The bouton connectivity of different clusters shows significant differences (fig. S1E), emphasizing the importance of maintaining the connection numbers of each bouton during the shuffling process. We found that glomeruli within the same cluster shared analogous functionality, with 93% of cluster 3 glomeruli (labeled by “#”) displaying responsiveness to food-related odors (Fig. 1B), consistent with a previous work done by Zheng *et al.* (*22*). In addition to food-related glomeruli, our analysis further revealed that more than one-third of glomeruli hold strong connection preference for γ KCs, including pheromone-attractive glomeruli for a female fly (labeled by “*”).

Comparing the hemibrain network with shuffled networks based on other random null models (*22*)—specifically, the random bouton model, which hypothesizes that each bouton has equal connection potential for KC claws, and the random glomerulus model, which assumes the same for each glomerulus—the connection preference from PN cluster 1 to γ KCs could not be observed (fig. S4) as these models overlook the diversity of bouton connection capacity (fig. S1).

We further explored whether KC subclasses demonstrate similar preferences for connections originating from distinct glomeruli. Using the same analytical methods, we found that specific connection preferences exist within several subclasses (fig. S5). Notably, the α′/β′-ap, α/β-p, α/β-s, and γ-d subclasses were identified as recipients of inputs from certain cluster 2 glomeruli, highlighting their unique odorant representations. For example, CO_2_ information is preferentially relayed by the V-glomerulus PNs to the α′/β′-ap1 subclass. Conversely, the α′/β′-m subclass primarily receives food-related information from cluster 3 glomeruli.

### Hybrid PN-to-KC networks

Next, upon further examination of the connection matrix derived from the hemibrain dataset, we identified a densely connected “L-shaped” hybrid pattern between PN clusters and KC classes (Fig. 1A and fig. S6). From the aspect of PN projection, the PN cluster 3 exhibits a relatively “divergent” projection to all three KC classes whereas PN cluster 1 exhibits a more “convergent” projection primarily targeting the γ KCs. Notably, a distinct pattern emerges when examining the matrix from the perspective of KC reception: The γ class functions as a “generalist,” receiving inputs from all PN clusters, whereas the α/β class serves as a “specialist,” receiving biased inputs only from PN cluster 3 (figs. S2 and S7, A to D).

To verify the hybrid pattern found in the hemibrain, we further analyzed the FAFB dataset (*31*). We observed that PN cluster 1 also exhibits high specificity for the γ class. Unexpectedly, PN cluster 3 shows a convergent projection connection pattern rather than a divergent one (fig. S7, E to H). To address this discrepancy, we analyzed the number of claws per KC and the total number of sampled KCs in both datasets. We found that the claw numbers for α/β and α′/β′ KCs are similar; however, γ KCs exhibit ~1.94 times more claws contacting uniglomerular PNs in the hemibrain dataset compared to the FAFB dataset (fig. S6B). Previous findings also support a higher claw count in γ KCs (*34*). Regarding neuronal composition, the number of α/β neurons sampled in the hemibrain dataset is 1.69 times that in the FAFB dataset (fig. S6C). Both the claw number and neuron number in the hemibrain dataset align with previous reports (*34*, *35*). These differences in claw counts and neuron number may contribute to the reduced divergent projection of PN cluster 3 in the FAFB dataset (*31*).

Zheng *et al.* (*22*) also demonstrated higher intracluster glomerular correlation of cluster 1 glomeruli (our classification) using conditional input analysis, examining the glomerular preferences connecting to the same KCs in the hemibrain dataset. By applying Zheng *et al.*’s approach, we reproduced the same result with our PN classification (fig. S8A). To verify the inconsistency of interglomerular correlation between the hemibrain dataset and the FAFB dataset, we further constructed a subsampled connection model of the hemibrain dataset based on the neuron number and claw count observed in the FAFB dataset (fig. S6) (*22*, *31*). We then analyzed connection preferences and performed the conditional input analysis, as proposed by Zheng *et al.* (*22*). In both the subsampled hemibrain dataset and the FAFB dataset, most of the connection preferences for γ KCs could still be observed when preserving the connection numbers of each bouton (fig. S6, D and E). On the contrary, the observed higher correlation within cluster 1 glomeruli diminishes (fig. S8B).

### Global input preferences of different KC classes

We then calculated input preferences to determine whether the input pattern of a KC class is random or structured by contrasting principal components analysis (PCA) results, specifically eigenvalues, between the observed network and networks with shuffled connections (*20*). When more KCs receive similar combinations of glomerular inputs than would be expected by random chance, the first PC (PC1) of the PN-to-KC network shows higher variance compared to a completely shuffled network. Conversely, when KCs receive connections randomly from all glomeruli, the maximum eigenvalue of the connection matrix is minimized.

As for global input preference, we performed connection shuffling within the whole calyx (see Materials and Methods), regardless of their original spatial locations. We executed separate shuffling procedures for each of the three KC classes, varying proportions from 0 to 100% (Fig. 1D and fig. S9). Our findings disclosed diverse degrees of preference across KC classes. For instance, a shuffling with ratios of 45% for γ, 45% for α′/β′, and 65% for α/β demonstrated comparable variance with fully shuffled groups (Fig. 1D and fig. S9).

Discrepancies between our findings and previous research (*20*) may stem from two factors: (i) the scale of sampling, encompassing both neuron and connection numbers, and (ii) a potential sampling bias toward specific KC classes. To ascertain the minimum number of neurons essential for detecting input preference, we conducted random subsets sampling spanning from 5 to 100% of KCs, calculating the mean variance of the PC1. Our results underscore that, to unveil the distinction between a genuine network and shuffled subnetworks, at least 100 KCs are needed for α′/β′ and γ, whereas 80 KCs suffice for α/β (fig. S10). Furthermore, detecting input preference becomes more intricate when connection data for a particular KC class remains incomplete. Even with the full collection of γ KCs, input preference was not discerned for γ when only 50% of the connections were obtained. The specific insufficiency also affects the analysis of the FAFB dataset as the number of claws in γ KCs is only about half of that in the hemibrain dataset (fig. S6). When scrutinized with the same sampling count used by Caron *et al.* (*20*), the subsampled data consistently displayed random-like traits (fig. S11). Hence, the inference of a randomly structured calyx network may find an explanation in insufficient sampling sizes.

### Biased glomerular input of multiglomerular PNs

Next, we expanded our analysis to include multiglomerular PNs (MGPNs) to further investigate how olfactory information reaches KCs. Of the 164 MGPNs in the hemibrain dataset (*32*), only 13 connect to KCs, likely excitatory based on their lineage (*36*), whereas most target other neuropils, including the LH (fig. S12, A to F). These 13 MGPNs are classified into three categories, each showing a preference for γ, α′/β′, or α/β KCs (fig. S12I). Notably, all MGPNs primarily receive odor input from cluster 1 glomeruli via both ORNs and uniglomerular PNs (fig. S12, J to M), with inputs spanning up to 23 of the 52 glomeruli. These findings suggest that MGPNs play a critical role in integrating olfactory responses from cluster 1 glomeruli.

### Spatial segregation and organization of PN-to-KC connectivity

Previous studies suggested the spatial arrangement of PNs and KCs (*17*), specifically the overlap of food-related PNs with α/β and α′/β′ classes (*22*). However, several intriguing questions remain for further investigation: (i) Is the overlap between the α/β and α′/β′ classes significant when compared to random networks? (ii) What is the spatial relationship between PN clusters and KC classes, particularly between cluster 1 PNs and the γ class? (iii) Does the spatial distribution of neurites and synapses correlate with connection preferences, supporting the hybrid connectivity pattern? (iv) If such spatial arrangements exist in CA, do they extend to other neuropils?

**Fig. 2.**
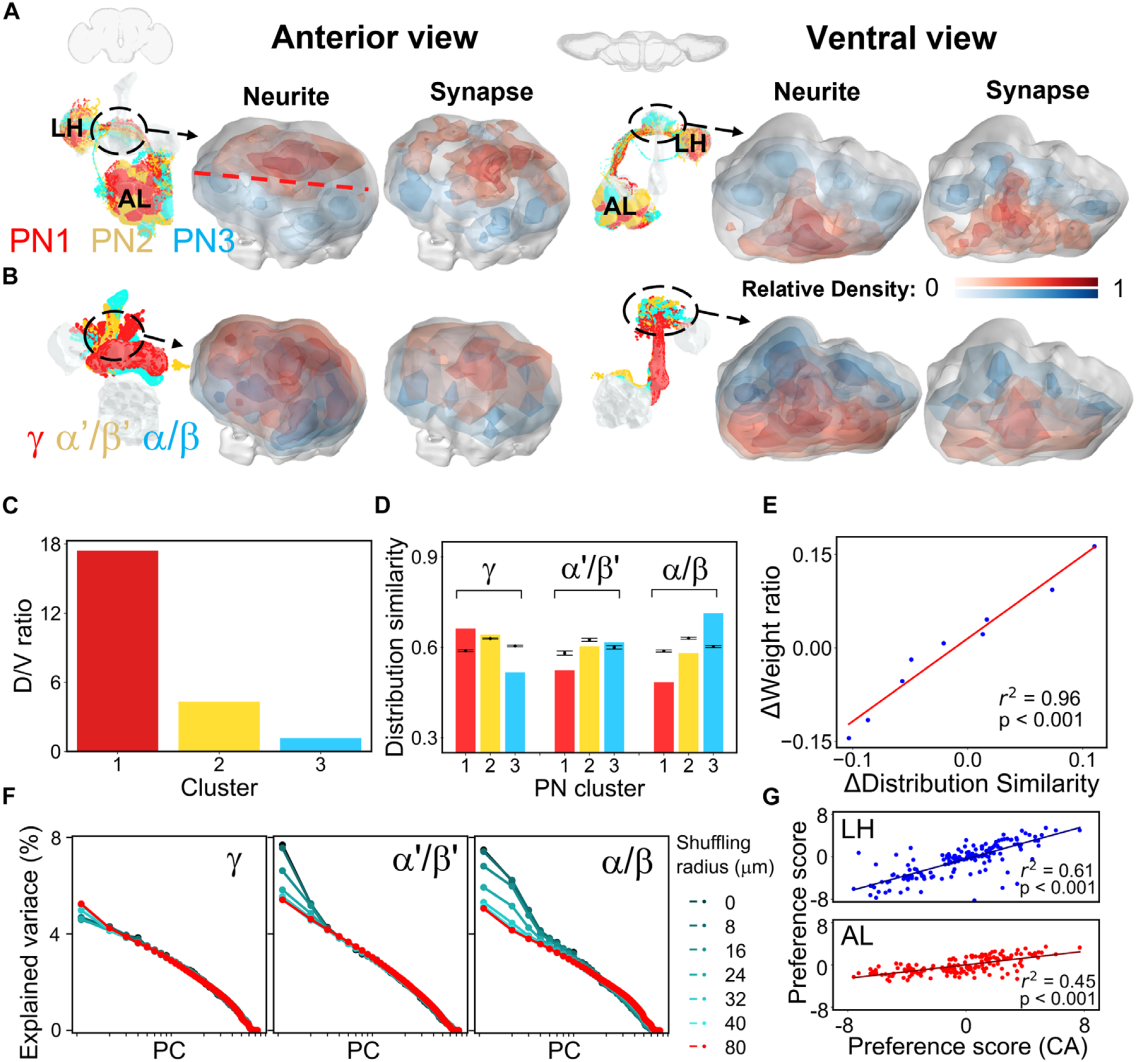
Preferential spatial connectivity between PN clusters and KC classes. (**A** and **B**) Spatial distribution of neurites and PN-to-KC synapses from each PN cluster (A) and KC class (B) shown by color code. AL, antennal lobe; LH, lateral horn; CA, calyx. (**C**) Quantitative spatial distribution of PN neurites. The dorsal/ventral (D/V) compartments are separated by the red dashed line in (A). (**D**) Distribution similarity between presynapse and postsynapse in PN-to-KC connections compared with shuffled data (*n* = 30, error bars: ± SD). (**E**) Correlation between the distribution similarity of PN-to-KC synapses and their input connection weight ratio (see Materials and Methods; fig. S2E) quantified by the difference between the real network and shuffled networks, as shown by the linear regression plot (*r*^2^ = 0.96, *P* < 0.001). (**F**) Analysis of local input preferences quantified by PCA after local random shuffling of KC claw inputs. Colors indicate different shuffling radii (refer to fig. S20 for detailed information). (**G**) Correlation of spatial innervation preference between each glomerulus and each PN cluster by the comparison between the real network and shuffled networks (see Materials and Methods), as shown by the linear regression plot (for the comparison between CA and LH, *r*^2^ = 0.61, *P* < 0.001; for the comparison between CA and AL, *r*^2^ = 0.45, *P* < 0.001).

To address these questions, we conducted a comprehensive spatial innervation analysis across three levels: neurite, synapse, and bouton/claw. Density maps of traced calycal neurites and presynapses revealed that cluster 3 PNs predominantly congregate in the ventral-posterior region, whereas cluster 1 PNs are more concentrated in the dorsal-anterior domain (Fig. 2, A and C, and fig. S13; see Materials and Methods). Similarly, dendritic arbors and postsynapses of α/β and α′/β′ KC classes are densely distributed in the ventral-posterior region, whereas the γ KC class exhibits a more uniform distribution (Fig. 2B and fig. S13).

Next, we evaluated the disparity in spatial distribution similarity between neuron collections and shuffled networks: For neurite analysis, we permuted PN and KC classifications while maintaining their total counts, and for synapse analysis, we performed categorical shuffling. Spatial innervation preferences and deviations from random placement were quantified using *z*-scores (figs. S14 and S15; see Materials and Methods). Our analysis revealed that cluster 1 and cluster 3 PNs occupy significantly distinct spatial areas compared to shuffled networks (fig. S14, A and B). Similarly, α/β and γ KC spatial distributions showed notable deviations from each other (fig. S14, C and D). When comparing PN clusters and KC classes, cluster 1 PNs preferentially project toward areas populated by γ KCs, whereas cluster 3 PNs target regions with dense dendritic arbors and postsynapses of α/β and α′/β′ KCs (Fig. 2D and figs. S14, E and F, and S15). The same results were replicated using the FAFB dataset (*31*) (fig. S16), confirming consistent spatial innervation preferences among PNs and KCs across individuals. Furthermore, detailed glomerular projection pattern analysis revealed the segregation of cluster 1 and cluster 3 PNs not only within the calyx but also in the LH (fig. S17).

We further explored the correlation between spatial innervation patterns and connection patterns by comparing observed data with shuffled networks. Our results revealed a strong correlation between spatial distribution similarity and the connection weight ratio (Fig. 2E, *r*^2^ = 0.96, *P* < 0.001; see Materials and Methods), as well as between spatial innervation preferences and connection preferences (fig. S18, *r*^2^ = 0.857, *P* < 0.001). This highlights that connection preferences can largely be attributed to spatial innervation preferences. In addition, the synaptic spatial innervation preferences between glomerular PNs and KC subclasses (fig. S19) also align closely with connection preferences (fig. S5). These findings support Peter’s rule, indicating that greater overlap between axonal and dendritic arbors increases the likelihood of synaptic connections.

### Local input preferences of KC network

The nonrandom spatial distribution of PN-to-KC connections led us to determine how spatial innervation patterns affects preference for connectivity between the PN boutons and KC claws. Considering the high energy requirements associated with neurite branching (*37*), it is plausible that KC claws exhibit a tendency to form connections primarily with adjacent PN boutons forming microglomeruli. To facilitate this exploration, we devised a local shuffling algorithm calculating the local input preferences of KCs (see Materials and Methods), allowing for localized randomization of connections within the calyx. This algorithm permits claws of a KC class to interchange their upstream boutons, solely if the relative distance is shorter than a certain threshold *R* (fig. S20), preserving the bouton connection capacity. Within a 40-μm radius, PC1 (reflecting connectivity variance) witnesses a 30% decrease for α′/β′ and a 50% decrease for α/β, respectively (Fig. 2F and fig. S20). In contrast, the PC strengths of γ presented a more uniform distribution (Fig. 2F). These outcomes underscore that the local distribution of claws is intimately linked to the preference of α′/β′ and α/β for receiving food signals, whereas this pattern is less prominent for γ, suggesting its role as a general input receiver (figs. S2 and S7). Our extensive investigation has unveiled a panorama of spatial connectivity preferences, spanning from the neurite to the synapse level.

### Segregation of PN terminals across AL, calyx, and LH

To further explore whether the spatial connectivity preferences we observed within the calyx extended to two other related neuropils, we generated density maps of the three PN clusters in the AL and the LH based on their total postsynaptic and presynaptic coordinates, respectively. Notably, our analysis of the postsynaptic density map revealed that cluster 3 PNs predominantly occupy the middle glomeruli, whereas cluster 1 PNs exhibit a distribution across the lateral glomeruli (Fig. 2A and fig. S21, A and B). Within the LH, the presynaptic density maps for cluster 3 PNs coalesce at the center, whereas cluster 1 PNs display a bilateral distribution pattern (Fig. 2A and fig. S21, A and B).

When comparing the spatial innervation preferences at the synaptic level, we observed that cluster 1 and cluster 3 PNs exhibit distinct spatial distributions from each other in both the LH and AL (figs. S17 and S21, C to F). An interesting observation emerged when we compared the preference scores for each glomerulus of PNs between cluster 1 and cluster 3—an anticorrelation is evident in both the AL and LH, consistent with our observations in the calyx (fig. S21, E to G). This pattern of spatial connectivity preferences appears to be a consistent and intriguing feature of neural connectivity across these related neuropils. A closer examination of the relationship between a glomerulus’ preferences for a specific PN cluster in both the calyx and LH revealed strong and consistent correlations (Fig. 2G and fig. S21H). These patterns are mirrored when comparing the calyx to the AL, suggesting a general phenomenon (Fig. 2G and fig. S21H). For example, when comparing the spatial innervation preference between DC3 PNs and all cluster 1 PNs, the preference is high across all the neuropils.

Furthermore, it is worth noting that cluster 3 PNs not only display this connectivity pattern but are also found to receive inputs from the same type of sensilla (fig. S22) (*38*). This remarkable uniformity across various olfactory centers underscores the presence of an inherited wiring pattern within the calyx connectome. These findings hold the potential to provide valuable insights into the fundamental mechanisms governing olfactory encoding.

### Functional correlates of calycal connection preferences

**Fig. 3.**
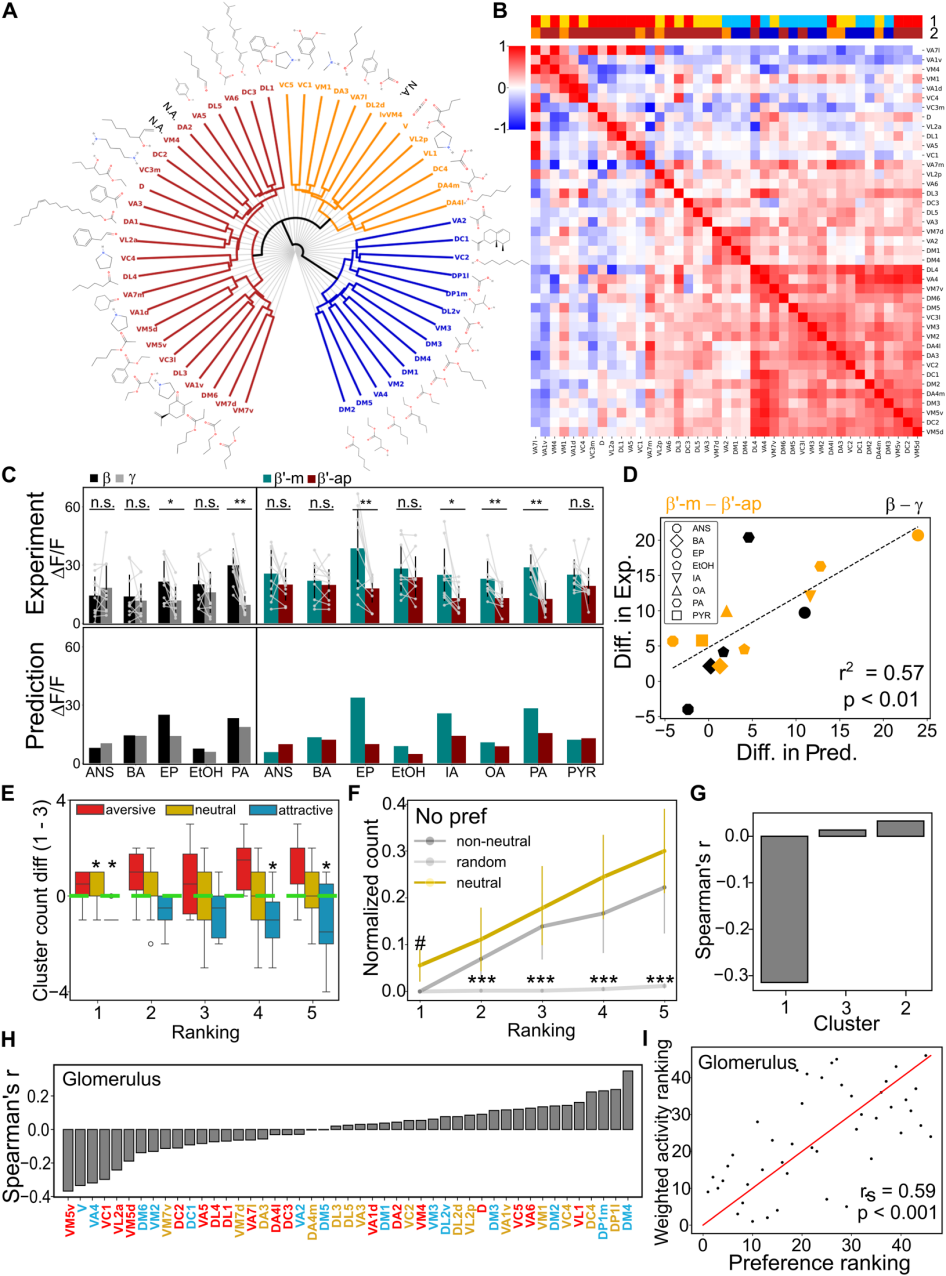
Preferential functional connectivity between PNs and KCs. (**A**) Most sensitive odorant per glomerulus, classified into three clusters (colors), based on output correlations [fig. S22, DoOR database (*43*)]. N.A., not applicable. (**B**) Correlation matrix of ORN odorant responses. Top rows show three preference clusters (Fig. 1C) and glomerular output clusters (A). (**C**) Experimental validation of predicted calcium responses (Δ*F*/*F*) for eight odorants at MB lobes (top: raw data, gray). Predicted responses used the DoOR database (*43*) and hemibrain dataset (*32*) (Wilcoxon signed-rank test: *P < 0.05 and **P < 0.01; error bars: mean + SD, *n* = 10). n.s., not significant. (**D**) Correlation between simulated and experimental calcium responses. Regression plot shows response differences between β versus γ lobes (black) and β′-m versus β′-ap (orange) in (C) (*r*^2^ = 0.57, *P* < 0.01). Markers represent different odor types. (**E**) Count difference of glomeruli between clusters 1 and 3 across top-activated rankings of odors with valences (*45*) (Kruskal-Wallis test with Bonferroni correction: *P < 0.05). (**F**) Normalized no preference glomeruli counts among top-activated rankings for neutral versus non-neutral (attractive + aversive) odors and random models (*n* = 100 for random models; for neutral and non-neutral odor lists, see Materials and Methods; Kruskal-Wallis test with Bonferroni correction: #*P* < 0.05 for neutral odors compared with others; ***P < 0.001 for random models compared with others). (**G** and **H**) Correlation of behavioral preferences with glomerular activation by cluster (G) and individual glomeruli (H) (Spearman’s correlation). (**I**) Weighted summed glomerular activity ranks correlate with behavioral preferences (46 odors; Spearman’s test, *r*_s_ = 0.59, *P* < 0.001).

To assess the functional implications of PN connection preferences within diverse olfactory information centers, we embarked on an inquiry to determine whether AL glomeruli within the same PN clusters display similar input responses to odors bearing comparable chemical attributes and similar output patterns to KCs for further processing. From the output pattern to KC perspective, our results suggested higher intracluster glomerular correlations among the three PN clusters (figs. S8C and S22). From the odor input perspective, each glomerulus receives input from distinct types of olfactory receptor neurons (ORNs), thereby inheriting specific response profiles (*5*, *39*). Before being relayed to other neuropils, these odor inputs are further processed by AL local neurons (*40*). Previous studies have revealed the clustered responsiveness of ORNs to aliphatic or aromatic (arom) odorants (*3*, *41*, *42*). In our investigation, we scrutinized ORN responses to pure odor stimuli sourced from the DoOR database (*43*). This enabled us to pinpoint the most favored ligand candidate for each ORN, indicative of the odor eliciting the most robust response. Unexpectedly, our observations unveiled an augmented sensitivity of cluster 3 glomeruli to short-chain esters featuring one- to two-carbon chains, hinting at an unexpected interplay between functionality and convergent glomerular architecture (Fig. 3A). In contrast, the most favored ligand candidates for glomeruli in clusters 1 and 2 do not exhibit clear chemical resemblances. Instead, clusters 1 and 2 manifest broad responsiveness to alcohols, aromatics, esters, and aliphatics. These intriguing findings suggest that the PN-to-KC network’s connection preferences exhibit a pronounced association with the processing of olfactory information tied to specific functional groups, rather than encompassing a universal sensitivity to all foraging scenarios.

To further investigate the correlation in responsiveness between glomeruli, we delved into the composition of the top 10 most responsive molecules of ORNs for each glomerulus. The outcomes indicated a heightened prevalence of esters within cluster 3 (fig. S23). Furthermore, we undertook a correlation analysis to quantify the likeness in ORN tuning for ~700 monomolecular odors. We identified a group of glomeruli, primarily from cluster 3 rather than other clusters, that exhibit notably high responsiveness similarity (Fig. 3B). Notably, rearranging the connection matrix based on functional correlations still captures the L-shaped preference connections (fig. S24).

### Validating the prediction of odor responses from connectivity

Next, we combined functional data from the DoOR database (*43*) with connectivity insights from the hemibrain dataset (*32*) to validate our predictions regarding how different KC classes respond to specific odors. Simulations revealed distinct odor preferences among KC classes, with short-chain esters showing a preference for α/β and α′/β′-m classes (fig. S25). To confirm our model’s accuracy, we conducted calcium imaging experiments with eight odors, targeting specific MB lobe regions corresponding to different KC classes (fig. S26, A and B). The observed calcium responses closely aligned with our predictions, demonstrating consistent functional differences among KC classes (Fig. 3C) and across individual flies (fig. S26, C and D). In addition, in the γ lobe, different subregions exhibited consistent responses to most tested odors (fig. S26, E and F; see Discussion). Furthermore, predictions based on the FAFB dataset were highly consistent with those from the hemibrain dataset, whereas shuffled networks failed to replicate the observed functional disparities, highlighting the predictive strength of our integrated model (fig. S27). In addition, we observed that these response differences between β and γ classes remained consistent across varying odor concentrations, ranging from 10^−6^ to 10^−2^ dilutions (fig. S28). This aligns with dose-dependent functional responses observed in ORNs (*41*) and PNs (*44*), as well as the spatial KC responses in the calyx (*25*) and memory-guided behaviors (*13*). The strong correlation between our experimental and simulation data was evident (*r*^2^ = 0.57, as shown in Fig. 3D). These findings collectively underscore the critical role of preferential connectivity between PNs and KCs in shaping sensory representation and conferring chemical sensitivity at the population level.

### Correlation between behavioral preferences and PN clusters/KC classes

We further explored the relationship between behavioral preferences, glomerular activation patterns, and PN-to-KC connectivity. Cluster 3 glomeruli, associated with food-related odors, and cluster 1 glomeruli, responsive to ring-structured odor molecules, appear to be linked to innate approach or avoidance behaviors. To investigate the correlation between valence and PN clusters, we analyzed differences in glomerular counts between cluster 1 and cluster 3 across different rankings. Our analysis revealed that aversive odors predominantly activate cluster 1 glomeruli, whereas attractive odors favor cluster 3 glomeruli. For neutral-valenced odors, we hypothesized that they either balance activation across aversive and attractive glomeruli or preferentially activate glomeruli with no wiring preferences. The results supported both hypotheses: Neutral odors activated glomeruli more evenly between cluster 1 and cluster 3 (Fig. 3E), and their top responsive glomerulus exhibited no wiring preferences (Fig. 3F). Both neutral and non-neutral odors showed significant differences from random models.

Using functional response data for 46 odors [selected based on behavioral experiments (*45*)] from the DoOR database, Spearman’s correlation analysis revealed a strong negative correlation between behavioral preferences and cluster 1 glomeruli (Fig. 3G). Total activity across the three PN clusters, weighted by correlation coefficients, showed a significant correlation with behavioral odor preferences (fig. S29A, *r*_s_ = 0.34, *P* < 0.05). Individual glomeruli within clusters displayed differing valences, with some cluster 1 glomeruli positively correlating with behavioral preferences (Fig. 3H). Summing weighted glomerular activity further strengthened the correlation with preferences (Fig. 3I, *r*_s_ = 0.59, *P* < 0.001). These behavioral preferences extend to KCs (fig. S29, B to E). Among KC subclasses, α/β-m and α′/β′-m showed positive correlations, whereas α/β-s and γ-m exhibited negative correlations (fig. S29D). Total weighted activity among KC subclasses also demonstrated a significant correlation with behavioral preferences (fig. S29E, *r*_s_ = 0.37, *P* < 0.01). Together, our analysis suggests that behavioral odor preferences can be predicted by integrating an ORN-PN-KC diagram derived from connectomic and functional data.

### Hybrid network architecture diversifies olfactory coding strategies

**Fig. 4.**
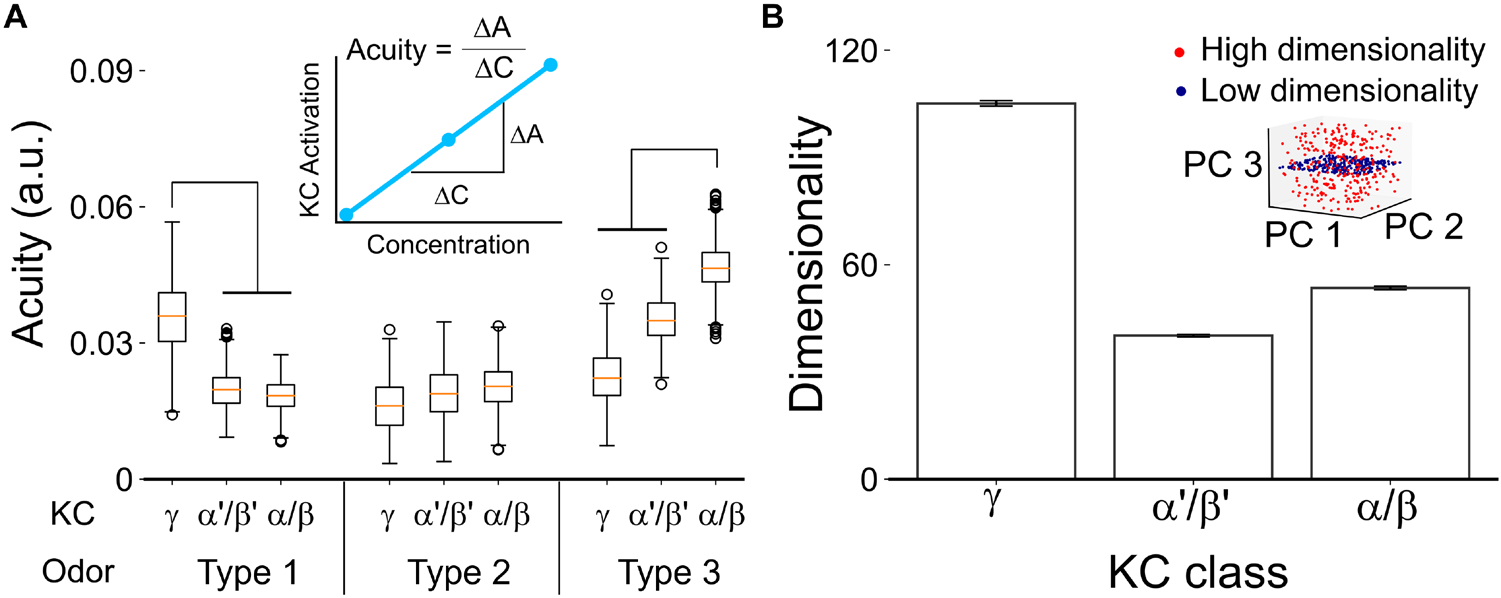
Hybrid network architecture diversifies olfactory coding strategies. (**A**) Olfactory acuity among KC classes to artificially generated odors in three odor types by numerical simulation (see Materials and Methods; Friedman test with Dunn’s post hoc test for pairwise group comparisons; for each comparison, the *P* value was < 0.001). The inset illustrates the calculation of odor acuity, which is the change in KC activation ratio over different odor concentrations. a.u., arbitrary units. (**B**) The coding capacity among KC classes for artificially generated odors is defined by dimensionality (see Materials and Methods; one-way ANOVA with Tukey’s post hoc test for pairwise group comparisons; for each comparison, the *P* value was < 0.001). The inset shows a simplified depiction of coding capacity measured by dimensionality.

The validation of predictive odor responses strongly supports the concept of a hybrid network configuration, characterized by distinct synaptic preferences and input divergence among KC classes. Aversive odors appear to activate more γ KCs via cluster 1 PNs (Fig. 3E), which exhibit higher connectivity selectivity (fig. S7A) and form the L-shaped configuration shown in Fig. 1A. This suggests that γ KCs may have enhanced acuity for odors (type 1) that exclusively activate cluster 1 glomeruli. To test this, we designed artificial odors by selectively activating specific sets of glomeruli (seven glomeruli) within each PN cluster, guided by preference scores. Connection weights were incorporated into our simulations and normalized based on the observed input weights for each KC relative to the total PN input (*9*, *46*). Simulations revealed that α/β KCs excel in detecting type 3 odors, which activates cluster 3 glomeruli, whereas γ KCs are more sensitive to type 1 odors (Fig. 4A). In addition, the divergent synaptic contacts of cluster 3 PNs and the convergent projections of cluster 1 PNs are integrated in γ KCs, positioning them as general input receivers. In natural environments, flies encounter diverse odors and odor blends, highlighting the importance of coding capacity for odor differentiation. To evaluate this, we crafted odors by randomly selecting glomeruli across clusters and examined KC Hamming code dimensionality, using binary representations for activated and inactivated states (*47*–*49*). PCA revealed that γ KCs have significantly higher coding capacity compared to α/β and α′/β′ KCs (Fig. 4B). In summary, the L-shaped hybrid network architecture provides a range of advantages: α/β KCs are highly sensitive to type 3 odors, whereas γ KCs excel in detecting type 1 odors. Moreover, this architecture maintains high coding capacity of γ KCs, enabling refined odor discrimination in complex environments.

## DISCUSSION

The MB serves as a pivotal hub in processing a vast array of olfactory cues, with specific emphasis on critical food and reproductive scents. Our detailed analysis of the connections between PNs and KCs within the MB’s calyx has illuminated an intricate hybrid architectural design that seamlessly accommodates these functional roles. Within this L-shaped hybrid architecture, cluster 1 PNs, including those sensing pheromones, converge primarily on γ KCs, whereas cluster 3 PNs, associated with food-related odors, project to all KC classes in a divergent manner. From the perspective of the KCs, α/β KCs act as specialists, primarily processing food odors, whereas γ KCs function as generalists, capable of processing a broader range of odors, including those related to food. This precise wiring specificity emerges from the spatial convergence of PN boutons and the arrangement of KC claws, strategically positioned within specific regions of the calyx as suggested previously (*17*, *18*). It is worth highlighting that this distinct connectivity motif observed between cluster 1 and cluster 3 PNs is consistently maintained across the AL, calyx, and LH. The informative simulations, driven by these established connections, provided valuable insights into the diverse responses of KCs to various odors. These simulations notably emphasized the shared sensitivities exhibited by cluster 3 PNs. Beyond this, the simulations underscored two discernible strategies: γ KCs adopt a more stochastic wiring pattern, promoting the capacity of odor coding, whereas α/β KCs leverage a stereotypical architecture to enhance detection sensitivity, particularly attuned to food odors. Furthermore, our investigation also revealed an intermediate profile in α′/β′ KCs, with α′/β′-m resembling α/β KCs, favoring short-chain esters, whereas α′/β′-ap seems displaying a preference for aroms. The correlation analysis between glomerular activation patterns and KC activity reveals that aversive odors preferentially activate more cluster 1 glomeruli, which are then transferred to γ-m and α/β-s KCs. In contrast, attractive odors activate more cluster 3 glomeruli, which are transferred to α/β-m and α′/β′-m KCs, suggesting potential valence associations with both PNs and KCs. In essence, our study provides a remarkable glimpse into the intricate hybrid wiring strategy harnessed by the *Drosophila* MB. This strategy effectively marries acuity and capacity, enabling the MB to adeptly process a diverse array of olfactory cues, including those pivotal for survival and reproduction.

Our findings on the hybrid network structure depend on the completeness of the hemibrain connectome data. Disparities in data size, even when using similar statistical approaches, can lead to different conclusions: random-like PN-to-KC patterns (*20*), a tightly correlated core community composed of food-related PNs converging on α′/β′ and α/β (*22*) and hybrid networks (current study). By downsampling according to the data size that previously used (*20*, *22*), we have reproduced the main results. Specifically, when we simulated the early dye-filling study by downsampling the data size to only 200 neurons across three KC classes with partial connections, we reproduced the random-like configuration reported by Caron *et al.* (*20*). We found that at least 100 KCs for a single KC class with more complete connection data are necessary to reveal the broader global input preferences of any particular KC class.

In addition, our results indicated a higher interglomerulus input correlation for cluster 1 (nonfood) glomeruli, mediated by uniglomerular PNs, compared to cluster 3 (food-related) glomeruli in the hemibrain dataset. By downsampling the hemibrain data to match the neural composition of the FAFB dataset, which includes fewer α/β neurons and only half the number of γ KC claws receiving input from uniglomerular PNs, we reproduced the diminished interglomerulus correlation for cluster 1 glomeruli as observed by Zheng *et al.* (*22*). Moreover, the MGPNs, which integrate cross-glomerular information, also preferentially receive input from cluster 1 glomeruli. Together, these findings suggest a more intricate interaction network involving cluster 1 glomeruli.

Our results further demonstrated that the hybrid network configuration stems from the spatial innervation patterns of PNs and KCs. PN cluster 1 (nonfood) projects densely onto the dorsal calyx, where more γ KCs innervate compared to α/β KCs. In contrast, PN cluster 3 (food-related) concentrates more on the ventral side, where both γ KCs, α′/β′, and α/β KCs are distributed. This pattern is consistently supported by both the FAFB and hemibrain datasets. Moreover, these distinct innervation patterns of PN clusters extend beyond the calyx to the AL and the LH, suggesting differentiated olfactory processing of different odor classes across neuropils along the olfactory pathways. These quasiparallel pathways may interact more in the downstream networks, forming a more complex architectural picture (*36*).

Transitioning to the functional aspect, earlier research hinted at a lack of stereotyped responsive profiles within α/β-c KCs across different individual animals at the single cell level (*24*). However, functional results showed predictable tuning features of KCs in both axonal bundle regions (*11*) and soma areas (*25*). Our model prediction followed by calcium imaging validation uncovered the preferential functional connectivity at population level. Therefore, even if stereotyped tuning profiles are absent for individual KCs, it is still possible that the soma distribution (*25*) and tuning profiles of KCs, as well as those of MBONs (*11*) maintain preferences across individuals.

Moreover, although a random connection model can generate conserved clustered odor representations (*23*) and stereotyped readout (*50*) by the MBONs, the divergent tuning profiles of different KC classes to aromatic versus aliphatic odors at population level rely on, at least, class-based connection preferences. Our study not only solidifies the notion of odor response stereotypy but also serves to validate predictions pertaining to specific odors, exemplified by odors like EP and PA that induce notably stronger responses in α/β KCs compared to their γ counterparts—a trend consistently affirmed through calcium imaging across multiple adult flies, with high correlations among fly individuals. This line of evidence led us to suggest that the connection preferences discerned from the hemibrain dataset (*32*) extracted from a single fly likely extend beyond the individual and encapsulate broader patterns within the fly population. Moreover, our analysis of calcium responses across γ lobe subregions revealed strong overall correlations, confirming the robustness of our observations, with some odor-specific variations. For example, γ2 and γ5 showed minimal correlation for benzaldehyde but high correlations for most other odors.

In addition, by integrating functional data (*43*), behavioral data (*45*), and connectomic data (*32*), we evaluated the valence of each circuitry component and predicted behavioral odor preferences. Aversive odors predominantly activate cluster 1 glomeruli, with more information transferred to γ KCs. For α/β-s KCs, input is received primarily via glomeruli such as DL1, VM7v, and VM7d, all of which have negative coefficients, inheriting negative behavioral responses. Although PNs inherit tuning profiles from ORNs (*6*), some exhibit divergent activity patterns (*40*). Also, complex interglomerular interactions exist (*51*). Therefore, further investigations are required to fully understand these complex interglomerular interactions and their impact on odor processing and behavioral responses.

The preferential connectivity between PNs from cluster 1 glomeruli and γ KCs sheds light on the critical role played by these connections in shaping behaviors related to courtship (*52*, *53*). Conversely, the α/β KCs, by virtue of their convergent input, might sacrifice some resolution, yet the presence of specific connections significantly bolsters their ability to detect food-related odors. Furthermore, the clustered responses enhance the ability for odor generalization (*23*). This intricate hybrid network architecture can be construed as a more refined model, finely attuned to satisfy the fly’s complex demands in terms of sensory sensitivity and discrimination, particularly when it comes to processes like learning and memory. In essence, our findings underscore the exquisite balance between sensitivity and discrimination achieved through the hybrid network configuration, offering insights into how neural architecture can be optimized to meet distinct functional demands.

The functionality of both artificial and biological neural networks hinges upon their underlying network architectures, playing pivotal roles in tasks ranging from discrimination to generalization (*15*, *54*). Notably, recent computational endeavors have drawn intriguing parallels between the intricate circuitry of the MB and the concept of hashing—a mechanism that transforms the multidimensional chemical space into sparse codes, thereby facilitating processes like novelty detection and generalization (*49*, *55*). In the realm of neural networks, the role of randomness in shaping connectivity is critical as random networks tend to offer heightened coding dimensionality (*56*), thereby potentially optimizing the discrimination capacity of the system.

Although we focused on the three major KC classes and several subclasses, they could be further subdivided into more families that play distinct roles in olfactory learning and memory processes (*10*, *35*, *57*–*60*). In larvae, the MB consists only of γ KC, rendering our PN classification based on KC classes inapplicable. Although further categorization of PNs into different clusters is possible in larval studies, it is beyond our current analysis approach. The exact PN connectivity preferences specific to these KC families remain unexplored. The role of the anterior paired lateral (APL) neurons innervated the entire MB in suppressing KC sparse coding using γ-aminobutyric acid is not fully understood and whether this suppression follows a random pattern is uncertain (*7*, *61*). In addition, our simulations highlight the L-shaped configuration’s acuity for pure odors activating few glomeruli within one cluster at low concentrations (*5*). However, as concentrations rise or for odor blends, activation patterns grow more complex (*62*, *63*), requiring further study. Moreover, it remains unclear whether sister PNs originating from the same glomerulus contribute to the diverse sparse coding patterns of KCs, as observed in the calycal spatial map of pheromone-sensing PNs in cockroaches (*64*, *65*). Addressing these aspects would provide a more comprehensive understanding of the intricate information processing within the MB circuitry.

## MATERIALS AND METHODS

### Data sampling

This research is mainly based on the hemibrain dataset v. 1.2.1 (*32*, *66*), a fruit fly’s hemibrain connectome with image resolution down to the synaptic level. The connection between a PN and a KC was determined by at least three synapses. Because we focused on investigating the olfactory encoding mechanism of *D. melanogaster*, only 52 types of olfaction-related glomerular PNs were selected. We excluded PNs from VP-type glomeruli and KCs without receiving any connection from the uniglomerular PNs from the 52 glomeruli. Eventually, we selected 106 uniglomerular PNs and 1745 KCs on the right side of the brain from the hemibrain dataset (*32*). KCs can be categorized into three major classes (*35*)—γ, α′/β′, and α/β—or subdivided into subclasses (*32*) including γ-m, γ-super, γ-d, α′/β′-ap1, α′/β′-ap2, α′/β′-m, α/β-m, α/β-c, α/β-p, and α/β-s. Considering that the synapses of the PN-to-KC network demonstrate high plasticity and may differ among individuals (*29*), we defined the connection between a single PN and KC as the connectivity unit (neuronal level). The selected PN-to-KC connectivity is shown in Fig. 1A.

### Connection preference evaluation

The preference score $G_{k}$ for connections from PNs of a glomerulus g to a KC k is defined as

$$G_{k}=\frac{P_{\mathrm{gk}}^{\text{observed}}-\bar{P_{\mathrm{gk}}^{\text{random}}}}{\sigma_{\mathrm{gk}}^{\text{random}}},k=\gamma ,\alpha \prime /\beta \prime ,\;\text{and}\;\alpha /\beta$$ (1)

where $P_{\mathrm{gk}}^{\text{observed}}$ is the ratio (0 to 1) of the connection from a given glomerulus *g* to the KC class *k*. $P_{\mathrm{gk}}^{\text{observed}}$ is normalized such that $\sum_{g}P_{\mathrm{gk}}^{\text{observed}}=1$ for each *k*. $P_{\mathrm{gk}}^{\text{random}}\;$ measures the same quantity but for the randomized glomerulus-to-KC network. Specifically, the targeted KCs are randomly chosen for every glomerulus, but the number of targeted KCs remains the same. The process is repeated 1000 times to calculate the mean innervating ratio ($\bar{P_{\mathrm{gk}}^{\text{random}}}$) and the SD $\sigma_{\mathrm{gk}}^{\text{random}}$. $\;G_{k}$ within the interval of ±2 is considered as no significantly preferred projection from the glomerulus g to the class-k KCs.

### Global shuffling for connections

We compared the observed data [the hemibrain dataset (*32*)] with 1000 random datasets generated by the shuffling algorithm. The shuffled ratio, *x*%, is given from 0 to 100%. For the full shuffling process (*x* = 100), all glomerular PNs were reassigned with downstream KCs at random, maintaining the total connection numbers of each glomerulus and each KC. The effect of the full shuffling process was identical to the shuffling algorithm described in Caron *et al.* (*20*). For partial shuffling, only *x*% of connections in the hemibrain dataset were randomly selected and shuffled.

### Local shuffling for connections

Compared to the global shuffling, we further set spatial constraints. After identifying the bouton/claw structure by DBSCAN (see Materials and Methods), we measured the distance between every two claws and exchanged the upstream boutons if the distance of the claws were shorter than *R*. The same process was repeated for 1000 times. The total numbers of connections, boutons, and claws were preserved under local shuffling. For the schematic plot, please see fig. S20.

### Connectivity correlation quantification

We applied Pearson correlation analysis to measure the downstream connection similarity of each two glomeruli. The connection profile of a single glomerulus is regarded as a vector, which is mapped into a 1745-dimensional space (the total number of KCs). The Pearson correlation is calculated by

$$\begin{array}{c} \begin{array}{c} C_{G,g}=\sum_{k}\frac{N_{k}^{G}-\bar{N^{G}}}{\sqrt{\sum_{K}{\left(N_{K}^{G}-\bar{N^{G}}\right)}^{2}}\;}\frac{N_{k}^{g}-\bar{N^{g}}}{\sqrt{\sum_{K}{\left(N_{K}^{g}-\bar{N^{g}}\right)}^{2}}\;},\;G,g=1\sim 52 \end{array} \end{array}$$ (2)

where $N_{k}^{G/g}$ is the total connected PN number between *G*^th^/*g*^th^ glomerulus and *k*^th^ KC, and $\bar{N^{G/g}}$ is the average downstream connection number of *G*^th^/*g*^th^ glomerulus. To visualize the convergent glomerular clusters, we implemented Ward hierarchical clustering using Python’s Scikit-Learn library.

### Input preference quantification

To estimate the degree of input preference of each KC class, the correlation-based PCA was implemented by calculating the eigenvalues of the Pearson correlation matrix of the glomerulus-to-KC connection matrix using Python’s Numpy library. The set of PCs is a linear transformation of the connection matrix that generates maximum variance, and the percent variance of the first PC (PC1) is correlated with the diversity of the input combination. If more KCs receive similar glomerular input combinations than the random connectivity hypothesis suggests, the explained variance ratio of the PC1 of the glomerulus-to-KC network would be larger than that of a completely shuffled network. We independently shuffled the connections within each of the three KC classes, varying the shuffled ratio from 0 to 100%. The degree of preference is defined as the minimal shuffled ratio *x*% that satisfies

$$x=\text{min}\left(PC_{x\%}^{1}-PC_{100\%}^{1}\geq \left(\sigma_{x\%}^{1}+\sigma_{100\%}^{1}\right)/2\;|\;x\in \left[0,100\right]\right)$$ (3)

where $PC_{x\%}^{1}$ and $\sigma_{x\%}^{1}$ are the mean and SD of the explained variance of PC1 calculated from 1000 partial shuffled connectivity trials.

### Categorical shuffling for neurites, bouton/claw, and synapses

We used a categorical shuffling algorithm to explore whether different classes of KCs and clusters of PNs occupy distinct regions within the calyx, across neurite, bouton/claw, and synapse levels. For neurites, we shuffled the classification of neurons (the class of a KC or the cluster of a PN) while maintaining the total number of neurons in each category. For example, when examining KC classes, we randomly reassigned the KC class for each KC while keeping the overall number of neurons in each class constant. For the bouton/claw aspect, we shuffled the identity of each claw/bouton while preserving the total number of claws/boutons associated with each neuron. Similarly, for synapses, we shuffled the identity of each synapse while maintaining the total number of synaptic sites associated with each neuron. In particular, we focused on the hypothetical scenario where neuron *A* establishes connections with neuron *B* at only one specific site. To account for this, we treated all synapses between neurons *A* and *B* as a single unit during the permutation process, ensuring that the overall number of synaptic sites remained consistent while introducing random shuffling.

### Odor selection for functional response

We collected the response profiles of 52 olfaction-related olfactory receptors (ORs) from the DoOR database (*43*). Each glomerulus connects with a distinct type of ORNs, and most ORNs only express a single type of OR (*67*). The DoOR dataset includes the OR’s response to 693 odors, which is normalized by the following equation

$$A=\left(F_{\text{activated}}-F_{\text{resting}}\right)/F_{\text{resting}}$$ (4)

where *A* is the response activity. *F_resting_* is derived from the response to mineral oil. *F_activated_* is derived from the response to other odors. The missing values in DoOR are manually converted to “0” in our study.

### Simulation for odor response in MB lobe

The odor response of the MB lobe is closely correlated with the activity of its upstream glomeruli and the strength of their connections. Each KC class has a distinct average claw number, and KCs with a higher claw number demonstrate a higher activation threshold (*9*, *46*). As a result, the KC response level has a higher correlation with the percentage of claws than the absolute number of claws that receive inputs. Therefore, we assumed that the KC response level is associated with the activated ratio of KCs, denoted as $R_{\mathrm{gk}}$ and given by

$$R_{\mathrm{gk}}=\frac{W_{\mathrm{gk}}}{\sum_{k}W_{\mathrm{gk}}}$$ (5)

where $W_{\mathrm{gk}}$ represents the number of connected claws between glomerulus, *g*, and a single KC, *k*. $R_{\mathrm{gk}}$ is a normalized value, which is divided by the total claw number of each KC.

The simulated odor response of the MB lobe, represented as $\frac{\Delta F}{F}$, is determined by the summation of inputs

$$\frac{\Delta F}{F}=\frac{\sum_{k}C_{g}R_{\mathrm{gk}}}{N}$$ (6)

where *N* represents the total number of KCs in a specific class, and $C_{g}$ is the activity of the upstream glomerulus, *g*. On the basis of the one-to-one mapping structure, we assumed that the glomerular activity is proportional to the activity of its upstream ORNs, and the level of ORN odor-evoked activity refers to the data from the DoOR database (*43*).

### Spatial distribution similarity analysis

To compute distribution similarity, the first step was to collect all the skeleton points, center points of boutons/claws, and synapse coordinates according to their KC classes or PN clusters. Then, we attained the spatial distribution by kernel density estimation, using Python’s SciPy library. Next, we calculated the Jensen-Shannon divergence (JSD) to estimate the distribution similarity using the SciPy package as well (*68*). JSD’s advantages include (i) boundedness and (ii) symmetry. Therefore, it helped us normalize the similarity among different distributions. JSD is defined by

$$\mathrm{JSD}\left(P\|Q\right)=\frac{1}{2}\sum p\left(x\right)\text{log}\left[\frac{p\left(x\right)}{\frac{p\left(x\right)+q\left(x\right)}{2}}\right]+\frac{1}{2}\sum q\left(x\right)\text{log}\left[\frac{q\left(x\right)}{\frac{p\left(x\right)+q\left(x\right)}{2}}\right]$$ (7)

S=1−JSD (8)

where *P* and *Q* are the distribution of *p* and *q*. *S* is the distribution similarity. The spatial distribution preference is evaluated by the difference in distribution similarity between the original data and the shuffled ones.

### Bouton/claw identification

To identify the middle-scale anatomic structures—boutons for a PN and claws for a KC—we used DBSCAN from Python’s SciPy library. The minimum number of points was set to 3, which is consistent with our connection threshold, and the radius was set to 1.6 μm because one bouton connecting to several KC claws should not be identified as several boutons. After manual inspection, we established a distance threshold of 2.8 μm. If the distance between the centers of two boutons/claws from a PN/KC was less than 2.8 μm, these two structures merged into a single entity automatically. The example results are presented in fig. S1B.

### Weight ratio calculation

The input weight ratio was calculated based on the connection weight from a PN cluster to a KC, divided by the total connection weight for the KC from all PNs. Specifically, the input weight ratio is given by the formula

$$R_{\mathrm{PN},i}=\frac{\sum_{n=1}^{N_{i}}w_{i,n}}{\sum_{i}^{C}\sum_{n=1}^{N_{i}}w_{i,n}}$$ (9)

where *R_PN,i_* is the input weight ratio of a KC class receiving from a cluster *i*, *N_i_* is the total number of PNs in cluster *i*, *C* is the number of clusters, and *w_i,n_* is the connection weight of a PN to a KC.

As for the output weight ratio, it was calculated using the connection weight for a PN to a KC class, divided by the total connection weight for a PN to all KC classes. The value is defined as follows

$$R_{\mathrm{KC},j}=\frac{\sum_{n=1}^{N_{j}}w_{j,n}}{\sum_{j}^{K}\sum_{n=1}^{N_{j}}w_{j,n}}$$ (10)

where *R_KC,j_* is the output weight ratio of a PN cluster to a KC class *j*, *N_j_* is the total number of KCs in KC class *j*, *K* is the number of KC classes, and *w_j,n_* is the connection weight of a PN to a KC.

### Correlation of weight ratio and synaptic distribution preference

To evaluate the correlation between the connection weight ratio and the spatial distribution, we calculated the differences in the connection weight ratio (for each KC class to each PN cluster) between the original network and the shuffled connection ratio. Next, we calculated the differences in the spatial distribution preference by subtracting the shuffled distribution similarity from the original distribution similarity. Last, the correlation between the difference in the connection weight ratio and the difference in distribution similarity was estimated by performing linear regression using Python’s SciPy package.

### Weight normalization for generated odor simulation

Previous studies showed that the KC activation is correlated with the percentage of the claws that receive inputs (*9*, *46*). Therefore, we normalized the connection matrix *W_PN>KC_* by the column. As a result, each connection weight between a PN to a KC is given by

$$w_{{\mathrm{PN}}_{i}>{\mathrm{KC}}_{j}}\prime =\frac{w_{{\mathrm{PN}}_{i}>{\mathrm{KC}}_{j}}}{\sum_{i=1}^{n}w_{{\mathrm{PN}}_{i}>{\mathrm{KC}}_{j}}}$$ (11)

where *n* is the total number of PN and *w_PNi>KCj_* represents the original connection weight (synapse number) of PN *i* to KC *j*.

### Simulation for artificial odors

To investigate the impact of connection preferences on KC coding, we generated artificial odors that selectively activate glomeruli within the same cluster for acuity experiments and across multiple clusters for coding capacity experiments. Here, we randomly chose *m* candidate glomeruli from *n* glomeruli and defined the activity *A_PN_* of the PNs from the chosen glomeruli as

$$\;A_{\mathrm{PNi}}=C_{\text{odor}}$$ (12)

otherwise

$$A_{\mathrm{PNi}}=0$$ (13)

where *C_odor_* is set from 7 to 11 to observe how odor stimulation strength affects KC activation. The unit of *C_odor_* is arbitrary. Note that the absolute value of *C_odor_* is not important as it can always be compensated by the scaling factor and activation threshold introduced below. The range of *C_odor_* is used to simulate the firing rate dependency of PNs to the odor concentration. For odors that only activate cluster 1 glomeruli, we defined the odors as type 1 odors. For each odor type, we generated 1000 different odors removing the same activated glomerular combination.

### Simulation for KC representation of artificial odors

To investigate how the connection preference affects KC coding, we used the artificially generated odors with the observed connection matrix derived from the hemibrain dataset (*32*) to analyze the KC activation profile. The activity of KC is given by

$$A_{\mathrm{KC}}=\text{Max}\left(0,s_{\text{scaling}}A_{\mathrm{PN}}\;W_{\mathrm{PN}>\mathrm{KC}}-\theta \right)$$ (14)

where θ represents the activity threshold, which was set to 1. *A_KC_* is the vector representing KC activity for each odor filtered through the ReLU function. *A_PN_* is derived from the artificial odors mentioned above. *s_scaling_* is a scaling factor that was set to 0.3. *W_PN>KC_* corresponds to the connection matrix. Here, we carefully adjusted *s_scaling_* and θ to achieve a KC activation ratio of ~10% when the *C_odor_* is 8. This ratio is in line with a previous experimental study (*44*).

### Odor selection for behavioral analysis

We analyzed top aversive odors (1-octen-3-ol, acetophenone, linalool, 2-methylphenol, benzaldehyde, and 1-octanol), top attractive odors (γ-butyrolactone, 2,3-butanedione, hexanoic acid, pentanoic acid, 3-methylthio-1-propanol, and 4-ethylguaiacol), and the most neutral odors (ethyl hexanoate, β-citronellol, ammonium hydroxide, pentanal, ethanol, acetaldehyde, nerol, linoleic acid, *E*3-hexenol, 2-propenal, *E*2-hexenol, terpinolene, nonanoic acid, *E*2-hexenal, and γ-octalactone) (*45*). For each odor type, we calculated the counts of glomeruli in each cluster and the no wiring preference group across the top 1 to 5 rankings.

### Correlation analysis for behavioral odor preference

For a total of 46 odors (*45*) [excluding six odors lacking sufficient functional data from the DoOR database (*43*)], we obtained tuning profiles from the DoOR database after subtracting the baseline response to oil. We then calculated the Spearman’s correlation for each cluster and each glomerulus with the behavioral preference ranking (*45*). Using the correlation coefficients as weighting parameters, we computed the summed activity by applying these weights to the tuning profiles. The summed activity is given by

$$A_{\text{total}}=\sum A_{C}r_{s}$$

where 𝐴 represents the activity, 𝑐 denotes different clusters or glomeruli, and 𝑟_s_ is the Spearman’s correlation coefficient.

For KC activation, we multiplied the activity by the connection matrix and applied a threshold to activate only the top 10% of KCs. We then calculated the Spearman’s correlation coefficient. For the summed weighted activity, the same procedures were applied as for PN clusters and glomeruli.

### Acuity analysis

To evaluate olfactory acuity for different odor classes, we first assessed the activation ratio of KCs. The activation ratio was calculated by dividing the number of activated KCs by the total number of KCs in response to each odor stimulus.

Next, we analyzed the relationship between the odor stimulation strength (*C_odor_*) and the activation ratio by performing linear regression using Python’s SciPy library. This regression analysis allows us to estimate the slope, which represents the change in the activation ratio per unit change in odor stimulation strength (*C_odor_*). The odor acuity for a KC class to an odor is defined by the slope of the regression line.

### Dimensionality analysis

To compute the dimensionality of KC representation, we performed PCA decomposition for the KC response matrix. The dimensionality is defined by (*56*)

$$\text{dim}\;\left(A_{\mathrm{KC}}\right)=\frac{{\left(\sum \lambda \right)}^{2}}{\sum \lambda^{2}}$$ (15)

where λ is the eigenvalue. For hamming code capacity calculation, all positive *A_KC_* values were set to 1.

### Fly strains

All fly stocks were reared on standard cornmeal-yeast-agar medium at 25° or 18°C with around 70% humidity under a 12-hour light/12-hour dark cycle. The following fly lines were used in the current study: *OK107-Gal4* drives the expression of all three classes of MB neurons, *VT57244-Gal4* (v200970, Vienna Drosophila Resource Center) for the α′/β′ neurons, and *UAS-GCaMP7f* (79031, Bloomington Drosophila Stock Center) flies carrying a transgene for a genetically encoded calcium sensor.

### Chemicals and stimulation

For olfactory stimulation, ethyl propionate (EP; CAS: 105-37-3, Sigma-Aldrich), isopentyl acetate (IA; CAS: 123-92-2, Sigma-Aldrich), octyl acetate (OA; CAS: 112-14-1, Sigma-Aldrich), pentyl acetate (PA; CAS: 628-63-7, Sigma-Aldrich), benzaldehyde (BA; CAS: 100-52-7, Scharlau), anisole (ANS; CAS: 100-66-3, Scharlau), pyrrolidine (PYR; CAS: 123-75-1, Sigma-Aldrich), ethanol (EtOH; CAS: 64-17-5, Honeywell), and 11-*cis*-vaccenyl acetate (cVA; CAS: 6186-98-7, Cayman Chemical) were used. Odors were diluted in mineral oil. For cVA, the concentration was 10^−3^ (v/v), whereas for the other odors, the concentration was 10^−6^ (v/v) in the single concentration test. Concentration gradient experiments were adjusted from 10^−6^ to 10^−2^ (v/v). The diluted solution was packed in glass tubes at a volume of 10 ml per tube. Using a computer to control the olfactometer, the odor was introduced into a continuous flow of air for the antennae to detect. Stimulus duration was 10 s. Airstream continued for the entire experiment time.

### Confocal imaging and imaging processing

The sample brains were imaged by a Zeiss LSM 710 or LSM 780 confocal microscope with a 40× C-Apochromat water immersion lens. Images were scanned under the following setting: resolution of 512 × 512 pixels, scanning speed of 7, and line average of 2 in ZEN software (Zeiss). Max-projection images and single-plane images were formatted using ZEN software (Zeiss).

### In vivo GCaMP functional imaging and imaging processing

Flies expressing *UAS-GCaMP6* in α′/β′ KC and *UAS-GCaMP7* in whole KC were mounted on a droplet-shaped sheet, with a window opened on the head capsule, and then adult hemolymph-like (AHL) saline [108 mM NaCl, 5 mM KCl, 2 mM CaCl_2_, 8.2 mM MgCl_2_, 4 mM NaHCO_3_, 1 mM NaH_2_PO_4_, 5 mM trehalose, 10 mM sucrose and 5 mM Hepes (pH 7.5), and 265 mosmol/kg H_2_O) added immediately.

Recording of changes in GCaMP intensity before and after odor stimulations was performed on Zeiss LSM 780 with a 40× C-Apochromat water immersion lens. Images were acquired at a resolution of 512 × 512 pixels, captured at 2 frames/s for a total of 60 frames. For odor stimulation, odorants were delivered during 10 to 20 s for odor stimulation in each 30-s trial. Each odor was presented with an interstimulus interval of 1 min.

A 488-nm light-emitting diode light source was placed beneath the chamber to provide red-light stimulations. MB lobes were imaged in an area of the dorsal surface of the fly brain (fig. S26). The regions of interest of α/β KC and γ KC were circled at the tip of the lobe, whereas α′/β′-m KC and α′/β′-ap KC were circled according to the position of the nerve distribution provided by neuPrint (*66*).

### Data analysis for functional imaging

The raw fluorescence signals were converted to Δ*F*/*F*, where *F* is the averaged baseline fluorescence value of 10 s before the stimulation onset, and Δ*F* is the difference between the highest signal and the mean. Peaks of postresponse were taken as maximum Δ*F*/*F* within the 10 s following light onset, compared using the nonparametric Wilcoxon signed-rank test.

### Statistics

Statistical analysis was performed by Microsoft Excel, GraphPad Prism, and Python. Regression analysis was used to capture the correlation between two variables. The nonparametric Wilcoxon signed-rank test was performed to compare the calcium responses between two lobes.

For non-normal distributed group data, Friedman test with Dunn’s post hoc test was used. Otherwise, we performed one-way analysis of variance (ANOVA), with Tukey’s post hoc test. The significance thresholds are indicated by *P < 0.05, **P < 0.01, and ***P < 0.001. All data are presented by mean values ± SD.

## References

[1] M. Heisenberg, Mushroom body memoir: From maps to models. Nat. Rev. Neurosci. 4, 266–275 (2003). 12671643 10.1038/nrn1074

[2] M. N. Modi, Y. Shuai, G. C. Turner, The Drosophila mushroom body: From architecture to algorithm in a learning circuit. Annu. Rev. Neurosci. 43, 465–484 (2020). 32283995 10.1146/annurev-neuro-080317-0621333

[3] E. A. Hallem, J. R. Carlson, Coding of odors by a receptor repertoire. Cell 125, 143–160 (2006). 16615896 10.1016/j.cell.2006.01.050

[4] A. F. Silbering, R. Rytz, Y. Grosjean, L. Abuin, P. Ramdya, G. S. Jefferis, R. Benton, Complementary function and integrated wiring of the evolutionarily distinct *Drosophila* olfactory subsystems. J. Neurosci. 31, 13357–13375 (2011). 21940430 10.1523/JNEUROSCI.2360-11.2011 PMC6623294

[5] J. W. Wang, A. M. Wong, J. Flores, L. B. Vosshall, R. Axel, Two-photon calcium imaging reveals an odor-evoked map of activity in the fly brain. Cell 112, 271–282 (2003). 12553914 10.1016/s0092-8674(03)00004-7

[6] C. M. Root, J. L. Semmelhack, A. M. Wong, J. Flores, J. W. Wang, Propagation of olfactory information in Drosophila. Proc. Natl. Acad. Sci. U.S.A. 104, 11826–11831 (2007). 17596338 10.1073/pnas.0704523104 PMC1913902

[7] A. C. Lin, A. M. Bygrave, A. de Calignon, T. Lee, G. Miesenbock, Sparse, decorrelated odor coding in the mushroom body enhances learned odor discrimination. Nat. Neurosci. 17, 559–568 (2014). 24561998 10.1038/nn.3660 PMC4000970

[8] A. Lüdke, G. Raiser, J. Nehrkorn, A. V. Herz, C. G. Galizia, P. Szyszka, Calcium in Kenyon cell somata as a substrate for an olfactory sensory memory in Drosophila. Front. Cell. Neurosci. 12, 128 (2018). 29867361 10.3389/fncel.2018.00128 PMC5960692

[9] H. Li, Y. Li, Z. Lei, K. Wang, A. Guo, Transformation of odor selectivity from projection neurons to single mushroom body neurons mapped with dual-color calcium imaging. Proc. Natl. Acad. Sci. U.S.A. 110, 12084–12089 (2013). 23818618 10.1073/pnas.1305857110 PMC3718165

[10] Y. Aso, D. Sitaraman, T. Ichinose, K. R. Kaun, K. Vogt, G. Belliart-Guérin, P.-Y. Plaçais, A. A. Robie, N. Yamagata, C. Schnaitmann, W. J. Rowell, R. M. Johnston, T.-T. B. Ngo, N. Chen, W. Korff, M. N. Nitabach, U. Heberlein, T. Preat, K. M. Branson, H. Tanimoto, G. M. Rubin, Mushroom body output neurons encode valence and guide memory-based action selection in *Drosophila*. Elife 3, e04580 (2014). 25535794 10.7554/eLife.04580 PMC4273436

[11] T. Hige, Y. Aso, G. M. Rubin, G. C. Turner, Plasticity-driven individualization of olfactory coding in mushroom body output neurons. Nature 526, 258–262 (2015). 26416731 10.1038/nature15396 PMC4860018

[12] J. K. Wu, C. Y. Tai, K. L. Feng, S. L. Chen, C. C. Chen, A. S. Chiang, Long-term memory requires sequential protein synthesis in three subsets of mushroom body output neurons in *Drosophila*. Sci. Rep. 7, 7112 (2017). 28769066 10.1038/s41598-017-07600-2 PMC5540930

[13] P. Masek, M. Heisenberg, Distinct memories of odor intensity and quality in *Drosophila*. Proc. Natl. Acad. Sci. U.S.A. 105, 15985–15990 (2008). 18824685 10.1073/pnas.0804086105 PMC2556361

[14] R. A. Campbell, K. S. Honegger, H. Qin, W. Li, E. Demir, G. C. Turner, Imaging a population code for odor identity in the *Drosophila* mushroom body. J. Neurosci. 33, 10568–10581 (2013). 23785169 10.1523/JNEUROSCI.0682-12.2013 PMC3685844

[15] O. Barak, M. Rigotti, S. Fusi, The sparseness of mixed selectivity neurons controls the generalization-discrimination trade-off. J. Neurosci. 33, 3844–3856 (2013). 23447596 10.1523/JNEUROSCI.2753-12.2013 PMC6119179

[16] S. Dasgupta, C. F. Stevens, S. Navlakha, A neural algorithm for a fundamental computing problem. Science 358, 793–796 (2017). 29123069 10.1126/science.aam9868

[17] H. H. Lin, J. S. Lai, A. L. Chin, Y. C. Chen, A. S. Chiang, A map of olfactory representation in the *Drosophila* mushroom body. Cell 128, 1205–1217 (2007). 17382887 10.1016/j.cell.2007.03.006

[18] G. S. X. E. Jefferis, C. J. Potter, A. M. Chan, E. C. Marin, T. Rohlfing, C. R. Maurer Jr., L. Luo, Comprehensive maps of *Drosophila* higher olfactory centers: Spatially segregated fruit and pheromone representation. Cell 128, 1187–1203 (2007). 17382886 10.1016/j.cell.2007.01.040 PMC1885945

[19] L. M. Masuda-Nakagawa, N. K. Tanaka, C. J. O’Kane, Stereotypic and random patterns of connectivity in the larval mushroom body calyx of *Drosophila*. Proc. Natl. Acad. Sci. U.S.A. 102, 19027–19032 (2005). 16357192 10.1073/pnas.0509643102 PMC1323213

[20] S. J. Caron, V. Ruta, L. F. Abbott, R. Axel, Random convergence of olfactory inputs in the *Drosophila* mushroom body. Nature 497, 113–117 (2013). 23615618 10.1038/nature12063 PMC4148081

[21] T. T. Hayashi, A. J. MacKenzie, I. Ganguly, K. E. Ellis, H. M. Smihula, M. S. Jacob, A. Litwin-Kumar, S. J. C. Caron, Mushroom body input connections form independently of sensory activity in *Drosophila* melanogaster. Curr. Biol. 32, 4000–4012.e5 (2022). 35977547 10.1016/j.cub.2022.07.055 PMC9533768

[22] Z. Zheng, F. Li, C. Fisher, I. J. Ali, N. Sharifi, S. Calle-Schuler, J. Hsu, N. Masoodpanah, L. Kmecova, T. Kazimiers, E. Perlman, M. Nichols, P. H. Li, V. Jain, D. D. Bock, Structured sampling of olfactory input by the fly mushroom body. Curr. Biol. 32, 3334–3349.e6 (2022). 35797998 10.1016/j.cub.2022.06.031 PMC9413950

[23] K. Endo, Y. Tsuchimoto, H. Kazama, Synthesis of conserved odor object representations in a random, divergent-convergent network. Neuron 108, 367–381.e5 (2020). 32814018 10.1016/j.neuron.2020.07.029

[24] M. Murthy, I. Fiete, G. Laurent, Testing odor response stereotypy in the *Drosophila* mushroom body. Neuron 59, 1009–1023 (2008). 18817738 10.1016/j.neuron.2008.07.040 PMC2654402

[25] Y. Wang, H. F. Guo, T. A. Pologruto, F. Hannan, I. Hakker, K. Svoboda, Y. Zhong, Stereotyped odor-evoked activity in the mushroom body of *Drosophila* revealed by green fluorescent protein-based Ca^2+^ imaging. J. Neurosci. 24, 6507–6514 (2004). 15269261 10.1523/JNEUROSCI.3727-03.2004 PMC6729867

[26] S. Cachero, G. S. X. E. Jefferis, *Drosophila* olfaction: The end of stereotypy? Neuron 59, 843–845 (2008). 18817725 10.1016/j.neuron.2008.09.017

[27] A. M. Mittal, D. Gupta, A. Singh, A. C. Lin, N. Gupta, Multiple network properties overcome random connectivity to enable stereotypic sensory responses. Nat. Commun. 11, 1023 (2020). 32094345 10.1038/s41467-020-14836-6 PMC7039968

[28] S. Namiki, M. Takaguchi, Y. Seki, T. Kazawa, R. Fukushima, C. Iwatsuki, R. Kanzaki, Concentric zones for pheromone components in the mushroom body calyx of the moth brain. J. Comp. Neurol. 521, 1073–1092 (2013). 22911613 10.1002/cne.23219

[29] L. Baltruschat, L. Prisco, P. Ranft, J. S. Lauritzen, A. Fiala, D. D. Bock, G. Tavosanis, Circuit reorganization in the *Drosophila* mushroom body calyx accompanies memory consolidation. Cell Rep. 34, 108871 (2021). 33730583 10.1016/j.celrep.2021.108871 PMC8515896

[30] K. Choi, W. K. Kim, C. Hyeon, Olfactory responses of *Drosophila* are encoded in the organization of projection neurons. Elife 11, e77748 (2022). 36173095 10.7554/eLife.77748 PMC9616568

[31] Z. Zheng, J. S. Lauritzen, E. Perlman, C. G. Robinson, M. Nichols, D. Milkie, O. Torrens, J. Price, C. B. Fisher, N. Sharifi, S. A. Calle-Schuler, L. Kmecova, I. J. Ali, B. Karsh, E. T. Trautman, J. A. Bogovic, P. Hanslovsky, G. S. X. E. Jefferis, M. Kazhdan, K. Khairy, S. Saalfeld, R. D. Fetter, D. D. Bock, A complete electron microscopy volume of the brain of adult *Drosophila* melanogaster. Cell 174, 730–743.e22 (2018). 30033368 10.1016/j.cell.2018.06.019 PMC6063995

[32] L. K. Scheffer, C. S. Xu, M. Januszewski, Z. Lu, S.-Y. Takemura, K. J. Hayworth, G. B. Huang, K. Shinomiya, J. Maitlin-Shepard, S. Berg, J. Clements, P. M. Hubbard, W. T. Katz, L. Umayam, T. Zhao, D. Ackerman, T. Blakely, J. Bogovic, T. Dolafi, D. Kainmueller, T. Kawase, K. A. Khairy, L. Leavitt, P. H. Li, L. Lindsey, N. Neubarth, D. J. Olbris, H. Otsuna, E. T. Trautman, M. Ito, A. S. Bates, J. Goldammer, T. Wolff, R. Svirskas, P. Schlegel, E. Neace, C. J. Knecht, C. X. Alvarado, D. A. Bailey, S. Ballinger, J. A. Borycz, B. S. Canino, N. Cheatham, M. Cook, M. Dreher, O. Duclos, B. Eubanks, K. Fairbanks, S. Finley, N. Forknall, A. Francis, G. P. Hopkins, E. M. Joyce, S. J. Kim, N. A. Kirk, J. Kovalyak, S. A. Lauchie, A. Lohff, C. Maldonado, E. A. Manley, S. M. Lin, C. Mooney, M. Ndama, O. Ogundeyi, N. Okeoma, C. Ordish, N. Padilla, C. M. Patrick, T. Paterson, E. E. Phillips, E. M. Phillips, N. Rampally, C. Ribeiro, M. K. Robertson, J. T. Rymer, S. M. Ryan, M. Sammons, A. K. Scott, A. L. Scott, A. Shinomiya, C. Smith, K. Smith, N. L. Smith, M. A. Sobeski, A. Suleiman, J. Swift, S. Takemura, I. Talebi, D. Tarnogorska, E. Tenshaw, T. Tokhi, J. J. Walsh, T. Yang, J. A. Horne, F. Li, R. Parekh, P. K. Rivlin, V. Jayaraman, M. Costa, G. S. Jefferis, K. Ito, S. Saalfeld, R. George, I. A. Meinertzhagen, G. M. Rubin, H. F. Hess, V. Jain, S. M. Plaza, A connectome and analysis of the adult *Drosophila* central brain. Elife 9, e57443 (2020). 32880371 10.7554/eLife.57443 PMC7546738

[33] K. Yang, T. Liu, Z. Wang, J. Liu, Y. Shen, X. Pan, R. Wen, H. Xie, Z. Ruan, Z. Tan, Y. Chen, A. Guo, H. Liu, H. Han, Z. Di, K. Zhang, Classifying *Drosophila* olfactory projection neuron boutons by quantitative analysis of electron microscopic reconstruction. iScience 25, 104180 (2022). 35494235 10.1016/j.isci.2022.104180 PMC9038572

[34] N. J. Butcher, A. B. Friedrich, Z. Lu, H. Tanimoto, I. A. Meinertzhagen, Different classes of input and output neurons reveal new features in microglomeruli of the adult *Drosophila* mushroom body calyx. J. Comp. Neurol. 520, 2185–2201 (2012). 22237598 10.1002/cne.23037

[35] Y. Aso, D. Hattori, Y. Yu, R. M. Johnston, N. A. Iyer, T. T. Ngo, H. Dionne, L. F. Abbott, R. Axel, H. Tanimoto, G. M. Rubin, The neuronal architecture of the mushroom body provides a logic for associative learning. Elife 3, e04577 (2014). 25535793 10.7554/eLife.04577 PMC4273437

[36] A. S. Bates, P. Schlegel, R. J. V. Roberts, N. Drummond, I. F. M. Tamimi, R. Turnbull, X. Zhao, E. C. Marin, P. D. Popovici, S. Dhawan, A. Jamasb, A. Javier, L. Serratosa Capdevila, F. Li, G. M. Rubin, S. Waddell, D. D. Bock, M. Costa, G. S. X. E. Jefferis, Complete connectomic reconstruction of olfactory projection neurons in the fly brain. Curr. Biol. 30, 3183–3199.e6 (2020). 32619485 10.1016/j.cub.2020.06.042 PMC7443706

[37] H. Cuntz, A. Mathy, M. Hausser, A scaling law derived from optimal dendritic wiring. Proc. Natl. Acad. Sci. U.S.A. 109, 11014–11018 (2012). 22715290 10.1073/pnas.1200430109 PMC3390826

[38] A. Couto, M. Alenius, B. J. Dickson, Molecular, anatomical, and functional organization of the *Drosophila* olfactory system. Curr. Biol. 15, 1535–1547 (2005). 16139208 10.1016/j.cub.2005.07.034

[39] J. G. Hildebrand, G. M. Shepherd, Mechanisms of olfactory discrimination: Converging evidence for common principles across phyla. Annu. Rev. Neurosci. 20, 595–631 (1997). 9056726 10.1146/annurev.neuro.20.1.595

[40] V. Bhandawat, S. R. Olsen, N. W. Gouwens, M. L. Schlief, R. I. Wilson, Sensory processing in the *Drosophila* antennal lobe increases reliability and separability of ensemble odor representations. Nat. Neurosci. 10, 1474–1482 (2007). 17922008 10.1038/nn1976 PMC2838615

[41] G. Si, J. K. Kanwal, Y. Hu, C. J. Tabone, J. Baron, M. Berck, G. Vignoud, A. D. T. Samuel, Structured odorant response patterns across a complete olfactory receptor neuron population. Neuron 101, 950–962.e7 (2019). 30683545 10.1016/j.neuron.2018.12.030 PMC6756926

[42] H. K. M. Dweck, S. A. M. Ebrahim, T. Retzke, V. Grabe, J. Weißflog, A. Svatos, B. S. Hansson, M. Knaden, The olfactory logic behind fruit odor preferences in larval and adult *Drosophila*. Cell Rep. 23, 2524–2531 (2018). 29791860 10.1016/j.celrep.2018.04.085

[43] D. Munch, C. G. Galizia, DoOR 2.0—Comprehensive mapping of *Drosophila melanogaster* odorant responses. Sci. Rep. 6, 21841 (2016). 26912260 10.1038/srep21841 PMC4766438

[44] M. Stopfer, V. Jayaraman, G. Laurent, Intensity versus identity coding in an olfactory system. Neuron 39, 991–1004 (2003). 12971898 10.1016/j.neuron.2003.08.011

[45] M. Knaden, A. Strutz, J. Ahsan, S. Sachse, B. S. Hansson, Spatial representation of odorant valence in an insect brain. Cell Rep. 1, 392–399 (2012). 22832228 10.1016/j.celrep.2012.03.002

[46] E. Gruntman, G. C. Turner, Integration of the olfactory code across dendritic claws of single mushroom body neurons. Nat. Neurosci. 16, 1821–1829 (2013). 24141312 10.1038/nn.3547 PMC3908930

[47] A. Rajagopalan, C. Assisi, Effect of circuit structure on odor representation in the insect olfactory system. eNeuro 7, ENEURO.0130–ENEU19.2020 (2020). 32345734 10.1523/ENEURO.0130-19.2020 PMC7292731

[48] G. C. Turner, M. Bazhenov, G. Laurent, Olfactory representations by *Drosophila* mushroom body neurons. J. Neurophysiol. 99, 734–746 (2008). 18094099 10.1152/jn.01283.2007

[49] S. Dasgupta, D. Hattori, S. Navlakha, A neural theory for counting memories. Nat. Commun. 13, 5961 (2022). 36217003 10.1038/s41467-022-33577-2 PMC9551066

[50] E. S. Schaffer, D. D. Stettler, D. Kato, G. B. Choi, R. Axel, L. F. Abbott, Odor perception on the two sides of the brain: Consistency despite randomness. Neuron 98, 736–742.e3 (2018). 29706585 10.1016/j.neuron.2018.04.004 PMC6026547

[51] A. A. M. Mohamed, T. Retzke, S. Das Chakraborty, B. Fabian, B. S. Hansson, M. Knaden, S. Sachse, Odor mixtures of opposing valence unveil inter-glomerular crosstalk in the *Drosophila* antennal lobe. Nat. Commun. 10, 1201 (2019). 30867415 10.1038/s41467-019-09069-1 PMC6416470

[52] K. Keleman, S. Kruttner, M. Alenius, B. J. Dickson, Function of the *Drosophila* CPEB protein Orb2 in long-term courtship memory. Nat. Neurosci. 10, 1587–1593 (2007). 17965711 10.1038/nn1996

[53] D. S. Manoli, M. Foss, A. Villella, B. J. Taylor, J. C. Hall, B. S. Baker, Male-specific fruitless specifies the neural substrates of *Drosophila* courtship behaviour. Nature 436, 395–400 (2005). 15959468 10.1038/nature03859

[54] A. M. Hermundstad, K. S. Brown, D. S. Bassett, J. M. Carlson, Learning, memory, and the role of neural network architecture. PLOS Comput. Biol. 7, e1002063 (2011). 21738455 10.1371/journal.pcbi.1002063 PMC3127797

[55] S. Dasgupta, T. C. Sheehan, C. F. Stevens, S. Navlakha, A neural data structure for novelty detection. Proc. Natl. Acad. Sci. U.S.A. 115, 13093–13098 (2018). 30509984 10.1073/pnas.1814448115 PMC6304992

[56] A. Litwin-Kumar, K. D. Harris, R. Axel, H. Sompolinsky, L. F. Abbott, Optimal degrees of synaptic connectivity. Neuron 93, 1153–1164.e7 (2017). 28215558 10.1016/j.neuron.2017.01.030 PMC5379477

[57] C. C. Chen, H. W. Lin, K. L. Feng, D. W. Tseng, J. S. de Belle, A. S. Chiang, A subset of cholinergic mushroom body neurons blocks long-term memory formation in *Drosophila*. Cell Rep. 42, 112974 (2023). 37590142 10.1016/j.celrep.2023.112974

[58] T. Miyashita, E. Kikuchi, J. Horiuchi, M. Saitoe, Long-term memory engram cells are established by c-Fos/CREB transcriptional cycling. Cell Rep. 25, 2716–2728.e3 (2018). 30517860 10.1016/j.celrep.2018.11.022

[59] A. Handler, T. G. W. Graham, R. Cohn, I. Morantte, A. F. Siliciano, J. Zeng, Y. Li, V. Ruta, Distinct dopamine receptor pathways underlie the temporal sensitivity of associative learning. Cell 178, 60–75.e19 (2019). 31230716 10.1016/j.cell.2019.05.040 PMC9012144

[60] A. L. Blum, W. Li, M. Cressy, J. Dubnau, Short- and long-term memory in *Drosophila* require cAMP signaling in distinct neuron types. Curr. Biol. 19, 1341–1350 (2009). 19646879 10.1016/j.cub.2009.07.016 PMC2752374

[61] A. Hu, W. Zhang, Z. Wang, Functional feedback from mushroom bodies to antennal lobes in the *Drosophila* olfactory pathway. Proc. Natl. Acad. Sci. U.S.A. 107, 10262–10267 (2010). 20479249 10.1073/pnas.0914912107 PMC2890443

[62] Y. Zhang, T. K. Tsang, E. A. Bushong, L. A. Chu, A. S. Chiang, M. H. Ellisman, J. Reingruber, C. Y. Su, Asymmetric ephaptic inhibition between compartmentalized olfactory receptor neurons. Nat. Commun. 10, 1560 (2019). 30952860 10.1038/s41467-019-09346-z PMC6451019

[63] J. L. Semmelhack, J. W. Wang, Select *Drosophila* glomeruli mediate innate olfactory attraction and aversion. Nature 459, 218–223 (2009). 19396157 10.1038/nature07983 PMC2702439

[64] H. Nishino, M. Iwasaki, M. Paoli, I. Kamimura, A. Yoritsune, M. Mizunami, Spatial receptive fields for odor localization. Curr. Biol. 28, 600–608.e3 (2018). 29429617 10.1016/j.cub.2017.12.055

[65] M. Paoli, H. Nishino, E. Couzin-Fuchs, C. G. Galizia, Coding of odour and space in the hemimetabolous insect *Periplaneta americana*. J. Exp. Biol. 223, jeb218032 (2020). 31932303 10.1242/jeb.218032

[66] S. M. Plaza, J. Clements, T. Dolafi, L. Umayam, N. N. Neubarth, L. K. Scheffer, S. Berg, neuPrint: An open access tool for EM connectomics. Front. Neuroinform. 16, 896292 (2022). 35935535 10.3389/fninf.2022.896292 PMC9350508

[67] R. I. Wilson, Early olfactory processing in *Drosophila*: Mechanisms and principles. Annu. Rev. Neurosci. 36, 217–241 (2013). 23841839 10.1146/annurev-neuro-062111-150533 PMC3933953

[68] J. Lin, Divergence measures based on the Shannon entropy. IEEE Trans. Inf. Theory 37, 145–151 (1991).

[69] H. K. M. Dweck, S. A. M. Ebrahim, M. Thoma, A. A. M. Mohamed, I. W. Keesey, F. Trona, S. Lavista-Llanos, A. Svatoš, S. Sachse, M. Knaden, B. S. Hansson, Pheromones mediating copulation and attraction in *Drosophila*. Proc. Natl. Acad. Sci. U.S.A. 112, E2829–E2835 (2015). 25964351 10.1073/pnas.1504527112 PMC4450379

