# Supplementary material for: Hybrid neural networks in the mushroom body drive olfactory preference in Drosophila
Source: Sci Adv. 2025 May 30;11(22):eadq9893. doi: 10.1126/sciadv.adq9893 (PMC12124391; doi:10.1126/sciadv.adq9893)
Supplement: Supplementary file 1 — Supplementary Methods Figs. S1 to S29 Tables S1 to S10 [file sciadv.adq9893_sm.pdf]

Science Advances
AAAS

Supplementary Materials for

Hybrid neural networks in the mushroom body drive olfactory preference in *Drosophila*

Li-Shan Cheng *et al.*

Corresponding author: Ann-Shyn Chiang, aschiang@life.nthu.edu.tw; Chung-Chuan Lo, cclo@mx.nthu.edu.tw; Ting-Kuo Lee, tklee@phys.sinica.edu.tw

Sci. Adv. **11**, eadq9893 (2025)
DOI: 10.1126/sciadv.adq9893

This PDF file includes:

Supplementary Methods
Figs. S1 to S29
Tables S1 to S10

Supplementary Methods

Spatial clustering for glomeruli

We selected glomeruli from PN cluster 1 and PN cluster 3 to perform the clustering analysis using the Python package Seaborn. The color bar indicates the spatial innervation similarity. The color gradient ranges from 10% (minimum) to 90% (maximum) of the similarity values in the matrix. The pairwise distance is defined by Pearson correlation, and the clustering method is '*single*' to better capture similar glomeruli.

Spatial innervation preference

All neural structures, including neurites, claws, boutons, and synapses, are represented by spatially distributed points in the data. To estimate the spatial innervation preferences between the neural structures (point sets) A and B, we first calculated the distribution density of the sets of points using kernel density estimation (KDE). Next, we determined the spatial distribution similarity (S) between A and B by calculating the JS divergence (see Methods). Then, we quantified the preference score by

$$Pref_{AB} = \frac{S_{AB} - \overline{S_{AB'}}}{\sigma_{AB'}}$$

where S_{AB} is the spatial distribution similarity between A and B. After repeating the categorical shuffling (see Methods) by a number of times, we obtained the mean, $\overline{S_{AB'}}$, and the standard deviation, $\sigma_{AB'}$, of the spatial distribution similarity between A and shuffled $B's$. For example, $Pref_{11}$ represents the spatial innervation preference between the original PN cluster 1 and the shuffled PN cluster 1.

Global shuffling with synaptic weighting

We aimed to incorporate synapse weighting between PNs and KCs during the shuffling process. The unit of shuffling is a KC claw, and as part of this process, the glomerular PNs are reassigned to new downstream KC claws. Throughout the shuffling, we preserved the total synapse number for each claw, as well as the total KC claw number connecting with each PN. The G-to-KC connection table is defined as the sum of the total synapse number between glomerular PNs of the glomerulus and their downstream KCs.

Random null models

We employed three random models, proposed by Zheng et al. *(22)*, to examine the effect of different random models on the observation of connection preferences. In the random bouton null model, each claw of a KC randomly chooses any bouton, regardless of the potential connection limitations of different boutons. In the random glomerulus null model, each claw of a KC randomly chooses any glomerulus, irrespective of its bouton number. In the random claw model, PN-to-KC connections are shuffled while preserving the connection capacity of each bouton during the shuffling process. For the FAFB dataset, we used the data provided in Zheng et al. *(22)*. For the hemibrain dataset, we employed the models based on the connection matrix constructed from the extracted boutons and claws using PN-to-KC synapses (see Methods).

Glomerular output correlation

To determine the relationship between glomeruli, we used Pearson correlation of their output patterns to KCs in the observed network and performed hierarchical clustering. To investigate

whether a glomerular pair preferentially connects to the same KCs, we employed conditional input analysis as proposed by Zheng et al. *(22)*. This involved counting the original frequency of the observed network and calculating the z-scores by comparing it to the frequency of shuffled networks.

Connection specificity estimation

For glomerular projection specificity, the value of a glomerulus is defined as the maximum number of connections to a KC class divided by the total number of connections to all KCs. For receiving specificity of a KC, the value of a KC is defined as the maximum number of connections from a PN cluster divided by the total number of connections from all PN clusters.

Glomerular grouping analysis for PN-to-KC network randomness

To quantify the PN-to-KC connectivity patterns, we analyzed the distribution and proportion of connections from specific glomerular groups to KCs, compared with shuffled connections. The groups were based on PN anatomy in the LH, as detailed in Table S1, with an additional group for the remaining glomeruli not included in groups 1-5. The detailed procedure is as follows *(20)*:

1. Calculate the overall percentage of PN-to-KC connections from each glomerular group.

2. Select KC subsets based on whether they received at least one connection from a specific glomerular group. For example, for KCs receiving at least one input from group 1, the distribution of their remaining connections was compared with the full data and shuffled networks (mean: black dots, error bars: ± s.d.).

This procedure was repeated for all glomerular groups. This approach provides insights into the structured randomness of the PN-to-KC network.

Comparison of Functional Correlation Across Individuals

We collected Pearson's correlation coefficients across all individual pairs based on responses to cVA, BA, PA, EP, and EtOH, and compared them with inter-fly correlations from random models that shuffled odor responses to these odors within each individual.

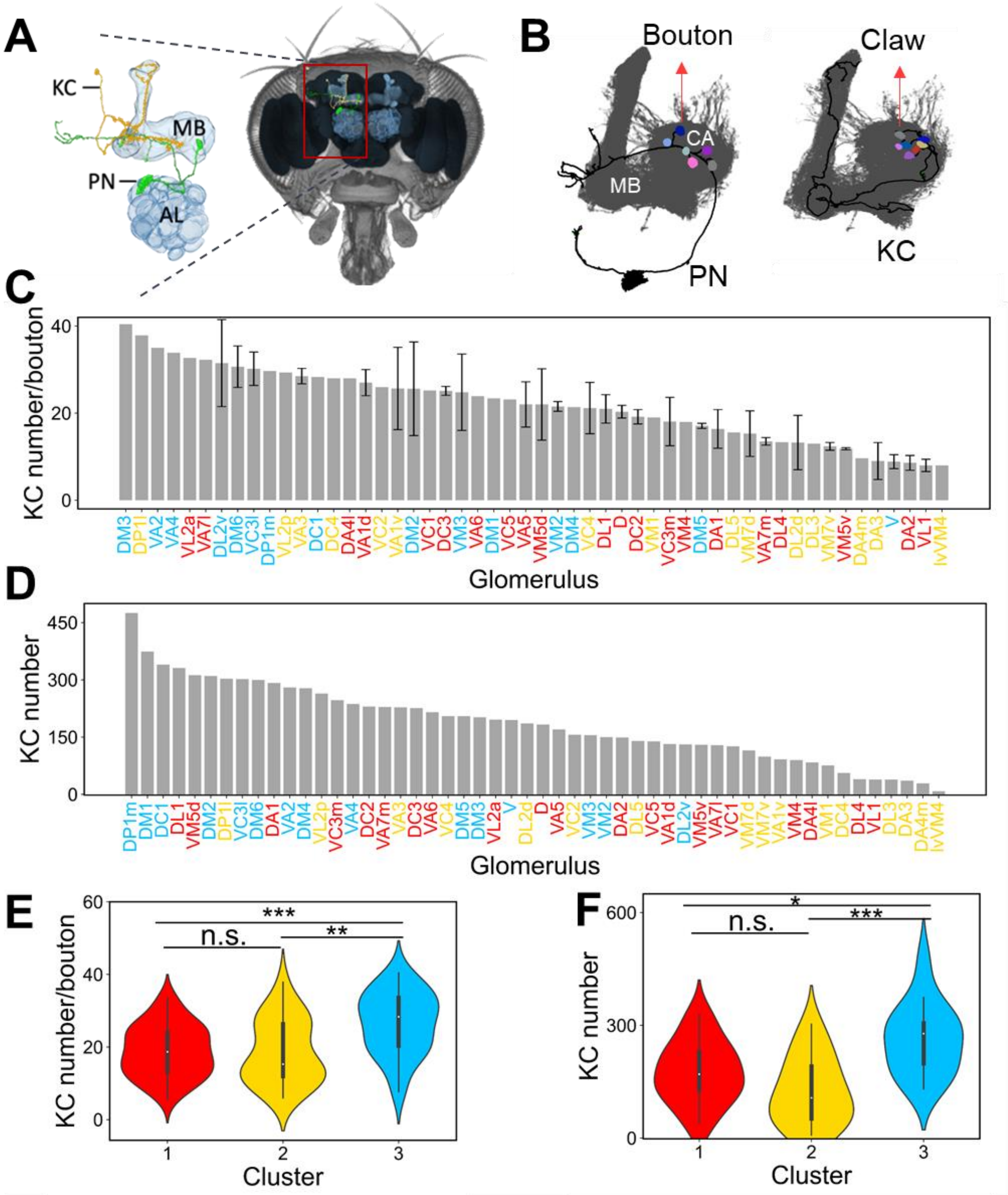

Fig. S1. Differential bouton capacity of glomeruli for KCs. (**A**) A *Drosophila* brain and a zoom-in view of the AL and MB. An example of a synaptic contact of a PN (green) and a KC (yellow) in CA. (**B**) Identified boutons from a PN (left) and claws from a KC (right), using PN-to-KC synapses (see Methods). (**C**) The average number of downstream KCs connected by each bouton from a given glomerulus. The color of each glomerulus is assigned by cellular connection preferences in Fig. 1B. Each error bar represents the standard deviation across individual PNs from the given glomerulus. (**D**) The total downstream KCs connected by each glomerulus. The color code is the same as in (C). (**E-F**) The distribution of the number of connected KCs per bouton (E) and per glomerulus (F) in PN clusters. (Kruskal-Wallis one-way ANOVA with Bonferroni's post hoc test: n.s.: not significant, *: $p < 0.05$, **: $p < 0.01$, and ***: $p < 0.001$.).

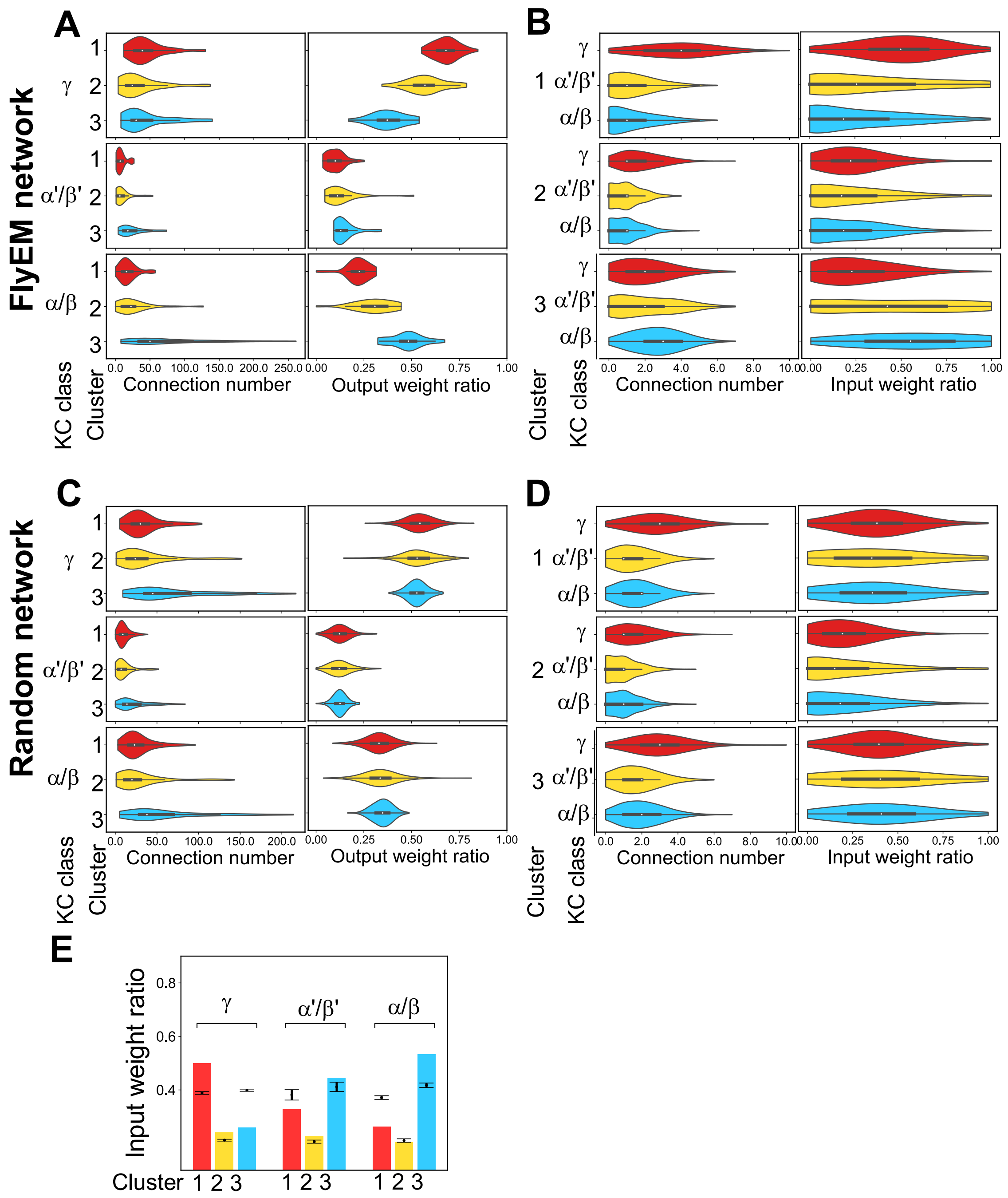

Fig. S2. Summary of PN-to-KC connections. The distributions of PN connection numbers and output weight ratios to each KC class for PNs in each cluster are shown. The output weight ratio is defined as the weight of a PN to a KC class divided by the total weight of the PN to all KCs (see Methods). (**B**) Similar to (A**)** but from the KC perspective. The connection numbers represent the number of PNs from which a KC receives input. The input weight ratio is defined as the weight of a PN cluster to a KC divided by the total weight for the KC from all PNs (see Methods). Part of the data is displayed in Fig. 1C. (**C**) and (**D**) are the same as (A) and (B), respectively, but for shuffled networks, preserving the connection numbers for each PN and KC. (**E**) The input weight ratio of three KCs from PN clusters. The error bar indicates the result from 30 trials of shuffling.

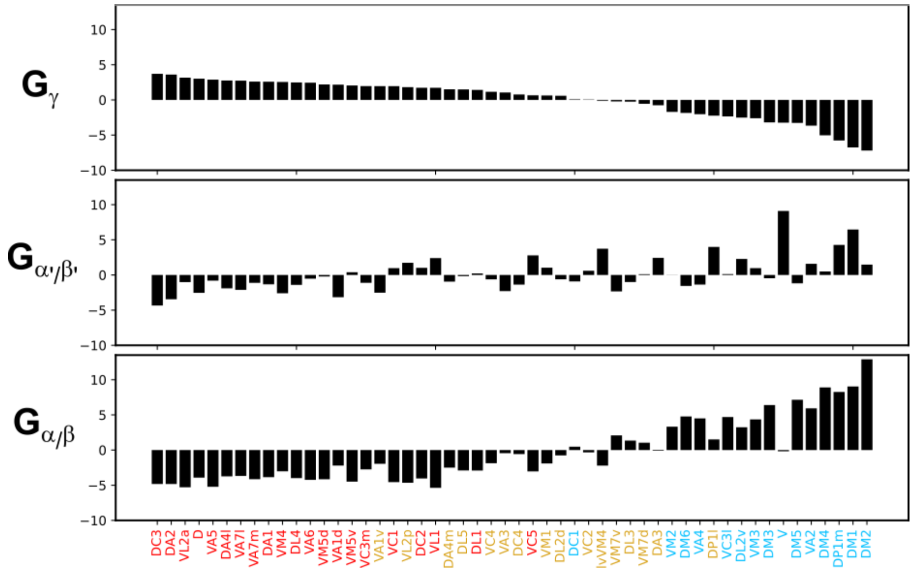

Fig. S3. The preference score G_i for each glomerulus with the synaptic numbers taken into consideration. Instead of counting each connection between a PN and a KC as one (as in Fig. 1A), in this plot, the connection between a pair of PN and KC is weighted by the number of synapses formed between them. Three preference clusters are color-labeled with red (cluster 1), yellow (cluster 2), and blue (cluster 3).

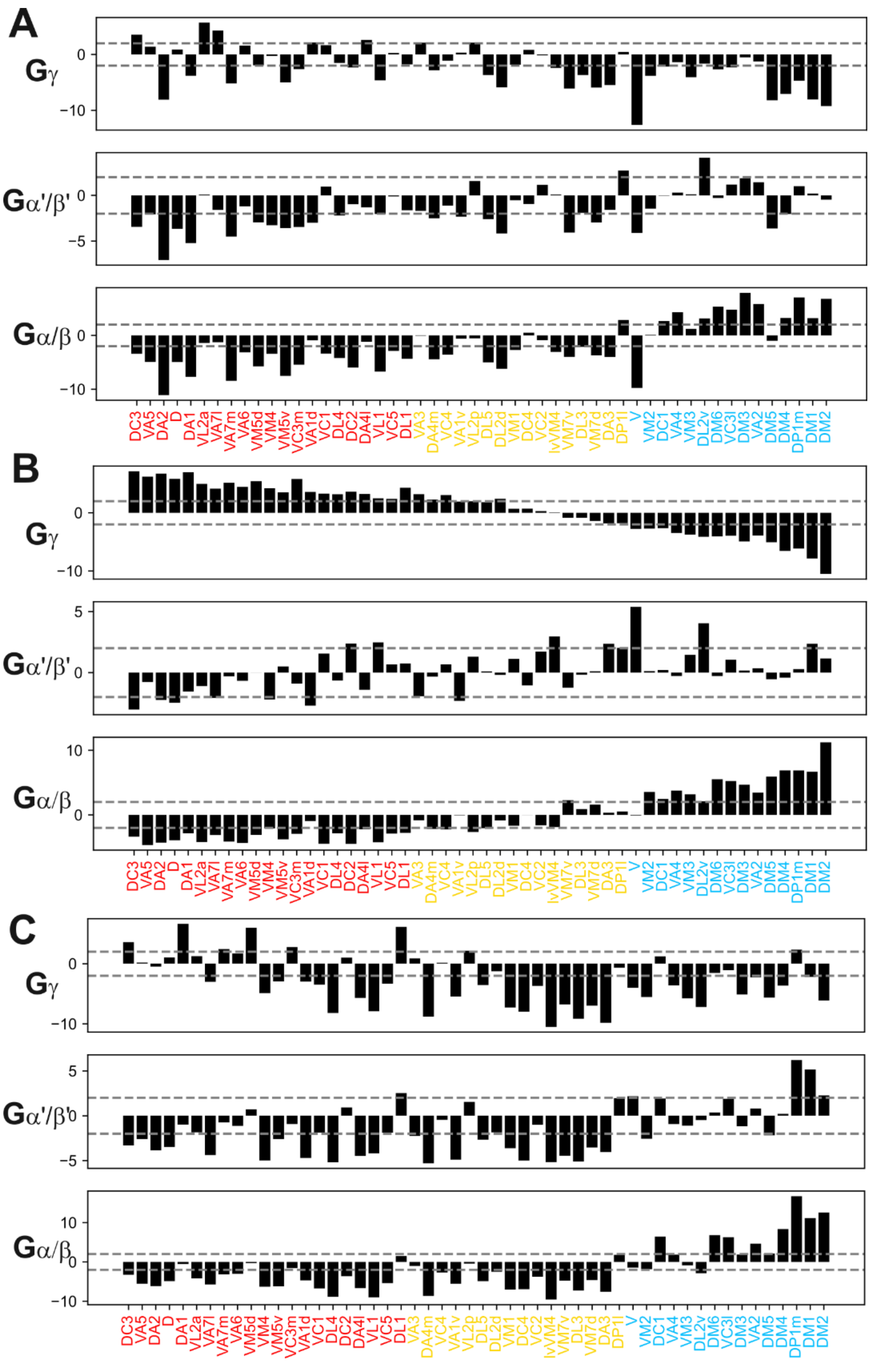

Fig. S4. Application of various random models on the hemibrain dataset. (**A-C**) The preference scores derived from the comparisons between the observed hemibrain network and the shuffled networks (n = 1000) based on the random bouton null model (A), the random claw null model (B), and the random glomerulus model (C), as suggested by Zheng et al. *(22)* (see Supplementary Methods). The glomeruli are arranged and color-labeled as in Fig. 1B. Dashed lines represent z-scores above 2 or below -2, respectively.

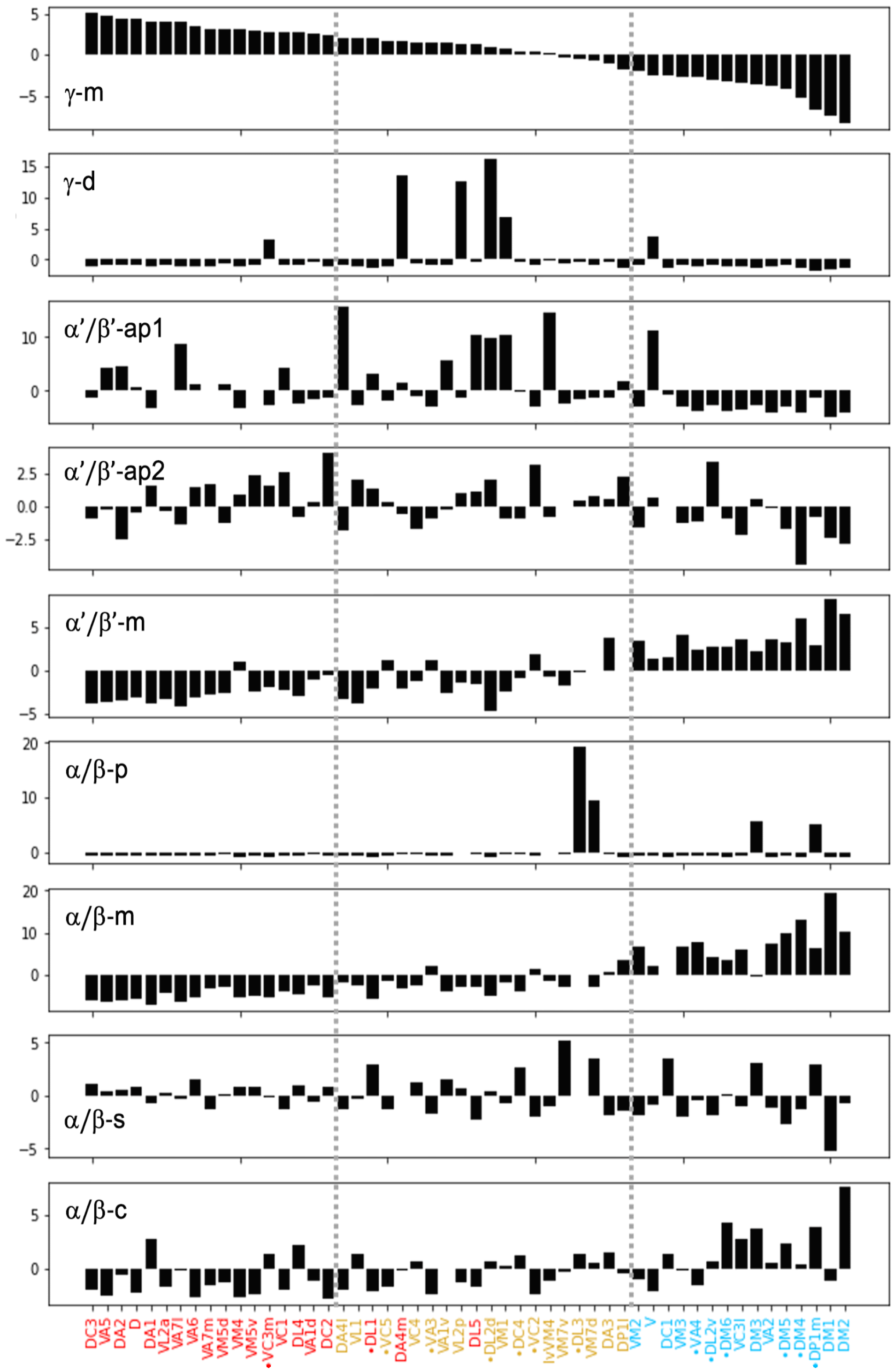

Fig. S5. The preference score of KC subclasses. The glomeruli are sorted by the preference score values of γ-m. Three preference clusters are color-labeled in red (cluster 1), yellow (cluster 2), and blue (cluster 3).

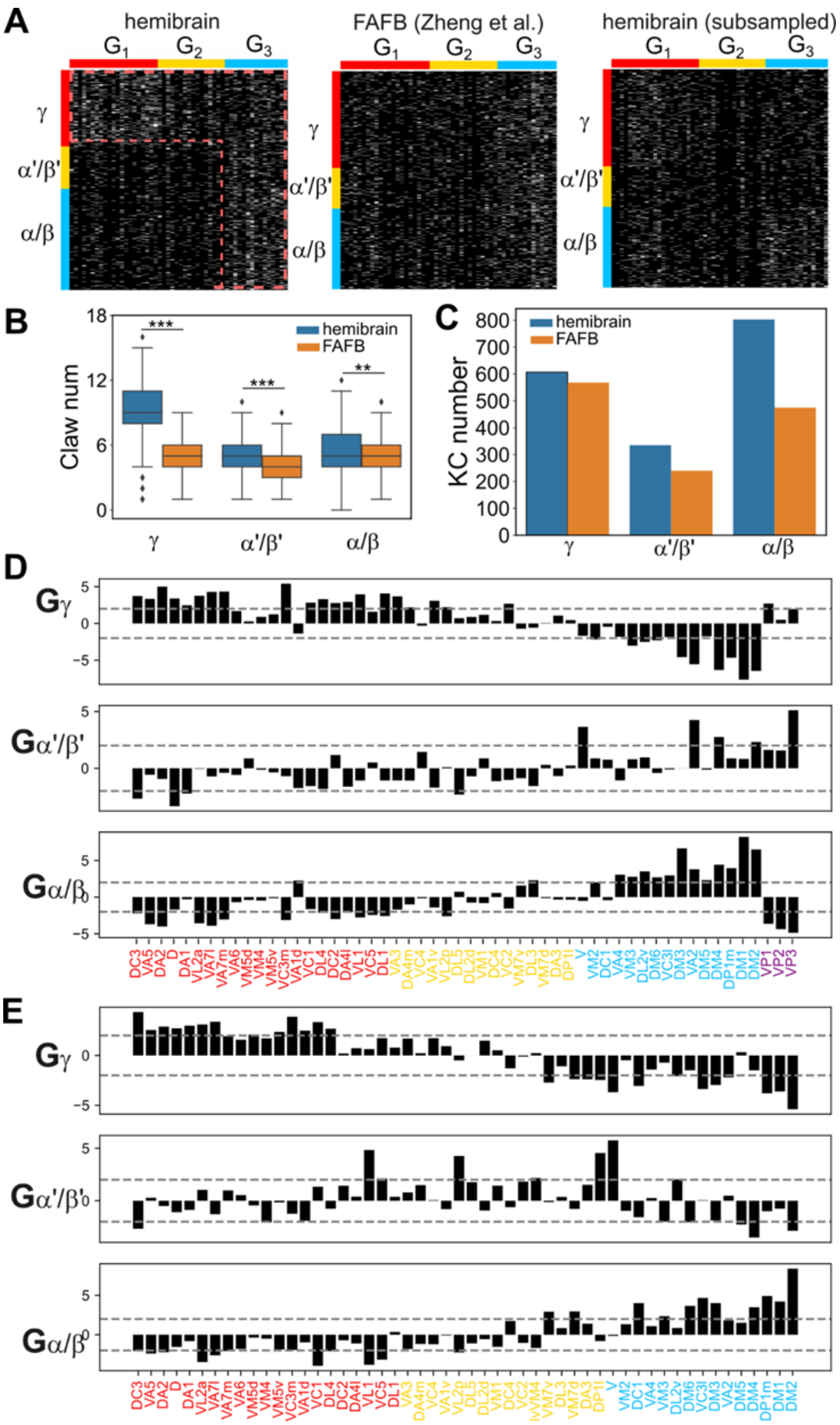

Fig. S6. Comparative overview of the hemibrain dataset and the FAFB dataset. (**A**) The G-to-KC connection table based on bouton and claw of the original hemibrain dataset (left), the FAFB dataset (middle), and the subsampled hemibrain dataset (right). (**B-C**) The comparison of claw numbers (B) and neuron numbers (C) in each KC class between the two datasets. (Mann Whitney

U test: n.s.: not significant, *: $p < 0.05$, **: $p < 0.01$, and ***: $p < 0.001$). (**D**-**E**) The preference scores of the FAFB dataset (D) and the subsampled hemibrain dataset (E) compared with their shuffled networks (n = 1000) based on the random claw model. The glomeruli are arranged and color-labeled as in Fig. 1B. Dashed lines represent z-scores above 2 or below -2, respectively.

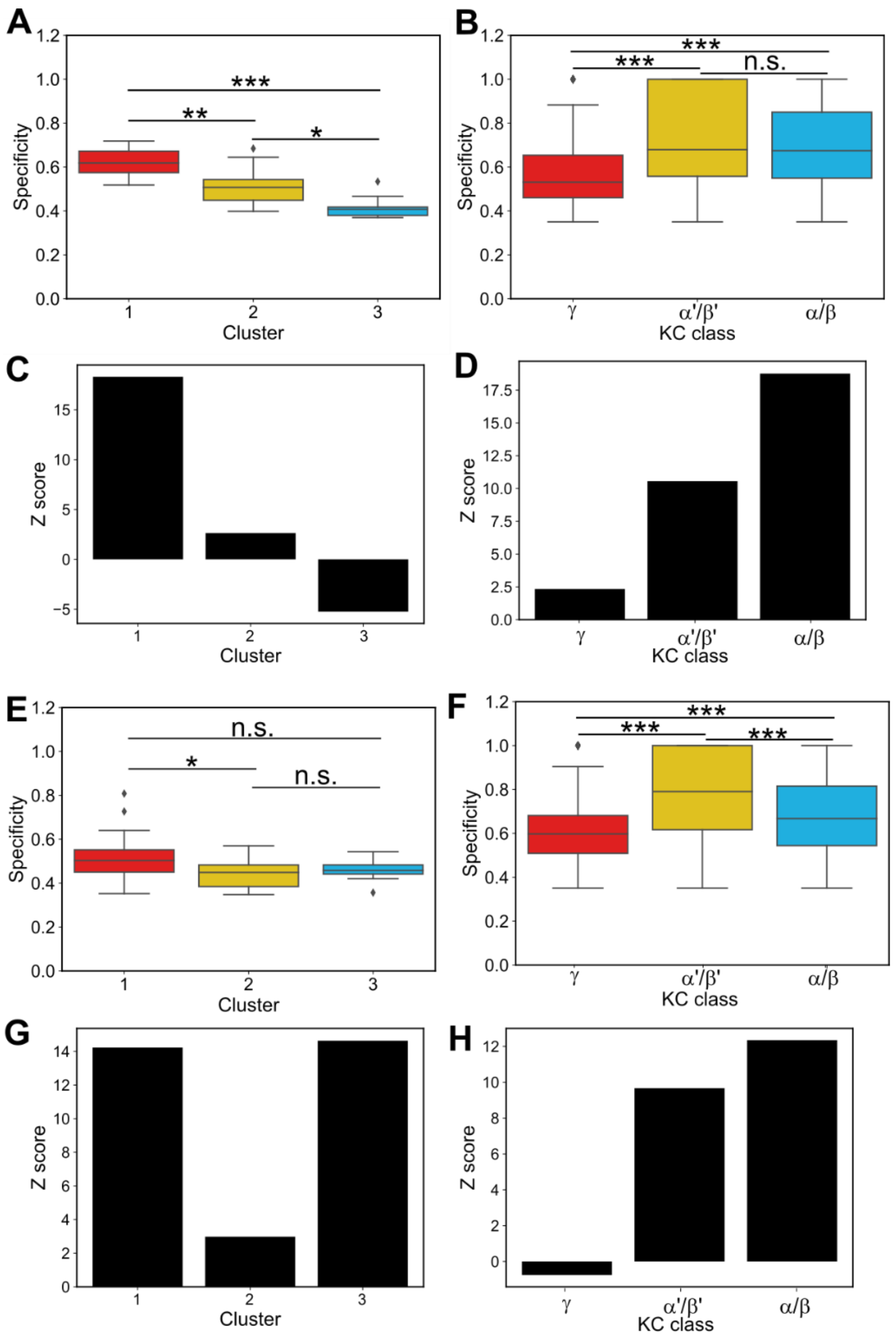

Fig. S7. Connection specificity of PN clusters and KC classes. (**A**) Glomerular projection specificity of PN clusters to a single KC class. (**B**) Receiving specificity of KCs from a single PN cluster. (**C**) Z-score comparisons of observed glomerular projection specificity to shuffled networks with preserved connection capacities. (**D**) Z-score comparisons of observed KC class receiving specificity to shuffled networks with preserved connection capacities. (**E-H**) Similar to (A-D) but for the FAFB dataset, respectively. (Kruskal-Wallis one-way ANOVA with Bonferroni's post hoc test: n.s.: not significant, *: $p < 0.05$, **: $p < 0.01$, and ***: $p < 0.001$.).

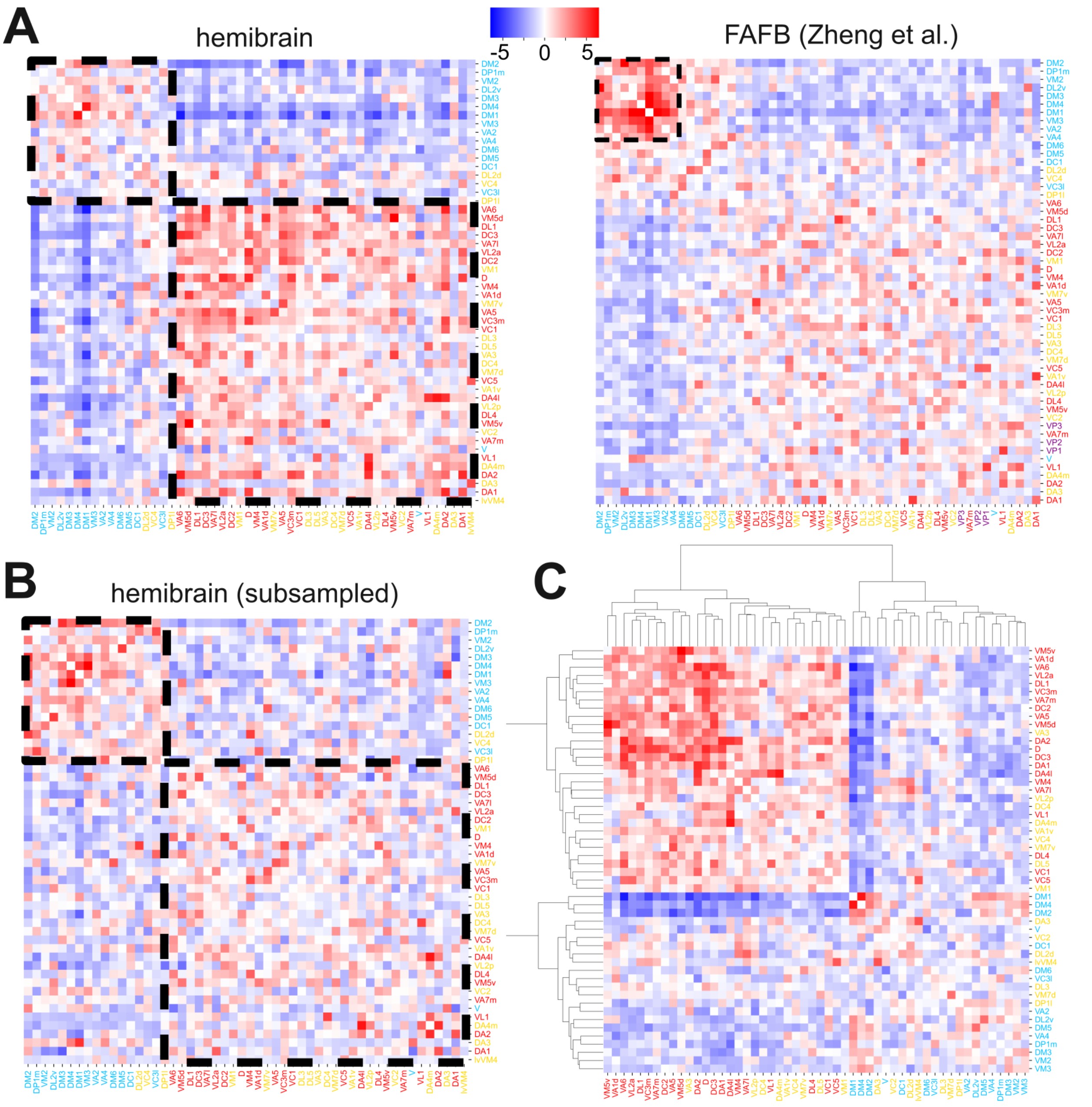

Fig. S8. Comparison of glomerular conditional input preference between different datasets. (**A**) The correlation matrix of the hemibrain dataset (left) and the FAFB dataset (right) based on the conditional input analysis (see Supplementary Methods) which compared the original ones with the random claw null models, respectively. These results were presented in Zheng et al. *(22)*. We remade the figures to show the classification based on our results in Fig. 1B. (**B**) Similar to (A) but for the subsampled hemibrain dataset. In (A-B), Glomeruli are arranged in the same order as in Zheng et al. *(22)*. We compared the result of subsampled hemibrain data in (B) with the results in (A). (**C**) The correlation matrix in (A) with glomeruli re-ordered by hierarchical clustering. The cluster 1 and cluster 3 glomeruli can be categorized into two separate groups.

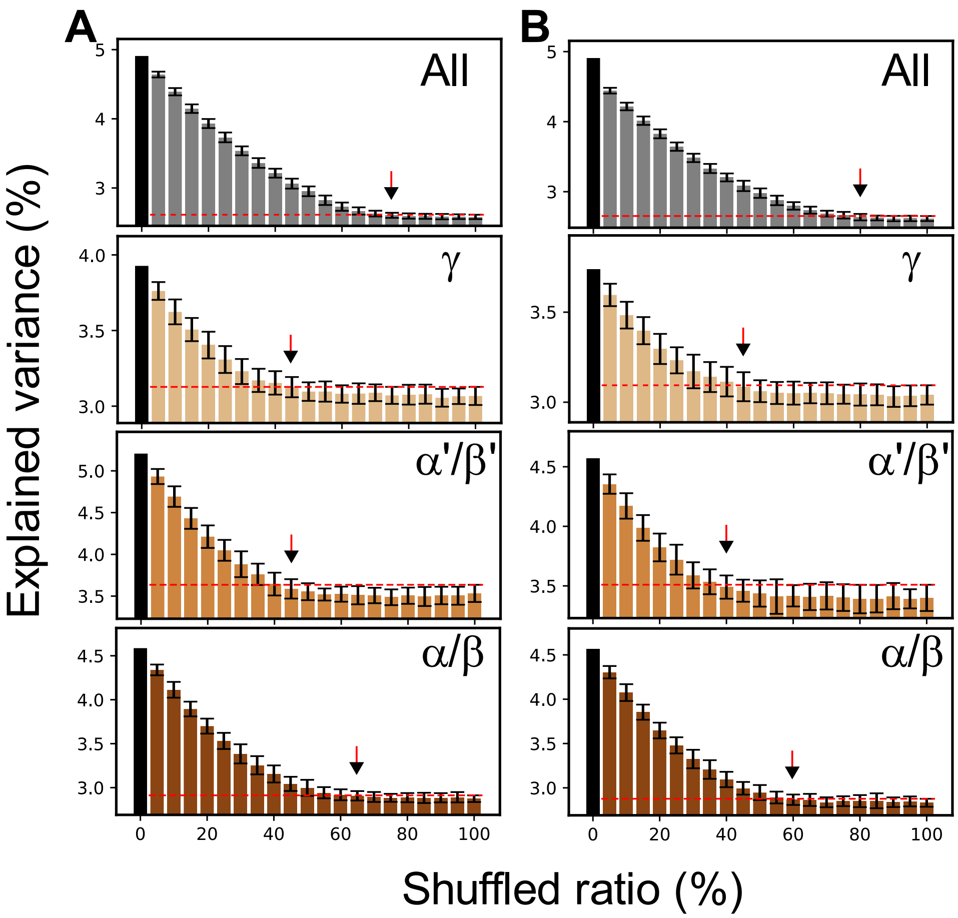

Fig. S9. The non-random structure in the PN-to-KC network as revealed by random shuffling. (**A**) The explained variance of the first principal component of the PN-to-KC networks containing all KCs, γ, α'/β', or α/β KCs (from top to bottom) with partially shuffled connections. The results imply a non-random structure in the PN-to-KC network. The error bars indicate the standard deviation of the explained variance from 1000 shuffling trials. The red dashed line indicates the upper standard deviation cap under 100% shuffling. Arrows mark the shuffled ratios that are required to remove the non-random structure. (**B**) Same analysis as in (A), but with the connections weighted by the synaptic numbers between PNs and KCs.

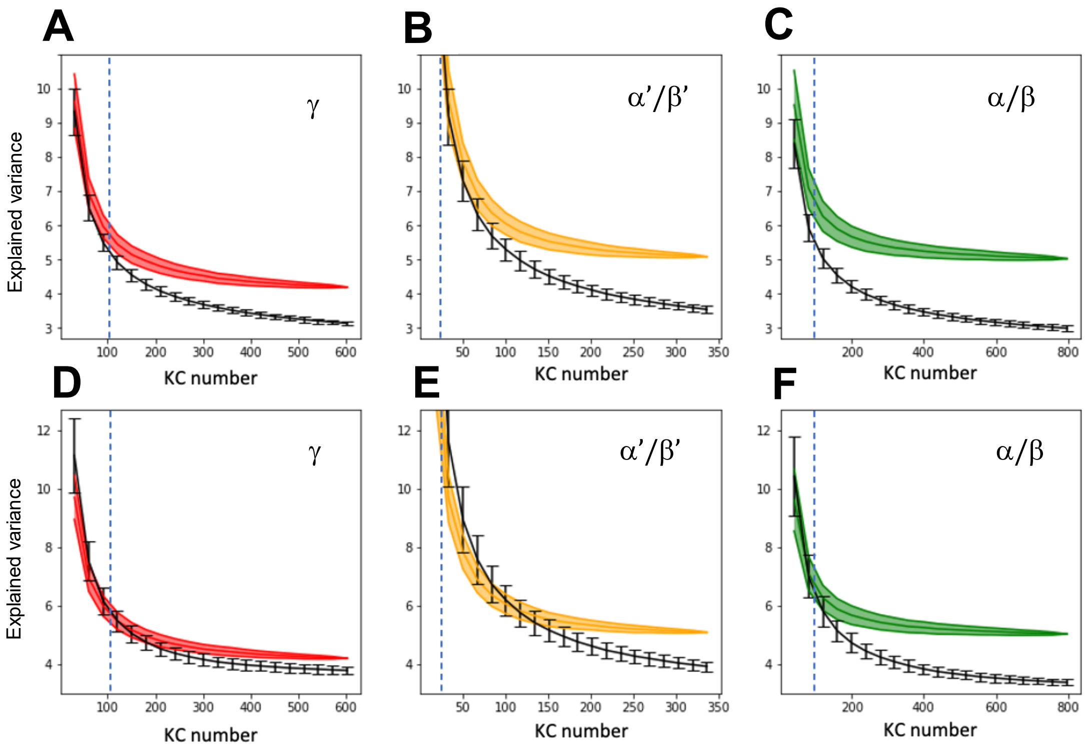

Fig. S10. Partial sampling of the KCs is less likely to reveal the non-random structure in the PN-to-KC network. (**A**-**C**) Comparison of the explained variance of the first principal component (PC1) (y-axis) between the PN-to-KC network (colored curves) and the shuffled network (black curves) under the same degree of partial sampling (x-axis). The total KC number and the connection number of each KC and each glomerulus are preserved during shuffling. The color-filled region represents the mean value ± s.d. of the explained variance from 1000 sampling trials. The error bar represents the mean value ± s.d. of the percent variance from 1000 shuffling trials. The blue dashed lines mark the sampling sizes in Caron et al. *(20)*. (**D**-**F**) The same procedure is applied on the partially sampled PN-to-KC network and shuffled networks, but with 50% of the connections randomly excluded. The results show that a large sampling size is required to discern the difference between a partially sampled network and shuffled networks.

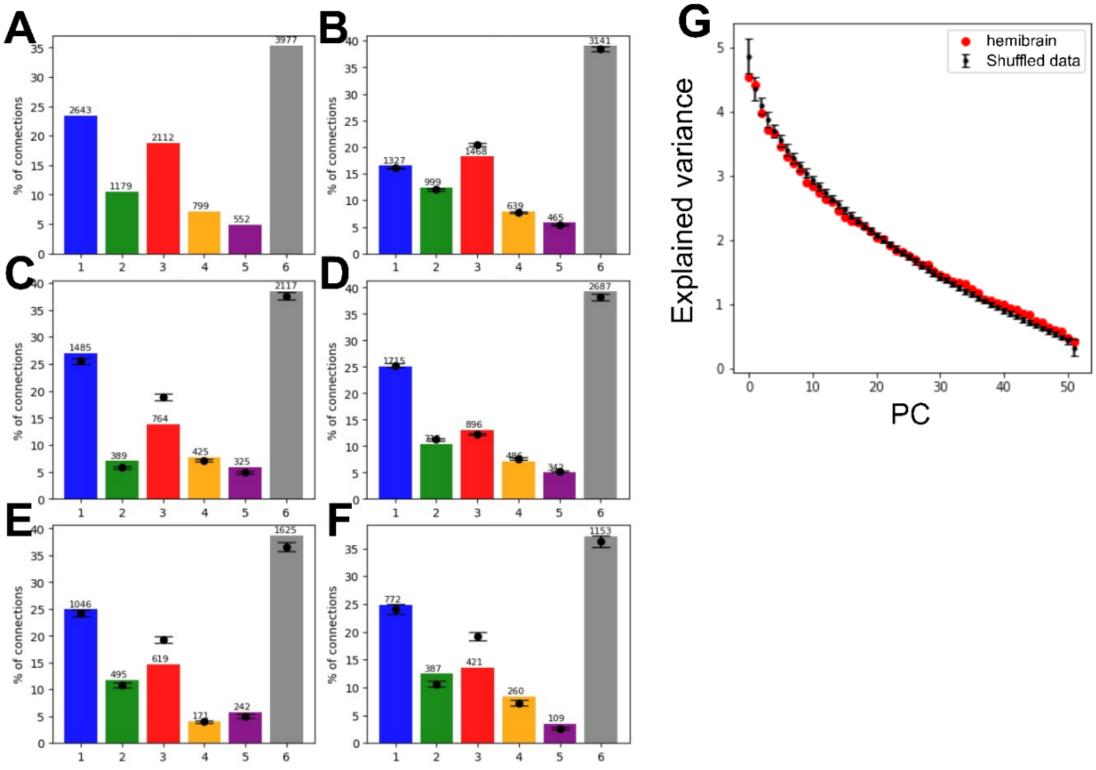

Fig. S11. Random-like network structure due to incomplete sampling of the PN-to-KC network. (**A**) The percentage of PN connections to all KCs is shown for each glomerular group (see Supplementary Methods and Table S1), with the number of connections labeled above each bar. (**B**) Similar to (A), but showing PN connections to the subset of KCs that receive at least one connection from glomerular group 1. Shuffled network results are represented by dots (mean) and error bars (s.d.). (**C–F**) Similar to (B), showing PN connections to subsets of KCs that receive at least one connection from glomerular groups 2–5, respectively. (**G**) The variance explained by each principal component (PC) in PCA for the partially sampled PN-to-KC network, where 50% of the input was dropped out, is shown for subsets of KCs (102 γ KCs, 14 α'/β' KCs, and 84 α/β KCs). The variance distribution of these subsampled connections is indistinguishable from the shuffled connections.

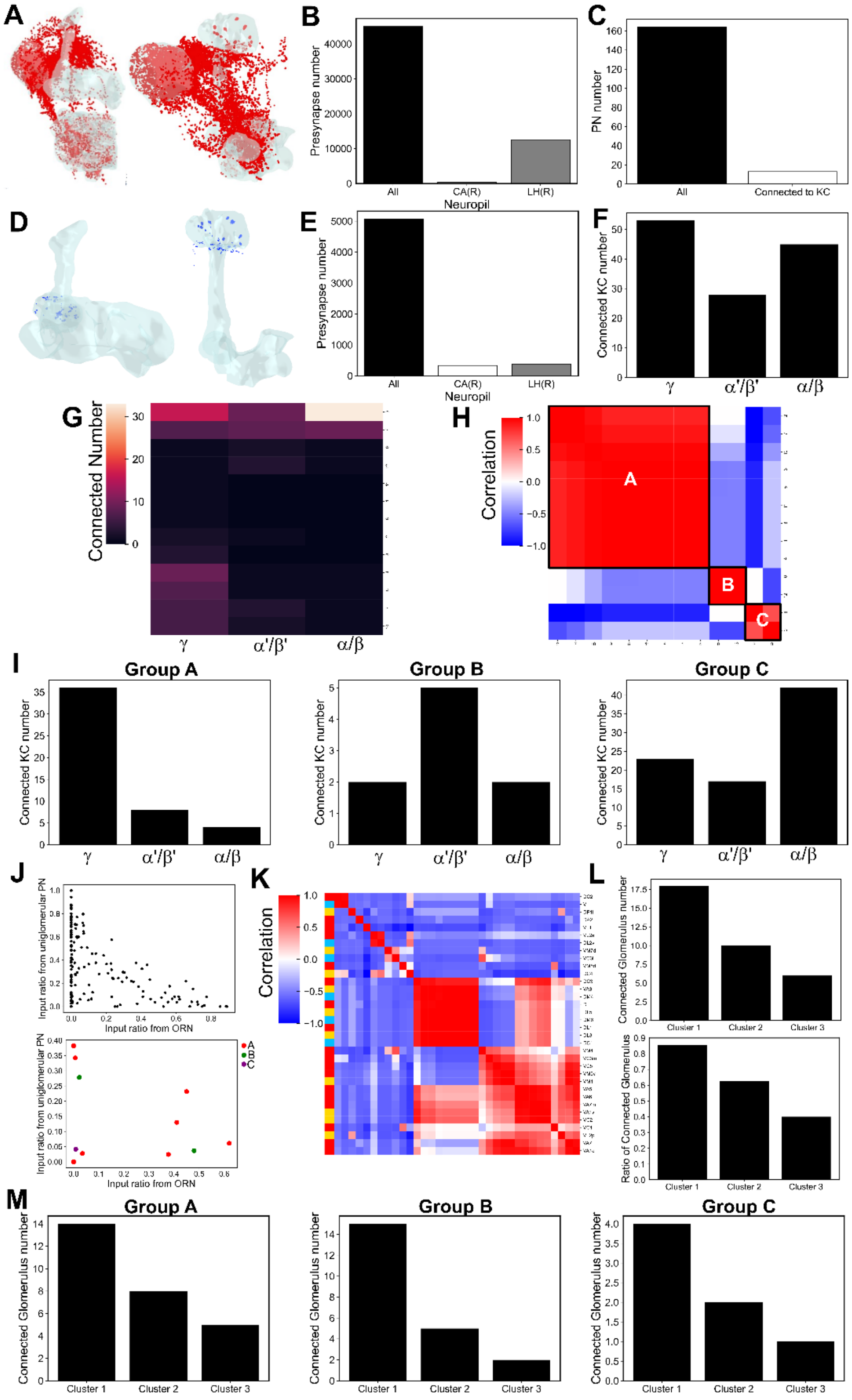

Fig. S12. Input and output patterns of multi-glomerular PNs. (A) Spatial distribution of presynapses of multi-glomerular PNs. **(B)** Number of presynapses in different neuropils. **(C)** Number of multi-glomerular PNs connected to KCs. **(D)** Spatial distribution of presynapses of

multi-glomerular PNs projecting to KCs. **(E)** Same as (B), but only showing neurons connected to KCs. **(F)** Number of KCs in different KC classes receiving inputs from multi-glomerular PNs. **(G)** Connection matrix of each multi-glomerular PN to KC classes. **(H)** Correlation matrix of multi-glomerular PNs based on their connection patterns to KCs. **(I)** Same as (F), but displayed for the three groups of multi-glomerular PNs. **(J)** Input weight ratio of ORNs and uni-glomerular PNs. The upper panel shows all multi-glomerular PNs, while the lower panel displays only those connected to KCs. **(K)** Correlation matrix of glomeruli based on their output patterns to multi-glomerular PNs connected to KCs. **(L)** Number of glomeruli in each cluster connected by multi-glomerular PNs. **(M)** Same as (L), but showing the number of connected glomeruli for each type of multi-glomerular PN, respectively.

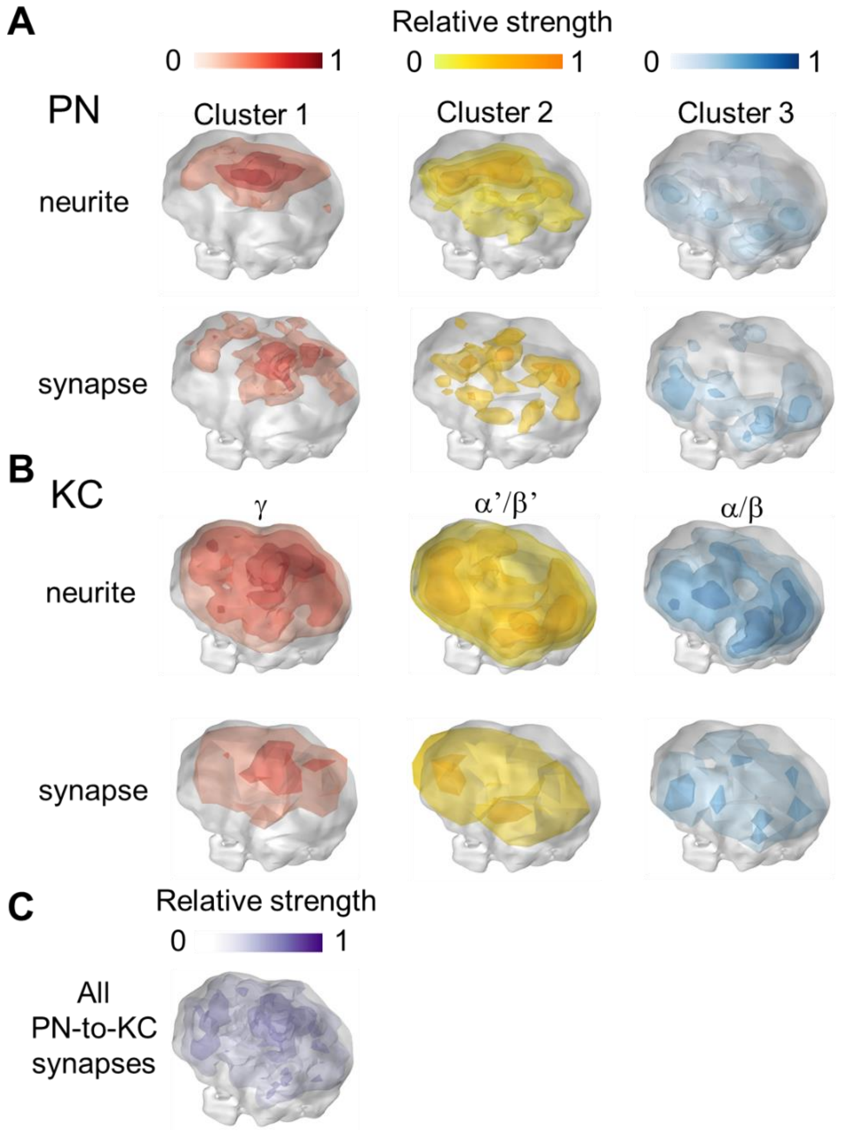

Fig. S13. Spatial innervation density of PN clusters and KC classes in CA. (**A**) The density map of PN neurites (top) and PN-to-KC synapses (bottom) for three PN clusters (from left to right). (**B**) Same as in (A) but for three KC classes. (**C**) The density map of all PN-to-KC synapses.

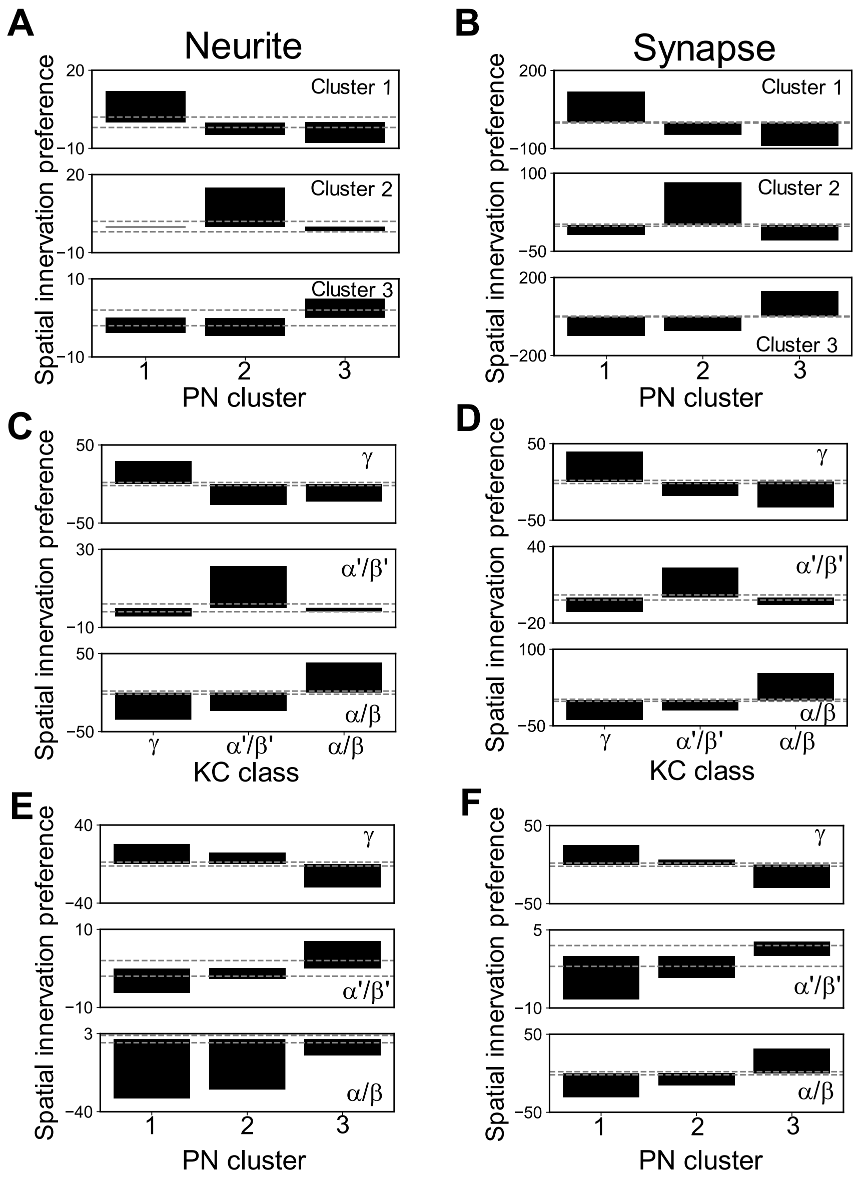

Fig. S14. Spatial innervation preference comparison among PN clusters and KC classes. The spatial innervation preference (S) measures the normalized overlap between two sets of structures (e.g., neurites of PN clusters 1 and 2) with respect to the shuffled data (n = 30, see Supplementary Methods). (**A**) Spatial innervation preferences computed based on neurites from the three PN clusters. (**B**) Same as (A) but for synapses. (**C**) and (**D**) Same as (A) and (B) but for three KC classes. (**E**) and (**F**) Similar to (A) and (B) but for the spatial innervation preferences between PN clusters and KC classes. For each plot, the upper and lower dashed lines indicate the preference scores of 2 and -2, respectively.

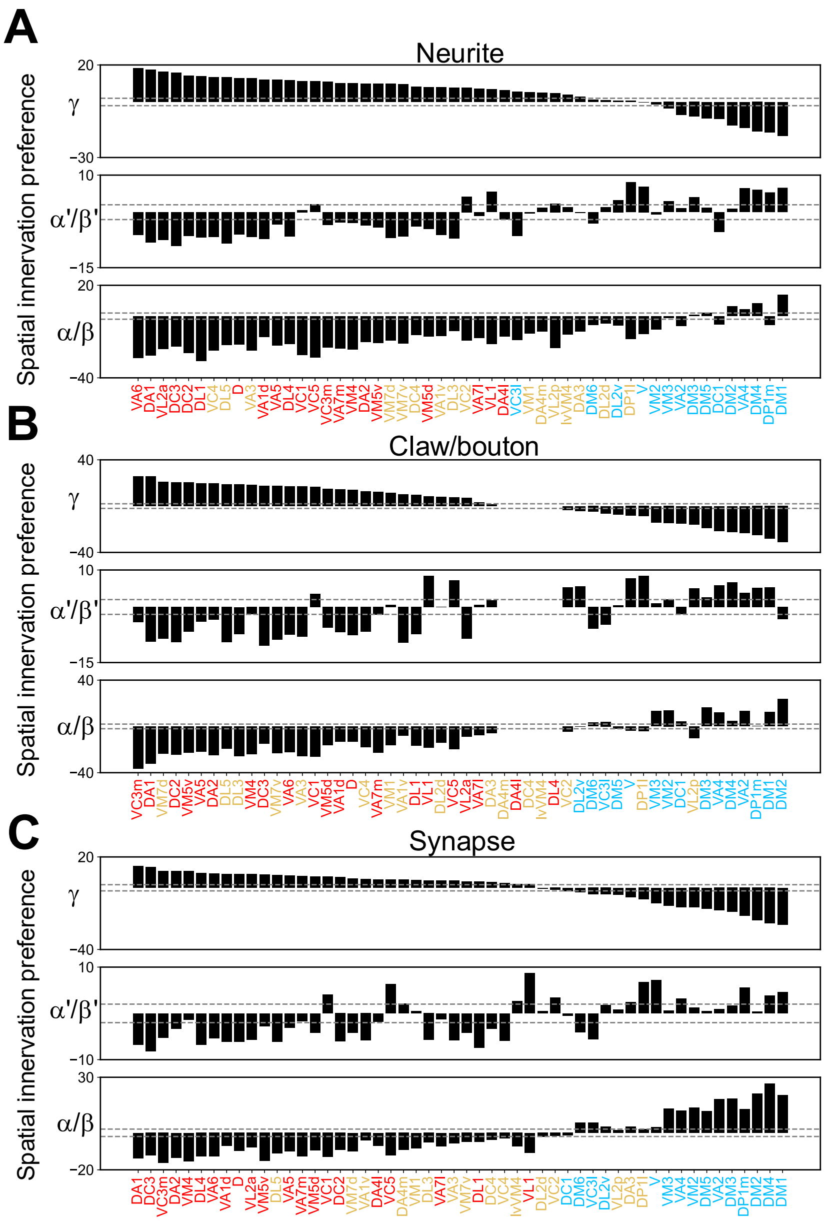

Fig. S15. Spatial innervation preference between glomeruli and KC classes. (**A**) Spatial innervation preferences (S, n = 30 for shuffled networks, see Supplementary Methods) computed based on neurites. (**B**) Same as (A) but for claws/boutons. (**C**) Same as (B) but for PN-to-KC synapses. The color assignment of each glomerulus is the same as in Fig. 1B. The glomeruli are ordered based on the descending preference scores for γ. For each plot, the upper and lower dashed lines indicate the preference scores of 2 and -2, respectively.

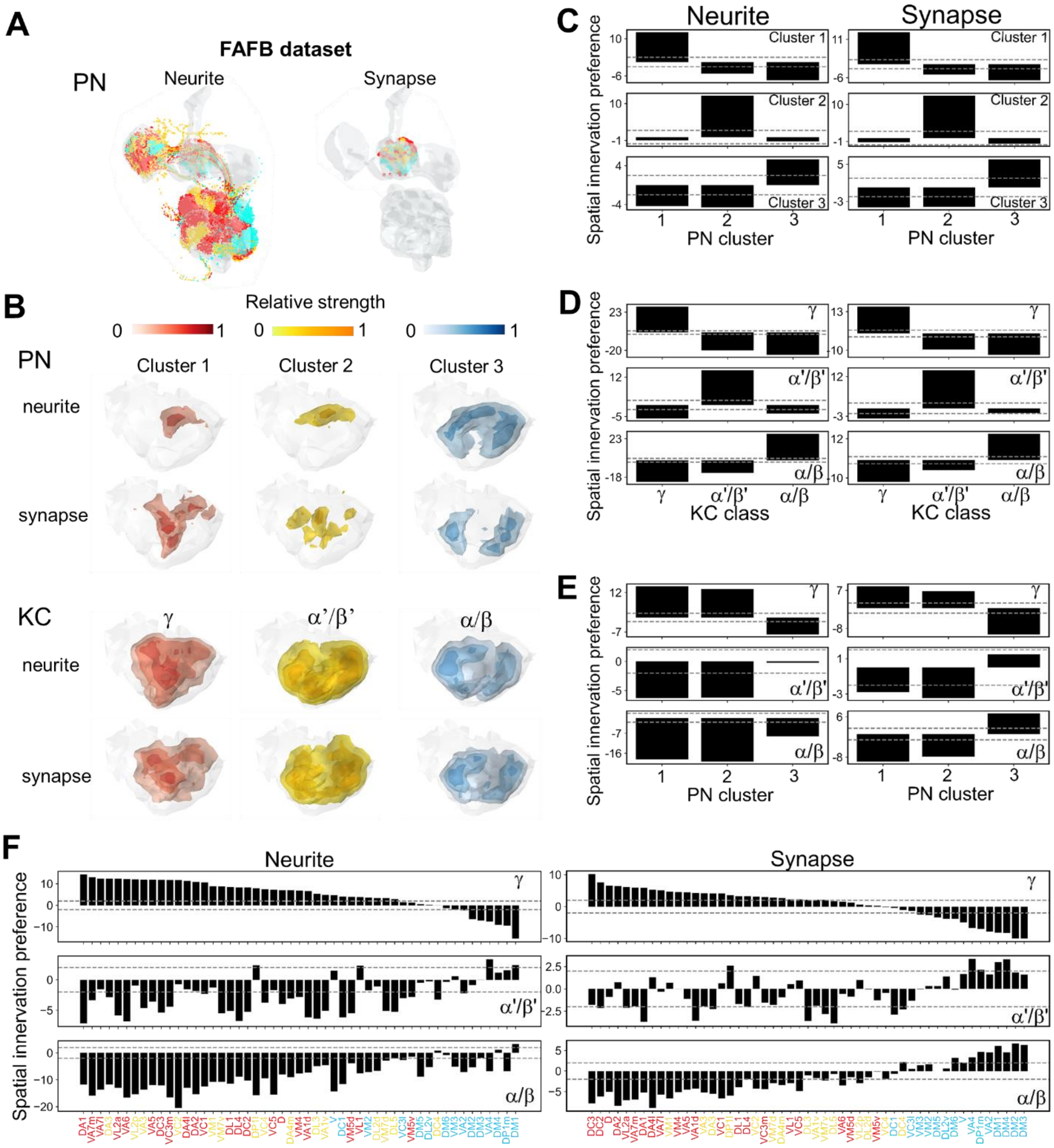

Fig. S16. Spatial innervation analysis of PN clusters and KC classes using the FAFB dataset. (**A**) Spatial innervation contours of PN clusters. The three PN clusters are represented in three different colors, as in Fig. 1B. PN cluster 3 projects to the ventral region of the calyx, while PN cluster 1 projects to the dorsal regions of the calyx. (**B**) Innervation densities of neurites and PN-to-KC synapses for PN clusters and KC classes. (**C-E**) Spatial innervation preferences of neurites (left) and synapses (right) compared within PN clusters (C), KC classes (D), and between PN clusters and KC classes (E). (**F**) Spatial innervation preferences of neurites (left) and synapses (right) are shown between glomeruli and KC classes, with glomeruli ordered based on descending preference scores for γ. Glomeruli are colored according to PN clusters as in Fig. 1B. (For each shuffling experiment, n = 30 for shuffled networks)

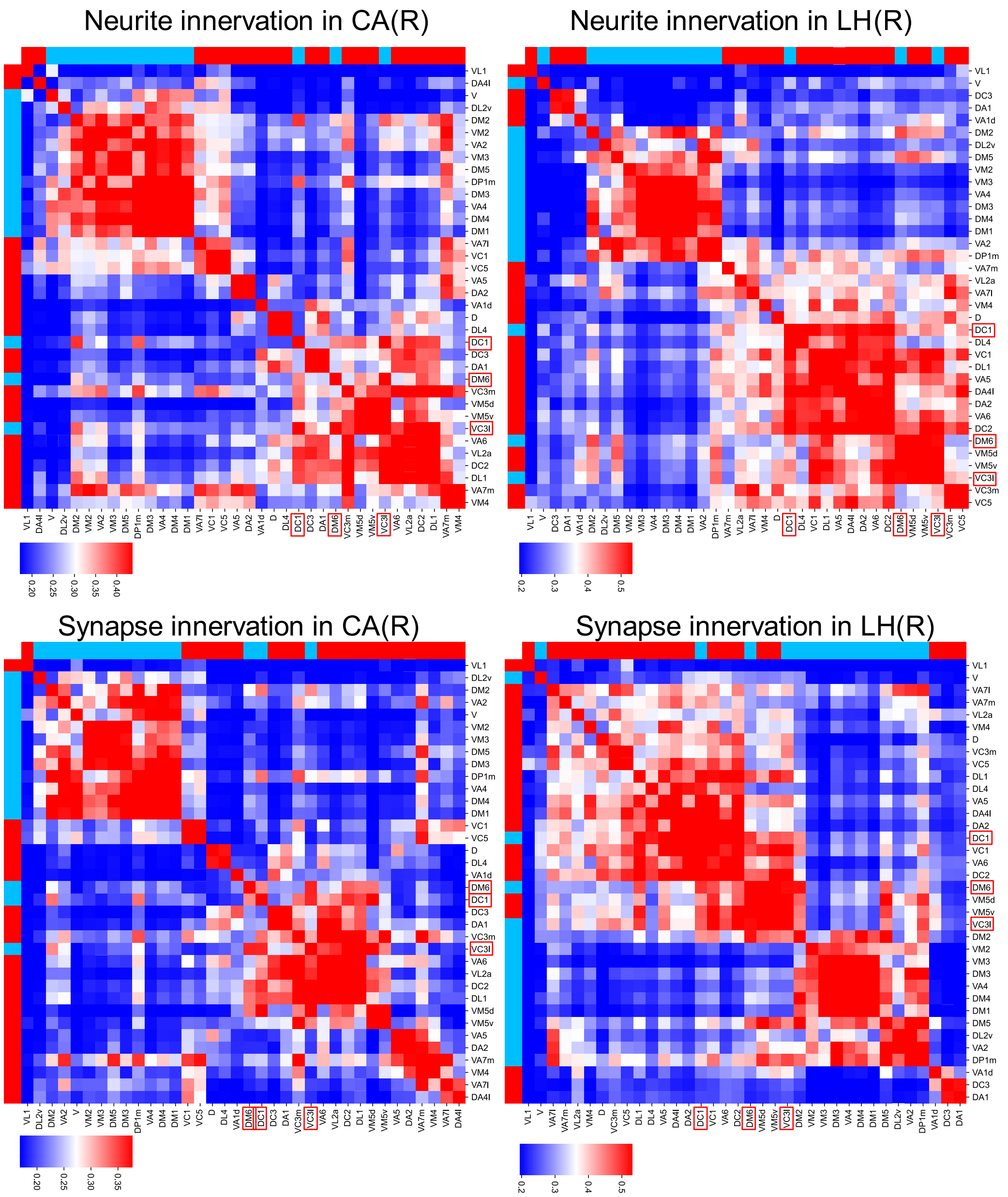

Fig. S17. Spatial segregation of PNs in CA and LH. We computed the spatial innervation similarity (see Supplementary Methods) between glomeruli in the CA (left column) and in the LH (left column) for PN clusters 1 and 3. The spatial innervation similarity is computed based on neurites (top row) and synapses (bottom row). The top row and leftmost column in each matrix indicate PN clusters (red for cluster 1 and blue for 3). PN cluster 1 and PN cluster 3 are segregated for most glomeruli in both CA and LH. The spatial innervation pattern of glomeruli, DC1, VC3l, and DM6 (indicated by red frames) in cluster 3, is different from other cluster 3 glomeruli in both CA and LH. The color gradient bar represents the range of spatial innervation similarity values, spanning from the 10th percentile (minimum) to the 90th percentile (maximum) of the similarity distribution among all pairs.

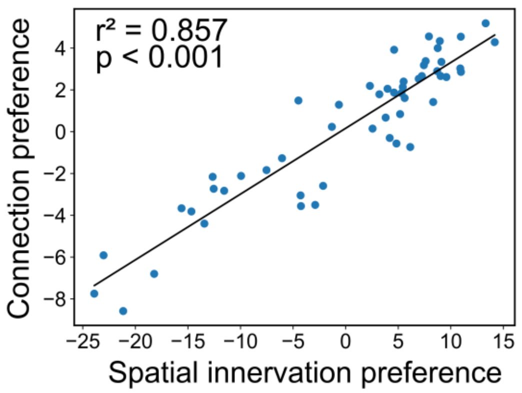

Fig. S18. Spatial innervation preference of glomeruli and KC classes is highly consistent with PN-to-KC connection preferences. The connection preference (G_γ, in Fig. 1B) is strongly correlated with the synaptic spatial innervation preference compared with γ (S_γ, in fig. S15C). ($r^2 = 0.857$, $p < 0.001$)

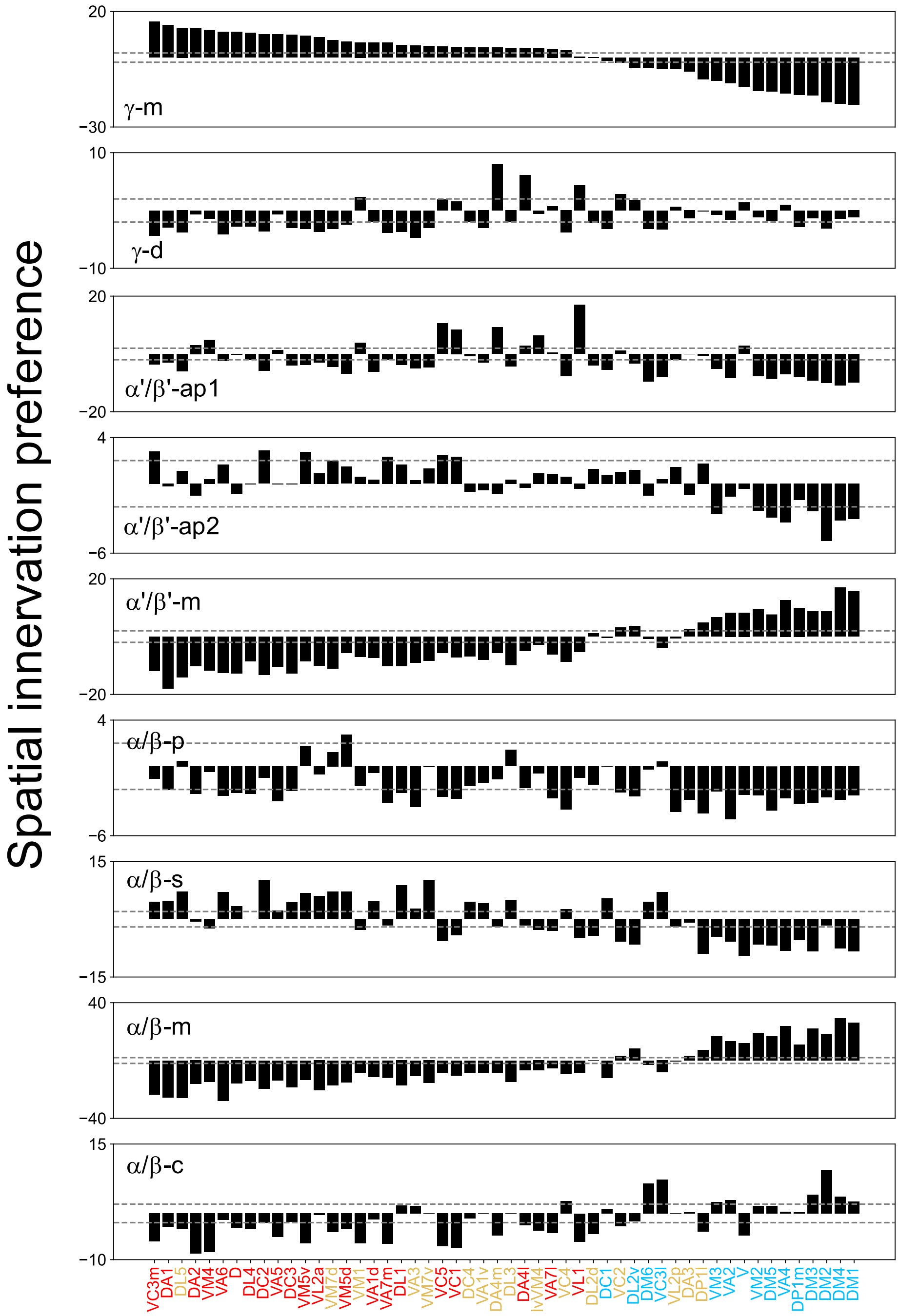

Fig. S19. Synaptic spatial innervation preference between glomeruli and KC subclasses. The glomeruli are ordered based on the descending preference scores for γ-m. The glomeruli are colored based on the PN clusters as in Fig. 1B. (n = 30 for shuffled networks)

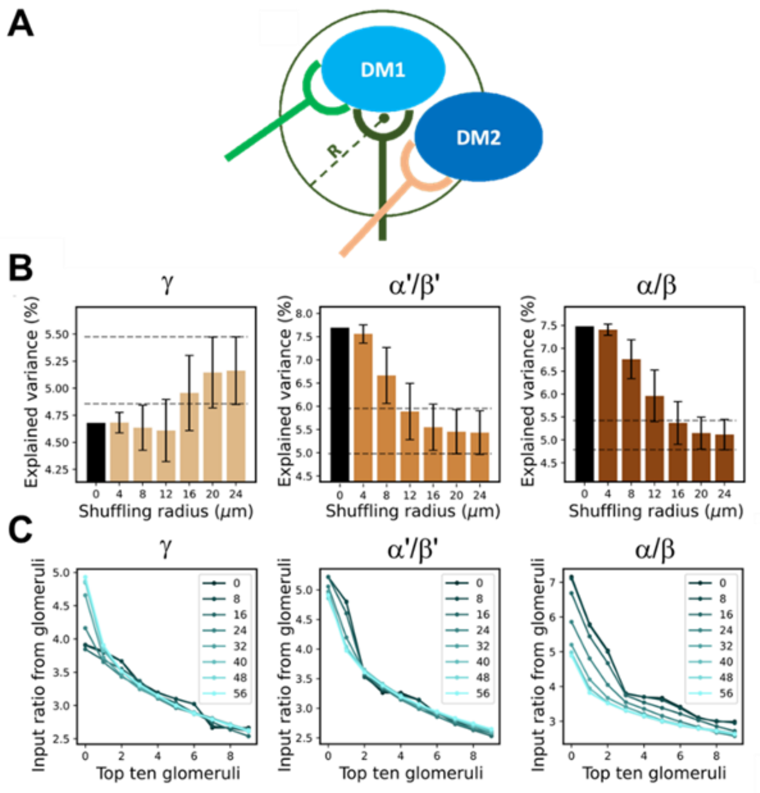

Fig. S20. The spatial stereotype of the calyx confines the glomerular input diversity of KC classes. (**A**) The schematic plot of the local shuffling algorithm. Two blue ovals illustrate the boutons from different PNs, and the forceps represent the KC claws. The claws with a distance shorter than *R* randomly exchange upstream PNs. The total number of claws and PN connections are maintained during the shuffling. (**B**) The percent variance distribution of each KC class connectivity with local shuffling. (**C**) Input ratio from the top ten glomeruli under different local shuffling radii for each KC class.

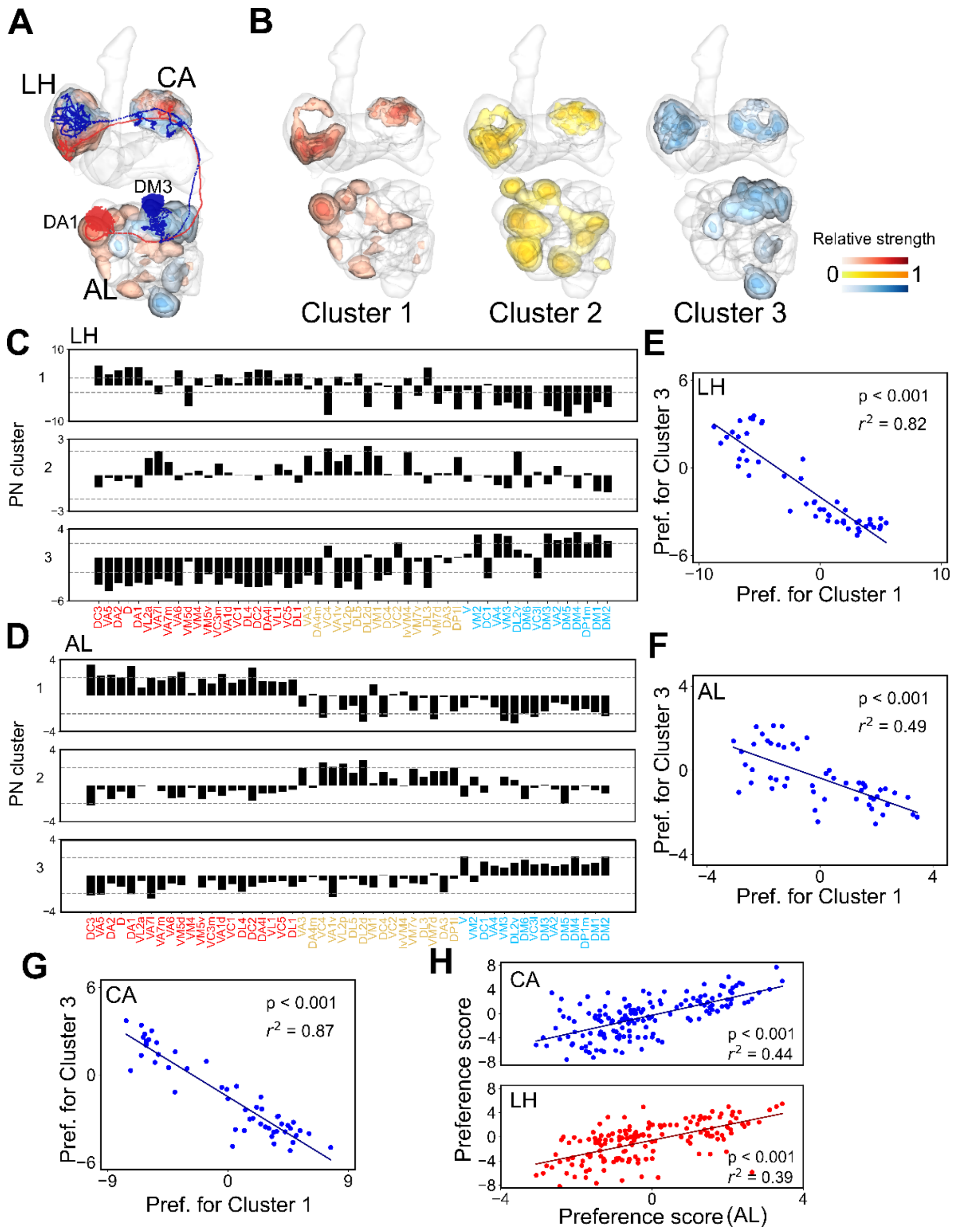

Fig. S21. Synaptic spatial segregation of PN clusters across the olfactory network architecture (**A**) The synaptic density maps of cluster 1 and cluster 3 PNs with example neurons. (**B**) The synaptic density maps of PN clusters. (**C-D**) The spatial innervation preference of each glomerulus for three PN clusters. Ordinates indicate the differences in preference between the real and 30 shuffled networks in LH (C) and AL (D). (**E-G**) The regression plot of the preference score of each glomerulus for PN cluster 1 (x-axis) and PN cluster 3 (y-axis) in LH (E), AL (F), and CA (G). (**H**) Correlation of synaptic spatial innervation preference between each glomerulus and each PN cluster (for the comparison between AL and CA, $r^2 = 0.44$, $p < 0.001$; for the comparison between AL and LH, $r^2 = 0.39$, $p < 0.001$).

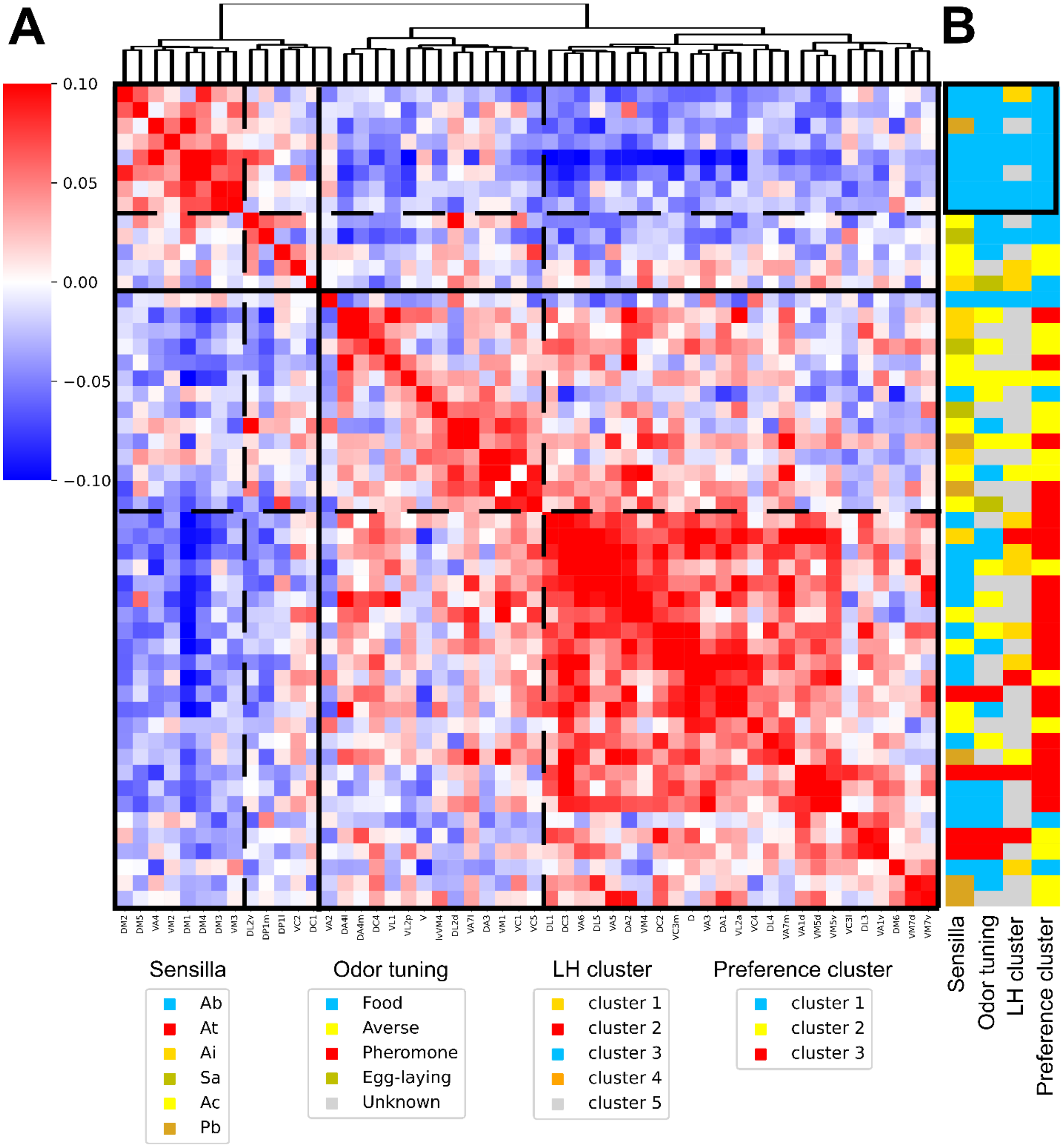

Fig. S22. Correlation between projection and physiology for AL glomeruli. (**A**) The correlation matrix of glomerular projection. The color of each element in the matrix represents the similarity (Pearson correlation) of the targeting KCs between glomeruli. Hierarchical clustering analysis is performed to visualize the grouping of glomerular subsets. (**B**) The morphological and functional features of glomeruli are color-labeled on the right-hand side of the matrix, including the preference cluster (Fig. 1B), the sensilla type of glomerular upstream ORNs *(40)*, and the glomerular axonal cluster in LH *(18)*. A group of glomeruli (black rectangle) with similar projection targets shares similar physiological characteristics.

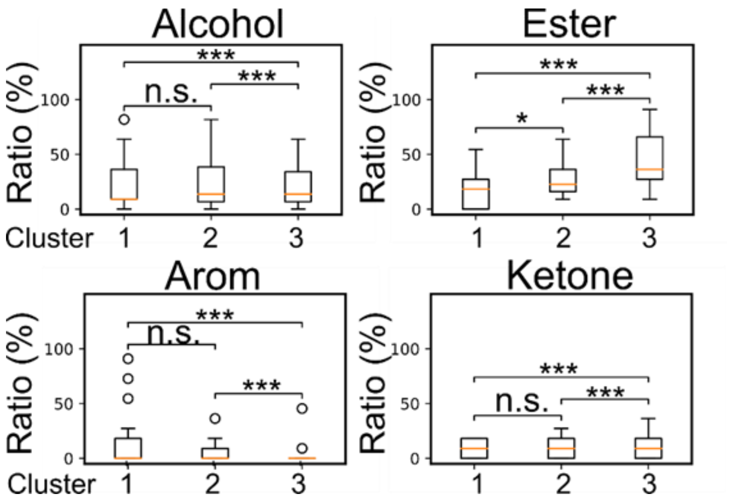

Fig. S23. The tuning profile of the glomerular cluster. We counted the ratio of glomeruli that are sensitive to an odor (alcohol, ester, arom, or ketone) in each PN cluster. A glomerulus is defined as sensitive to an odor if it is among the top ten most responsive odors of that glomerulus (data from the DoOR database *(45)*). (The Mann-Whitney U test: n.s.: not significant, *: $p < 0.05$, **: $p < 0.01$, and ***: $p < 0.001$.)

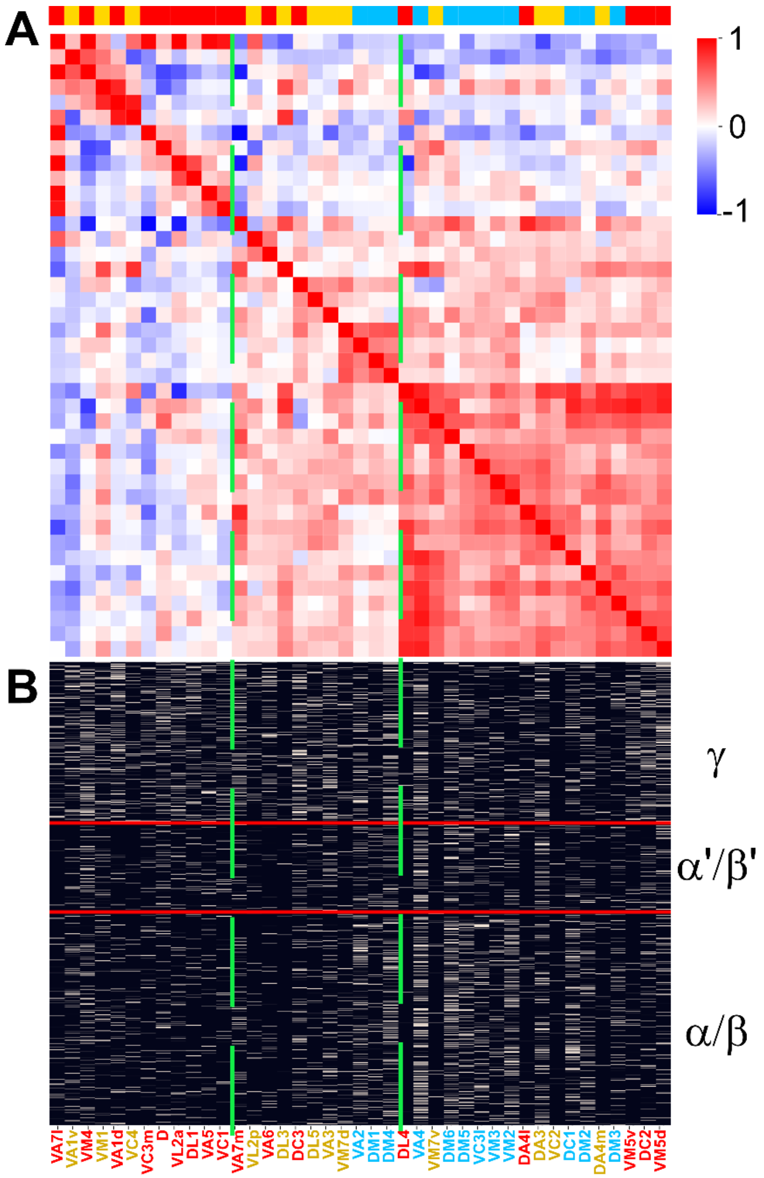

Fig. S24. Connection matrix based on functional responses. (**A**) Glomerular tuning profile correlation matrix as shown in Fig. 3B. (**B**) Connection matrix with glomeruli re-arranged based on the correlation matrix in (A). The top row color and the color of glomeruli at the bottom indicate the connection preferences shown in Fig. 1B.

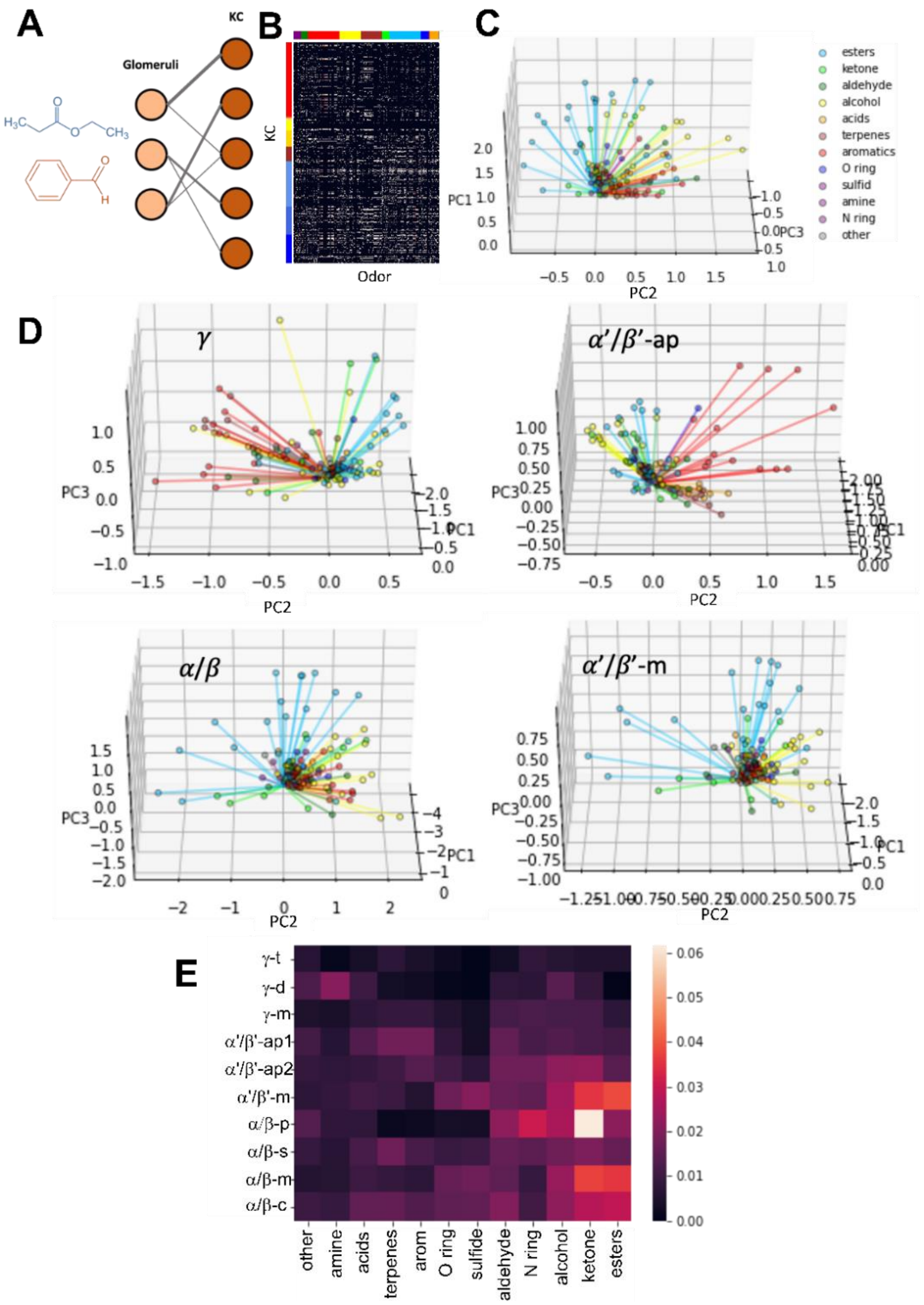

Fig. S25. Olfactory response simulation of KCs and MB lobes. (**A**) The schematic illustration of odor representation from AL glomeruli to KCs. The KC responses to an odor is simulated by multiplying the glomeruli-to-KC connection number by the glomeruli response level induced by a given odor. The KC response level is normalized by the total claw number of each KC. (**B**) The table of simulated KC response to ~700 odors. (**C**) The first three principal components in PCA of the simulated odor representation of KCs. Each dot in the plot represents an odor and is color-labeled according to the functional group the odor possesses. (**D**) Similar to (B) but for KCs in γ, α'/β'-ap, α'/β'-m, or α/β lobe, respectively. The angular difference between each two molecules represents the KC responsive pattern dissimilarity. (**E**) The chemical sensitivity of KC subclasses is computed by summing the odor responses of KCs in each subclass.

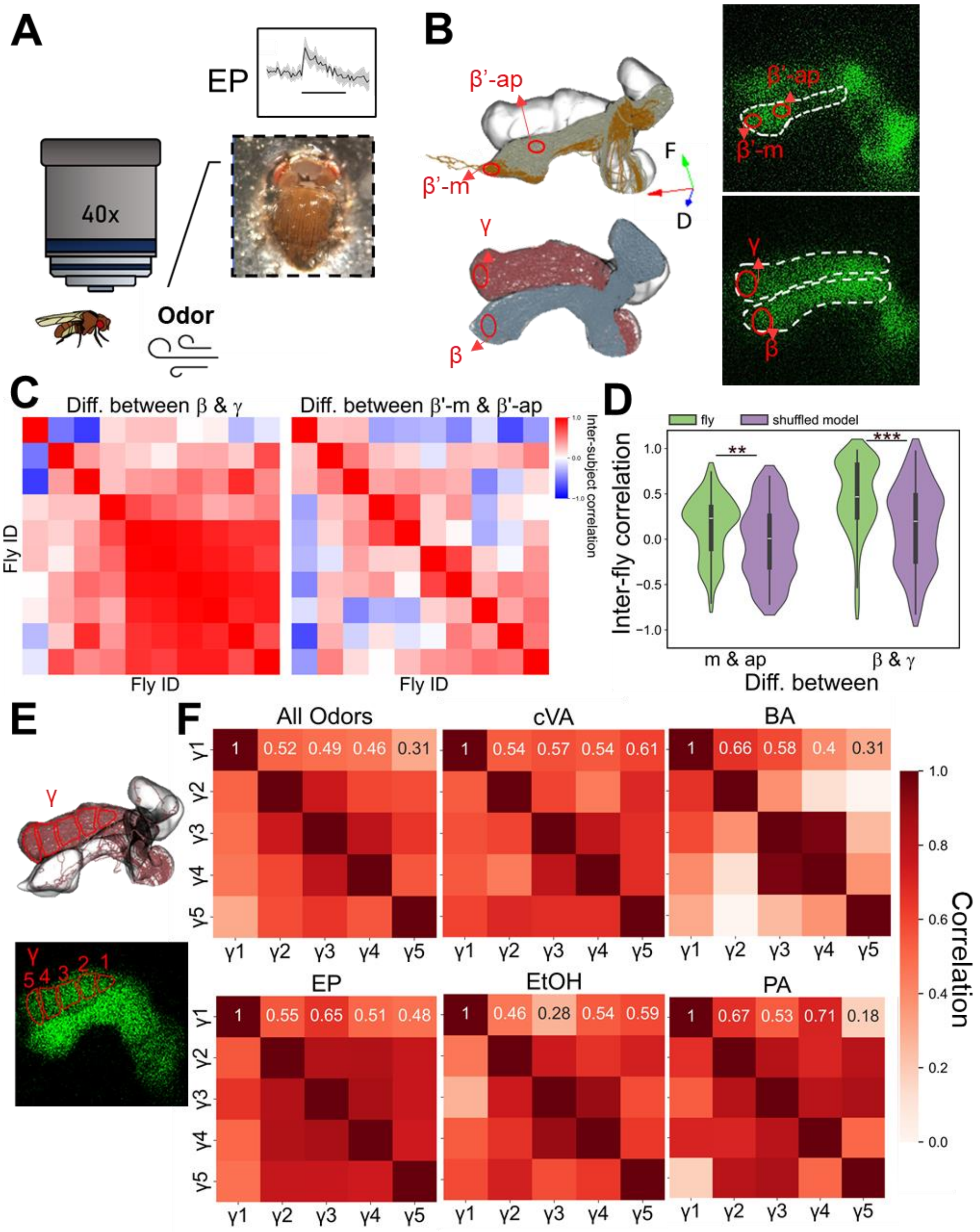

Fig. S26. Functional imaging of odor-evoked responses in MB lobes. (**A**) Schematic representation of the functional imaging experiments. Inset: Example calcium response recorded in the β lobe, with the bar indicating the odor delivery period. (**B**) Recording regions of interest (ROIs) in each lobe: β'-ap, β'-m, β, and γ. (**C**) Correlations of response differences between lobes across flies. (**D**) Comparison of correlations between flies and shuffled models (n = 30), showing significantly higher correlations across flies than in shuffled models (Mann-Whitney test: *: $p < 0.05$, **: $p < 0.01$, and ***: $p < 0.001$). (**E**) Schematic illustration of imaging across γ sub-regions. (**F**) Correlation analysis of odor responses among γ1 to γ5 sub-regions (*n* = 8).

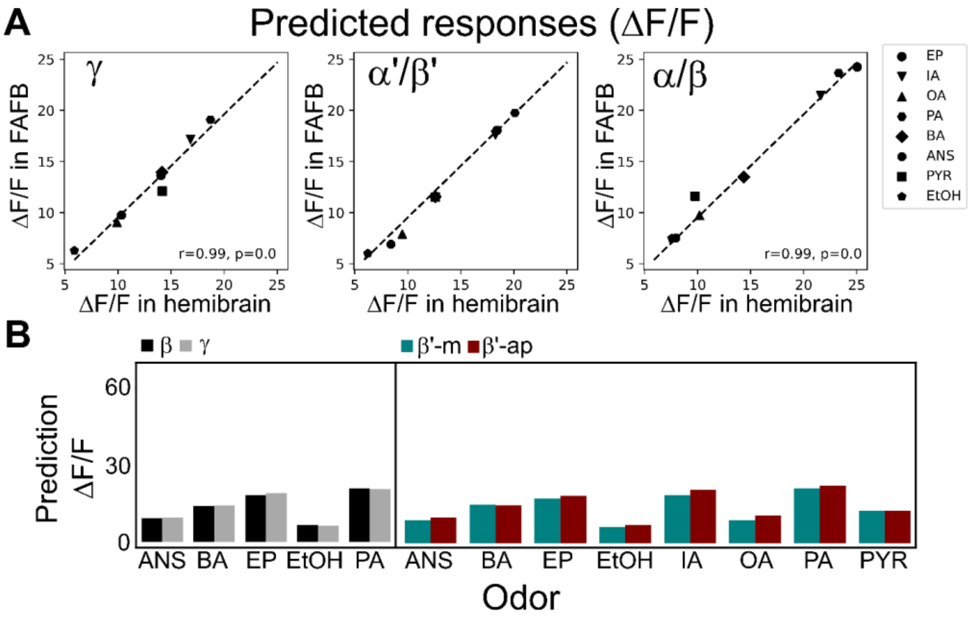

Fig. S27. Odor response predictions in shuffled networks and FAFB network. (**A**) Comparison of odor response predictions across three KC classes between the hemibrain network and the FAFB network. (**B**) Simulated odor response predictions using shuffled random networks, following the same approach as in Fig. 3C.

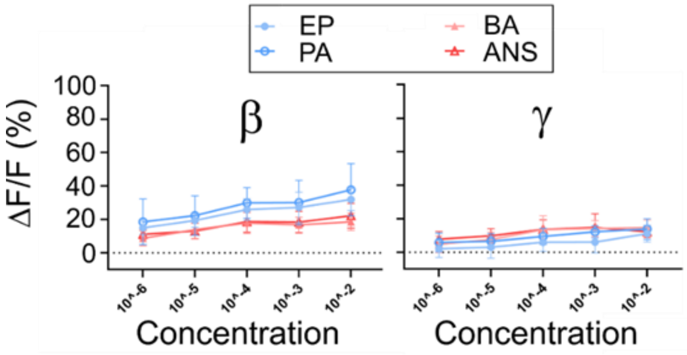

Fig. S28. Distinct response levels in different MB lobes induced by varying gradients of esters and aromatics. The odor-evoked response levels of MB lobes were analyzed across six orders of magnitude of odor concentration. Ethyl propionate (EP) and pentyl acetate (PA) elicited significantly higher responses than benzaldehyde (BA) and ammonium sulfide (ANS) in the β lobe at certain concentrations, while ANS and BA evoked significantly higher responses than EP and PA in the γ lobe at other concentrations. (Two-way ANOVA with Bonferroni correction: n.s.: not significant, *: $p < 0.05$, **: $p < 0.01$, and ***: $p < 0.001$; see Table S10 for detailed statistics.)

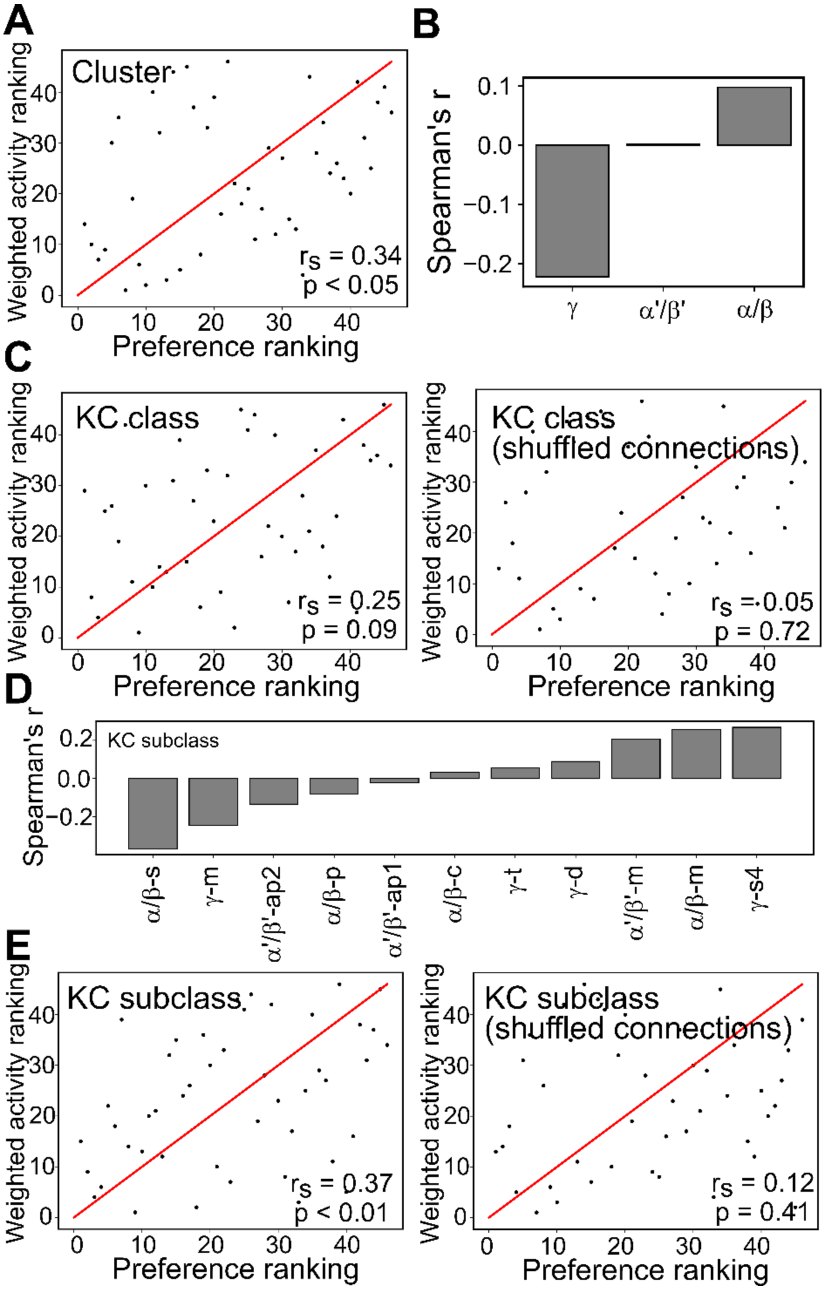

Fig. S29. Correlation between behavioral odor preferences and PN/KC activity. (**A**) Total weighted activity of PN clusters correlates with behavioral odor preferences (Spearman's correlation test: $r_s = 0.34$, $p < 0.05$). (**B**) Correlation coefficient assessed based on KC class activity (see Methods). (**C**) Same as (A), but calculated for KC classes using PN-to-KC connectomic data from the hemibrain dataset (left) and a shuffled network (right) (Spearman's correlation test: $r_s = 0.25$, $p = 0.09$ for the hemibrain dataset; $r_s = 0.05$, $p = 0.72$ for a shuffled network). (**D**) Same as (B), but calculated for KC subclasses. (**E**) Same as (C), but calculated for KC subclasses using data from the hemibrain dataset (left) and a shuffled network (right) (Spearman's correlation test: $r_s = 0.37$, $p < 0.01$ for the hemibrain dataset; $r_s = 0.12$, $p = 0.41$ for a shuffled network).

Table S1. The classification of glomeruli based on the PN projection anatomy in LH *(18)*.

**Group 1**	1, D, DC1, DC2, DL1, DL5, DM2, DM6, VA6, VC2, VL2p, VM7<br>(1 was not collected in the hemibrain dataset.)
**Group 2**	DA1, DC3, DL3, VA1d, VA1v, VA3
**Group 3**	DM1, DM3, DM5, DP1m, VA2, VM2, VM3, DL6<br>(DL6 was not collected in the hemibrain dataset.)
**Group 4**	VA7l, VA7m, VL2p, VM1
**Group 5**	DA1, DA3, VA1v, VL1

Table S2. The classification of glomeruli based on the PN projection anatomy in MB (*18, 20*).

**Group 1**	DM2, DM5, DP1m, VA7m, VL2p, VM2, VM3
**Group 2**	VA1d, VA7l, VC2, VL2p
**Group 3**	DA1, DL3, DL6, VM7 (DL6 was not collected in the hemibrain dataset.)
**Group 4**	1, D, DC2, DC3, DL1, DM6, VA1v (1 was not collected in the hemibrain dataset.)

Table S3. The classification of glomeruli based on their sensilla *(40)*.

**LB (ab)**	DM1, DM2, DM4, DM5, V, VA2
**TB (ab)**	D, DL4, DM6, VA3, VC4, VM2, VM3, VM5v
**SB (ab)**	DA2, VA6, VA5, DM3, DL5, DL1
**pb**	1, VA4, VA7l, VC1, VC2, VM7 (1 was not collected in the hemibrain dataset.)
**ac**	DC4, DL2, VC3, VL1, VL2a, VM1, VM4, VM6 (DL2 and VC3 have two subgroups, while VM6 was not collected in the hemibrain dataset.)
**T3**	DA4l, DA4m, DC1, DL3, VA1d, VA1v
**T2**	DA3, DC3
**T1**	DA1

Table S4. The classification of glomeruli based on their functionality *(22)*.

**Food**	DC3, DL2d, DL2v, DM1, DM2, DM3, DM4, *DM5, DM6, DP1l, DP1m, VA2, VA4, VA6, VC3l, VC3m, VL2a, VM1, VM2, VM3, VM5d, VM5v, VM7d
**Averse**	V, DA2, DA4l, DC2, DC4, DL4, DL5, VA7l, VL2p
**Pheromone**	DA1, DL3, VA1d, VA1v
**Egg-laying**	DC1, VC5
**Unknown**	D, DA3, DA4m, DL1, VA3, VA5, VA7m, VC1, VC2, VC4, VL1, VM4, VM7v, lvVM4

*According to the original paper *(65)*, we regard DM5 as food-responsive glomerulus

Table S5. The community structure derived from the FAFB dataset (*22*).

**Core**	DM2, DP1m, VM2, DL2v, DM3, DM4, DM1, VM3, VA2, VA4
**2nd**	DM6, DM5, DC1, DL2d, VC4, VC3l, DP1l, VA6, VM5d
**Under-convergent**	VA7m, VP1, VP2, V, VL1, DA4m, DA2, DA3, DA1<br>(VP1 and VP2 were not considered in our analysis)
**Others**	DL1, DC3, VA7l, VL2a, DC2, VM1, D, VM4, VA1d, VM7v, VA5, VC3m, VC1, DL3, DL5, VA3, DC4, VM7d, VC5, VA1v, DA4l, VL2p, DL4, VM5v, VC2, VP3 (VP3 was not considered in our analysis)

Table S6. The glomeruli associated with aversive or attractive responses based on 12 aversive and attractive odors *(52)*.

**Averse**	DL5, D, DL1, DA4, DC3
**Attract**	DM4, DM5, DM2

Table S7. The classification of glomeruli based on the innervation overlap between PN and KC *(17)*.

**γ**	DL1, VL2a, VA5, VA7l, DC2
**α'/β'**	DM1, VA4
**α/β-p**	DL4
**α/β-s**	DL3, DA1, VA1d, D
**α/β-c**	DM2

Table S8. The classification of glomeruli based on the neuronal distance *(30)*.

**1**	DL5, VP4, VP1l
**2**	VM4, VC5, VM5d, VM6, DL5, VC3, DC4, VP5
**3**	DL2v, DL2d
**4**	VM4, VL1, VP2
**5**	VC5, DA4m, V, VP4, VP2, VP1d, VP1m
**6**	VM1, VL1, DM5, VA4, VC2, DA3, VA7m, DP1l, DP1m, VL2p, DA2, DA4l, VC1, VA5, VA7l, DA1, VP2
**7**	DL2v, DM5, DM1, DM4, VM2, DM2, DA2, DM3
**8**	VL2a, DC1, D, DL4, VA1v, DA1, DC3, VA1d
**9**	VM1, VM5d, VA3, DA3, DL1, VM7v, VC4, VA2, DA2, D, DC2, VA6, DL3
**10**	DM6, VM5d, VM5v, VM7v, VC3, DL3

Table S9. Glomeruli and associated receptors with the most responded odor (from the DoOR database *(45)*). The glomeruli are arranged in the hierarchical order, shown in Fig. 3

DM2	Or22a	ethyl hexanoate	VC5	lr75d	pyrrolidine
DM5	Or85a	ethyl 3-hydroxybutyrate	DL1	Or10a	methyl salicylate
VA4	Or85d	ethyl pentanoate	DC3	Or83c	farnesol
VM2	Or43b	ethyl trans-2-butanoate	VA6	Or82a	geranyl acetate
DM1	Or42b	ethyl acetate	DL5	Or7a	E2-hexenal
DM4	Or59b	methyl acetate	VA5	Or49b	2-methylphenol
DM3	Or47a	pentyl acetate	DA2	Or56a	nan
VM3	Or9a	3-hydroxy-2-butanone	VM4	lr76a	nan
DL2v	lr75abc	acetic acid	DC2	Or13a	1-octen-3-ol
DP1m	lr64a	2,3-butanediol	VC3m	lr41a	putrescine
DP1l	lr75abc	acetic acid	D	Or69a	ethyl 3-hydroxy hexanoate
VC2	Or35a	1-heptanol	VA3	Or67b	acetophenone
DC1	Or19a	valencene	DA1	Or67d	nan
VA2	Or92a	2,3-butanedione	VL2a	lr84a	phenylacetaldehyde
DA4l	Or43a	1-hexanol	VC4	lr75d	pyrrolidine
DA4m	Or2a	3-hydroxy-2-butanone	DL4	Or49a	2-heptanone
DC4	lr64a	acetic acid	VA7m	Or33c	cyclohexanone
VL1	lr75d	pyrrolidine	VA1d	Or88a	pyrrolidine
VL2p	lr31a	2-oxovaleric acid	VM5d	Or85b	butyl acetate
V	Or21a	carbon dioxide	VM5v	Or98a	ethyl benzoate
lvVM4	lr40a	nan	VC3l	Or67c	ethyl lactate
DL2d	lr75abc	acetic acid	DL3	Or65a	pyrrolidine
VA7l	Or46aA	4-methylphenol	VA1v	Or47b	(S)-(+)-carvone
DA3	Or23a	1-pentanol	DM6	Or67a	butyl propanoate
VM1	lr92a	dimethylamine	VM7d	Or42a	propyl acetate
VC1	Or71a	4-ethyl guaiacol	VM7v	Or59c	ethyl butyrate

Table S10. Statistics for odor gradient experiment for MB lobes

Odor	Odor	Concentration	β lobe	γ lobe
EP	PA	10^{-6}	n.s.	n.s.
EP	BA	10^{-6}	*	n.s.
EP	Anisole	10^{-6}	n.s.	n.s.
PA	BA	10^{-6}	n.s.	n.s.
PA	Anisole	10^{-6}	n.s.	n.s.
BA	Anisole	10^{-6}	n.s.	n.s.
EP	PA	10^{-5}	n.s.	n.s.
EP	BA	10^{-5}	n.s.	n.s.
EP	Anisole	10^{-5}	*	n.s.
PA	BA	10^{-5}	n.s.	n.s.
PA	Anisole	10^{-5}	n.s.	n.s.
BA	Anisole	10^{-5}	n.s.	n.s.
EP	PA	10^{-4}	n.s.	n.s.
EP	BA	10^{-4}	n.s.	n.s.
EP	Anisole	10^{-4}	n.s.	*
PA	BA	10^{-4}	*	n.s.
PA	Anisole	10^{-4}	*	n.s.
BA	Anisole	10^{-4}	n.s.	n.s.
EP	PA	10^{-3}	n.s.	n.s.
EP	BA	10^{-3}	*	*
EP	Anisole	10^{-3}	n.s.	n.s.
PA	BA	10^{-3}	n.s.	n.s.
PA	Anisole	10^{-3}	n.s.	n.s.
BA	Anisole	10^{-3}	n.s.	n.s.
EP	PA	10^{-2}	n.s.	n.s.
EP	BA	10^{-2}	***	n.s.
EP	Anisole	10^{-2}	*	n.s.
PA	BA	10^{-2}	*	n.s.
PA	Anisole	10^{-2}	n.s.	n.s.
BA	Anisole	10^{-2}	n.s.	n.s.
